# Distinct Convergent Brain Alterations in Sleep Disorders and Sleep Deprivation: A Meta-Analysis

**DOI:** 10.1001/jamapsychiatry.2025.0488

**Published:** 2025-04-23

**Authors:** Gerion M. Reimann, Alireza Hoseini, Mihrican Koçak, Melissa Beste, Vincent Küppers, Ivana Rosenzweig, David Elmenhorst, Gabriel Natan Pires, Angela R. Laird, Peter T. Fox, Kai Spiegelhalder, Kathrin Reetz, Simon B. Eickhoff, Veronika I. Müller, Masoud Tahmasian

**Affiliations:** 1 Institute of Neuroscience and Medicine, Brain and Behaviour (INM-7), Research Centre Jülich, Jülich, Germany; 2 Section of Translational Neurodegeneration, Department of Neurology, RWTH Aachen University, Aachen, Germany; 3 Department of Neurology, School of Medicine, Mashhad University of Medical Sciences, Mashhad, Iran; 4 Faculty of Medicine, Bahcesehir University, Istanbul, Türkiye; 5 Institute for Systems Neuroscience, Medical Faculty and University Hospital Düsseldorf, Heinrich Heine University Düsseldorf, Düsseldorf, Germany; 6 Department of Nuclear Medicine, Faculty of Medicine and University Hospital Cologne, University of Cologne, Cologne, Germany; 7 Sleep and Brain Plasticity Centre, Department of Neuroimaging, Institute of Psychiatry, Psychology and Neuroscience, King’s College London, London, United Kingdom; 8 Sleep Disorders Centre, Guy’s and St Thomas’ NHS Foundation Trust, London, United Kingdom; 9 Institute of Neuroscience and Medicine, Molecular Organization of the Brain (INM-2), Research Centre Jülich, Jülich, Germany; 10 Departamento de Psicobiologia, Escola Paulista de Medicina, Universidade Federal de São Paulo, São Paulo, Brazil; 11 Department of Physics, Florida International University, Miami; 12 Research Imaging Institute and Department of Radiology, Long School of Medicine, University of Texas Health Science Center at San Antonio, San Antonio; 13 Department of Psychiatry and Psychotherapy, Medical Centre–University of Freiburg, Faculty of Medicine, University of Freiburg, Freiburg, Germany

## Abstract

**Importance:**

Sleep disorders have different etiologies yet share some nocturnal and daytime symptoms, suggesting common neurobiological substrates; healthy individuals undergoing experimental sleep deprivation also report analogous daytime symptoms. However, brain similarities and differences between long-term sleep disorders and short-term sleep deprivation are unclear.

**Objective:**

To investigate the shared and specific neural correlates across sleep disorders and sleep deprivation.

**Data Sources:**

PubMed, Web of Science, Embase, Scopus, and BrainMap were searched up to January 2024 to identify relevant structural and functional neuroimaging articles.

**Study Selection:**

Whole-brain neuroimaging articles reporting voxel-based group differences between patients with different sleep disorders and healthy control participants or between total or partial sleep-deprived and well-rested individuals were included.

**Data Extraction and Synthesis:**

Significant coordinates of group comparisons, their contrast direction (eg, patients < controls), and imaging modality were extracted. For each article, 2 raters independently evaluated eligibility and extracted data. Subsequently, several meta-analyses were performed with the revised activation likelihood estimation algorithm using *P* < .05 cluster-level familywise error correction.

**Main Outcomes and Measures:**

Transdiagnostic regional brain alterations were identified across sleep disorders and among articles reporting sleep deprivation. Their associated behavioral functions and task-based or task-free connectivity patterns were explored using 2 independent datasets (BrainMap and the enhanced Nathan Kline Institute–Rockland Sample).

**Results:**

A total of 231 articles (140 unique experiments, 3380 unique participants) were retrieved. The analysis across sleep disorders (n = 95 experiments) identified the subgenual anterior cingulate cortex (176 voxels, *z* score = 4.86), associated with reward, reasoning, and gustation, and the amygdala and hippocampus (130 voxels, *z* score = 4.00), associated with negative emotion processing, memory, and olfaction. Both clusters had positive functional connectivity with the default mode network. The right thalamus (153 voxels, *z* score = 5.21) emerged as a consistent regional alteration following sleep deprivation (n = 45 experiments). This cluster was associated with thermoregulation, action, and pain perception and showed positive functional connectivity with subcortical and (pre)motor regions. Subanalyses regarding the direction of alterations demonstrated that the subgenual anterior cingulate cortex exhibited decreased activation, connectivity, and/or volume, while the amygdala and hippocampus cluster and the thalamus cluster demonstrated increased activation, connectivity, and/or volume.

**Conclusions and Relevance:**

Distinct convergent brain abnormalities were observed between long-term sleep disorders (probably reflecting shared symptoms) and short-term sleep deprivation.

## Key Points

**Question:**

Are there any shared or specific structural and functional brain alterations of long-term sleep disorders and short-term sleep deprivation?

**Findings:**

In multimodal neuroimaging meta-analyses across sleep disorders, convergent regional abnormality was observed in the bilateral subgenual anterior cingulate cortex and the right amygdala and hippocampus. The right thalamus was consistently altered following sleep deprivation in healthy individuals.

**Meaning:**

Distinct convergent neural alterations between long-term sleep disorders and short-term sleep deprivation were observed, highlighting their unique underlying neurobiological substrates.

## Teaser

This meta-analysis investigates the shared and specific neural correlates across sleep disorders and sleep deprivation.

## Introduction

Chronic sleep disorders and short-term sleep loss are among the most frequent conditions in the general population, yet they are frequently overlooked in public health.^1^ Poor sleep and sleep disturbances are risk factors for various mental health conditions^2,3^ and are frequently reported by patients with a wide range of neuropsychiatric disorders,^4,5^ making them a global problem.^6^ Recent neuroimaging advancements have enhanced our understanding of the relationship between poor sleep and the brain. However, some critical questions remain. What are the consistent neural correlates of long-term sleep disorders and short-term sleep loss? Do these conditions involve overlapping or unique neurobiological substrates? This meta-analysis aims to address these questions.

The *International Classification of Sleep Disorders, Third Edition* classifies sleep disorders into 7 categories,^7^ while the *Diagnostic and Statistical Manual of Mental Disorders, Fifth Edition* suggests 10.^8^ Some studies also found distinct subtypes in each sleep disorder (eg, insomnia disorder^9,10,11^), which together reflect that differential diagnosis of sleep disorders and their subtyping are still under debate.^12^ Several etiologies cause chronic sleep disorders, and patients with them report a wide range of nocturnal and daytime symptoms. However, some symptoms, such as sleep duration or efficiency dissatisfaction, sleep fragmentation, daytime sleepiness, and cognitive or mood dysfunctions are similar across different sleep disorders.^6,7^ In addition, large-scale genome-wide association studies identified shared polymorphisms and associated genomic loci and pathways across multiple sleep-related symptoms.^13,14^ Hence, the evidence of homologous symptoms, overlapping comorbidities, and common genetic underpinnings suggests potential shared pathophysiological substrates across diverse sleep disorders.

Short-term sleep loss is highly prevalent in the general population due to constant societal demands and the pervasive use of social media, particularly among adolescents and young adults.^15^ Acute total sleep deprivation (ie, >24 hours) or partial sleep restriction over a few nights in healthy adults drastically impacts brain development, waste clearance, emotional stability, attention, working memory, and school and work performance.^16,17^ However, the neurobiological understanding of short-term sleep deprivation remains obscure.

Existing neuroimaging studies mainly included small samples and demographically and clinically heterogeneous populations and used divergent tasks, preprocessing steps, and statistical approaches. This calls for consolidating the literature to overcome the heterogeneity. Systematic reviews highlighted shared abnormalities between obstructive sleep apnea (OSA), insomnia disorder, and depressive symptoms in the default mode network (DMN) and salience network.^18,19^ Moreover, previous meta-analyses focused on either individual sleep disorders (eg, insomnia disorder, OSA, narcolepsy)^20,21,22,23,24,25^ or total sleep deprivation,^26,27,28^ chose various statistical approaches, and were often limited to unimodal imaging studies, leading to divergent findings. Thus, a comprehensive and methodologically rigorous multimodal coordinate-based meta-analysis (CBMA) is needed to understand the potential similarities and differences in the neurobiology of sleep disorders and experimental sleep deprivation. Herein, we performed a multimodal CBMA using whole-brain structural and functional studies to find where in the brain the amount of convergence between reported foci is more than expected by chance. First, we performed 2 activation likelihood estimation (ALE) analyses testing for regional convergence across different sleep disorders and across total and partial sleep deprivation to identify altered patterns of long-term sleep disorders and short-term sleep loss. Subsequently, we (1) explored conjunction and contrast analyses to identify shared and specific alterations between 2 conditions; (2) characterized the function of obtained convergent regions in the ALE analyses using the BrainMap database; (3) assessed the task-based and task-free connectivity patterns of those regions to identify the connected brain areas or networks; and (4) performed several complementary ALE subanalyses for each disorder or experimental condition, imaging modality, and contrast direction as well as further consistency analyses to address heterogeneity.

## Methods

### Literature Search and Article Selection

**Figure 1.**
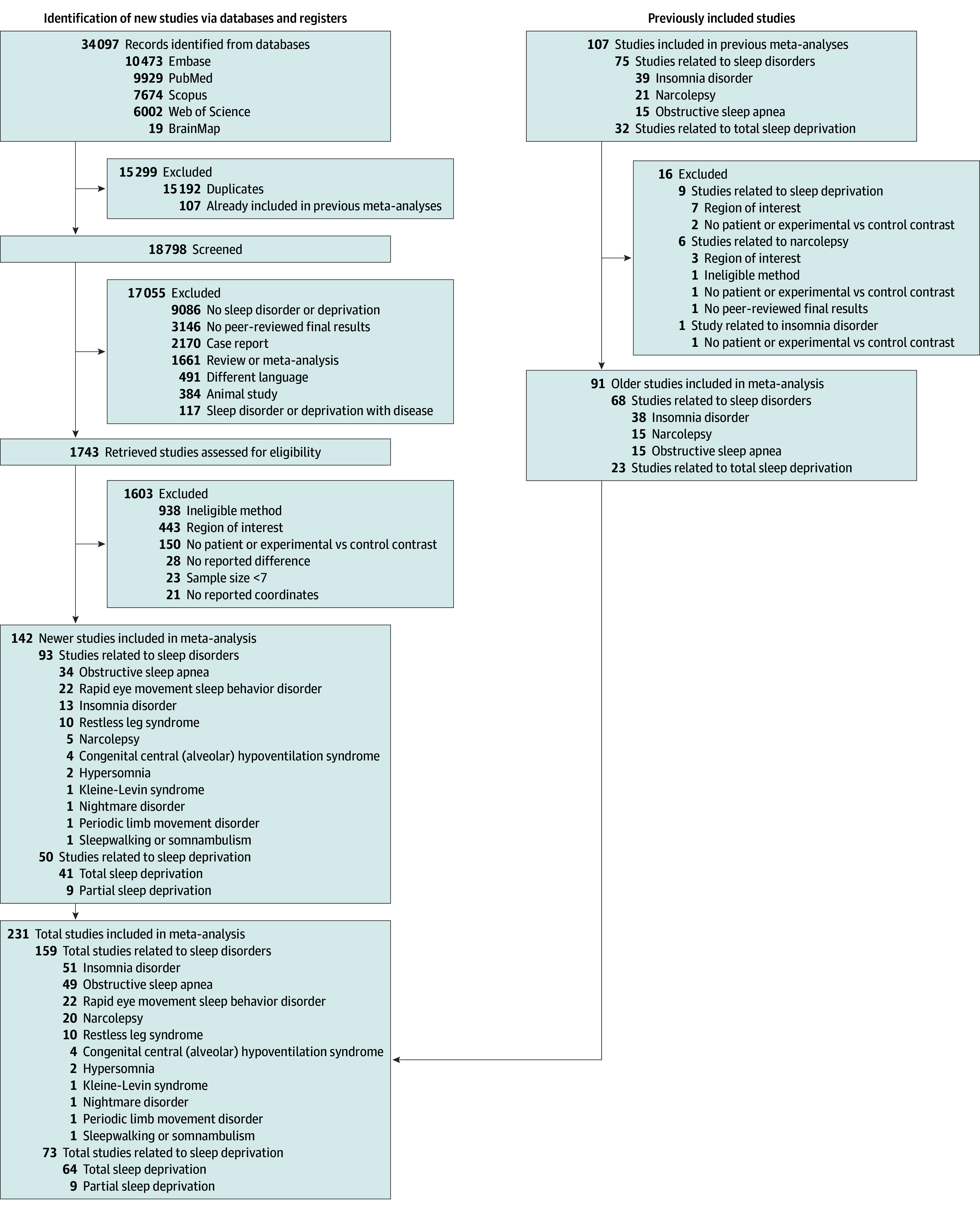
PRISMA Flow Diagram In total, we included 231 studies, 4 of which involved more than 1 disorder or sleep deprivation (2 studied narcolepsy and hypersomnia, 1 studied total sleep deprivation and insomnia disorder, and 1 studied obstructive sleep apnea and periodic limb movement disorder). Due to overlapping categorizations, studies may appear in more than 1 group (eg, disorder, deprivation type). The reported numbers reflect how many studies belong to each condition rather than mutually exclusive subsets. Additionally, the 2 studies addressing both narcolepsy and hypersomnia are counted under both older studies (91) for narcolepsy and newer studies (142) for hypersomnia but are counted once for the total number (231).

We performed a thorough literature search across a wide range of sleep disorders and total or partial sleep deprivation neuroimaging studies in several databases up to January 2024. Standard screening procedures were followed, including reference tracing, incorporation of databases from our previous meta-analyses, duplicate removal, and an initial screening of titles and abstracts, followed by full-text screening based on predefined selection criteria (Figure 1). The authors were contacted for clarification when necessary. Multiple authors were involved at each screening stage to ensure an unbiased screening process. We included peer-reviewed group comparison studies with statistically significant findings using structural (ie, gray matter volume) or functional (ie, resting-state functional magnetic resonance imaging [rs-fMRI], task-based functional magnetic resonance imaging [t-MRI], or glucose metabolism positron emission tomography [PET]) whole-brain imaging that compared patients with diagnosed sleep disorder vs healthy control participants or compared experimentally sleep-deprived individuals vs well-rested ones, considering participants of all age groups. Details of the search terms and screening process are provided in the eMethods in Supplement 1, and detailed exclusion criteria are listed in eTable 1 in Supplement 1. Our search strategy adhered to the current Preferred Reporting Items for Systematic Reviews and Meta-Analyses (PRISMA) reporting guidelines, the Enhancing the Quality and Transparency of Health Research (EQUATOR) guidelines, and best-practice recommendations for neuroimaging meta-analyses.^29,30,31^ This process included preregistration at the International Prospective Register of Systematic Reviews (PROSPERO Identifier: CRD42022343369).

### Experiment Merging

Hereinafter, a single publication is called an *article*, while an *experiment* describes a set of stereotactic coordinates of a comparison between a patient or experimental group and a control group originating from a specific analysis. We merged experiments based on identical or overlapping samples (reported within or between studies) into a single merged experiment to reduce within-group effects and to prevent these samples from disproportionately influencing the results (eMethods in Supplement 1).^32^

### Statistical Analysis

#### ALE Meta-Analysis

We used the revised ALE algorithm^33^ to identify convergent regional alterations across the existing literature (eMethods in Supplement 1). First, 2 main ALE analyses were conducted, once across all sleep disorders and once across total and partial sleep deprivation experiments. Furthermore, we conducted conjunction and contrast analyses between our 2 main ALE analyses to examine shared or specific convergence between short- and long-term sleep conditions (eMethods in Supplement 1). Several complementary ALE subanalyses were performed for (1) disorder or condition (each disorder and deprivation type separately); (2) contrast direction (patient or experimental > control [increase] or control > patient or experimental [decrease]); (3) imaging modality (rs-fMRI, t-fMRI, PET), tasks (t-fMRI and task-based PET), functional imaging (rs-fMRI, t-fMRI, and PET), and structural magnetic resonance imaging); and (4) further consistency analyses to address heterogeneity. The latter included restrictions to the following: (1) local voxelwise measures to avoid the ambiguity of global or long-distance connectivity measures in localized alterations (eMethods in Supplement 1)^34^; (2) adult participants (≥18 years) due to developing brain in children; (3) experiments of higher statistical power (n ≥ 21); (4) coordinates corrected for multiple comparisons as more rigorous statistical thresholds; and (5) sleep-deprived participants experiencing wakefulness for approximately 22 to 26 hours, representing the circadian nadir for alertness.

The ALE analyses were performed only if a minimum of 17 experiments could be included to achieve enough statistical power.^35^ The statistical significance threshold was set at a *P* < .001 cluster-forming threshold at voxel level and *P* < .05 cluster-level familywise error correction.^29,30,35^ Moreover, we implemented 2 approaches to ensure the results’ robustness: (1) Bonferroni correction to account for multiple comparisons in the performed subanalyses, given their high number; and (2) fail-safe N^36^ to evaluate the potential publication bias (eMethods in Supplement 1). Finally, we calculated the relative contribution of each experiment for each found region (cluster) (eMethods in Supplement 1). To determine the macrostructural and cytoarchitectural labels of the clusters, we used the SPM Anatomy Toolbox version 3.0 (Forschungszentrum Jülich GmbH); for visualization, we used MRIcroGL version 1.2.2 (NeuroImaging Tools & Resources Collaboratory).

#### Behavioral Decoding

We explored the function of the convergent clusters observed in the ALE analyses through behavioral decoding analysis using the BrainMap database (eMethods in Supplement 1).^37^ These results were corrected using false discovery rate–adjusted *P* < .05.

#### Delineating Functional Networks of Convergent Regions

We examined task-dependent and task-independent functional connectivity (FC) to delineate the FC patterns of identified clusters. For task-dependent FC, meta-analytic connectivity modeling (MACM)^38,39^ using the BrainMap database was applied. Additionally, resting-state FC (rsFC) was calculated using rs-fMRI from a sample (n = 192) of the enhanced Nathan Kline Institute–Rockland Sample dataset^40^ with standard preprocessing (eMethods in Supplement 1). Results were corrected for multiple comparisons (*P* < .001, cluster-forming threshold; *P* < .05, cluster-level familywise error corrected). Furthermore, minimum conjunction was used to identify brain areas exhibiting both task-dependent and task-independent FC with each cluster (conjunctive connectivity).^41^ Finally, a second conjunction identified shared connectivity between the clusters (shared connectivity).

## Results

### Included Articles

We retrieved 34 097 unique abstracts. Abstract and full-text screening yielded 231 articles that fulfilled our selection criteria (eResults and eTables 2 and 3 in Supplement 1), encompassing 140 unique experiments and 3380 unique participants after merging the overlapping samples. For sleep disorders, we included 95 experiments comprising 2302 unique participants and 1417 foci. We also included 45 experiments with 1079 unique participants and 962 foci for sleep deprivation. Of note, 91 of these 231 articles were already included in our previous CBMAs.^21,22,23,26^

### Convergent Regional Alterations Across Sleep Disorders

**Figure 2.**
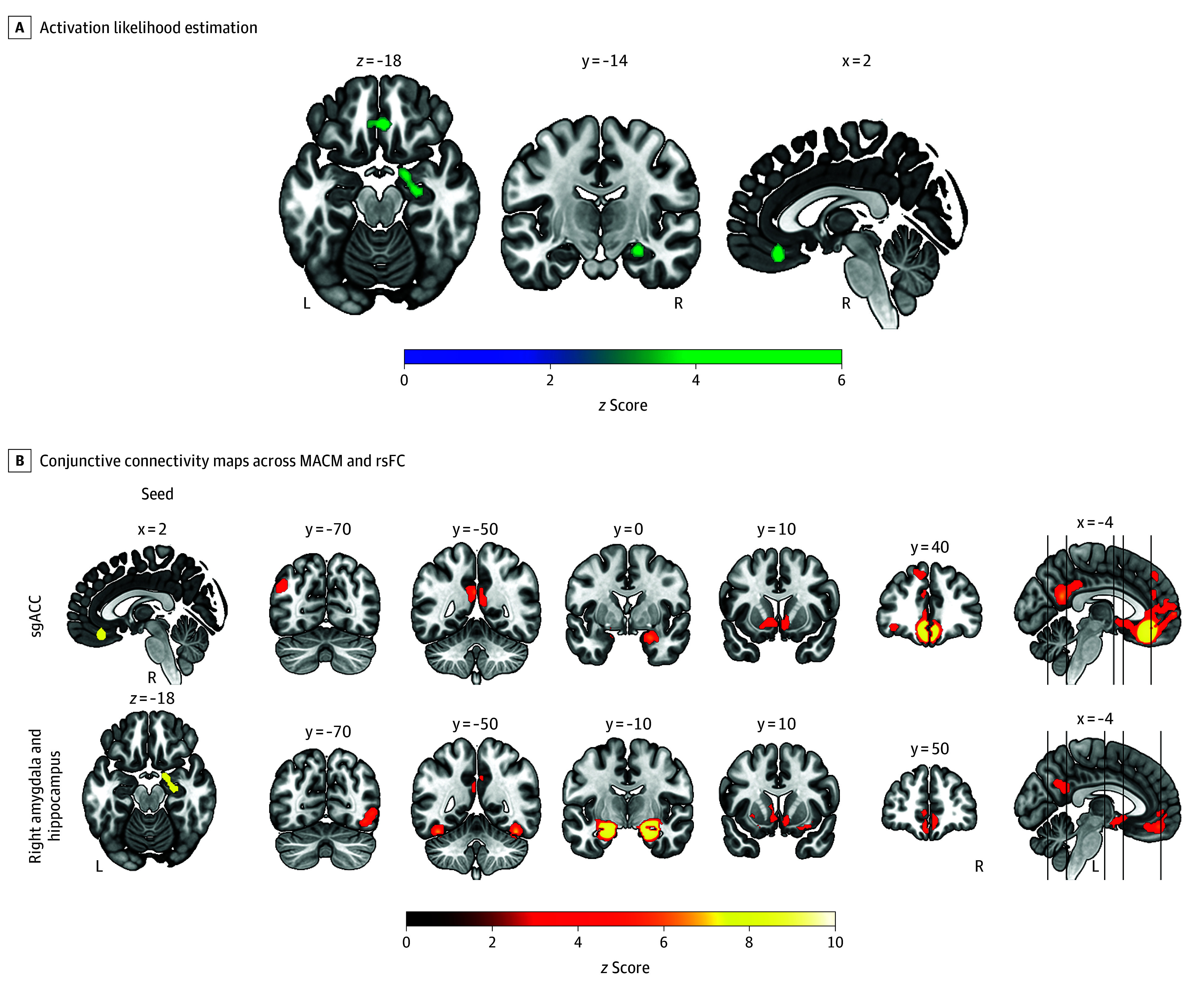
Convergent Regional Alterations Across Sleep Disorders A, Activation likelihood estimation analyses across all sleep disorders identified 2 significant clusters: 1 in the bilateral subgenual anterior cingulate cortex (sgACC) and the other in the right amygdala and right hippocampus. B, Conjunctive connectivity maps were derived from task-based (meta-analytic connectivity modeling [MACM]) and task-free (resting-state functional connectivity [rsFC]) analyses. Coordinates are provided in Montreal Neurological Institute space. The thresholds for activation likelihood estimation, MACM, and rsFC analyses were set at a voxel-level threshold of *P* < .001 and a cluster-level familywise error–corrected *P* < .05. L indicates left; R, right.

Our ALE analysis across all 95 sleep disorders yielded consistent brain alterations in 2 regions: (1) bilateral subgenual anterior cingulate cortex (sgACC) and (2) right amygdala and hippocampus (Table 1 and Figure 2A).

**Table 1.** Convergent Regional Alterations of Main Activation Likelihood Estimation Meta-Analyses

Region	Cluster size, voxels	Peak *z* score	MNI peak coordinates			No.			Macroanatomy^a^	Cytoarchitecture^a^
			x	y	z	Foci	Experiments in analysis	Contributing experiments		
**Sleep disorders**										
Bilateral subgenual anterior cingulate cortex	176	4.86	2	34	−20	1417	95	17	Frontal medial cortex, 61.8%; paracingulate gyrus, 11.7%	Area s32, 48.5%; area s24, 11.9%; area Fo1, 4.6%
Right amygdala and hippocampus	130	4.00	18	−2	−18	1417	95	25	Right amygdala, 46.9%; right hippocampus, 46.4%	Amygdala LB, 21.3%; amygdala SF, 15.1%; hippocampal CA1, 15.0%; amygdala VTM, 12.0%; hippocampal DG, 8.2%
**Sleep deprivation**										
Right thalamus	153	5.21	10	−18	6	962	45	13	Right thalamus, 100%	NA

Abbreviations: CA1, cornu ammonis 1; DG, dentate gyrus; Fo1, frontal gyrus orbital part 1; LB, laterobasal group; MNI, Montreal Neurological Institute; NA, not applicable; s24, subgenual area 24; s32, subgenual area 32; SF, superficial group; VTM, ventromedial part.

^a^ Macroanatomy and cytoarchitecture are based on maximum probability maps of SPM Anatomy Toolbox version 3.0 (Forschungszentrum Jülich GmbH).

The sgACC cluster (176 voxels, *z* score = 4.86) was associated with various disorders, with the first percentage reflecting the relative contribution of each disorder to the cluster and the second percentage reflecting the respective disorder’s proportion in the experiment data: insomnia disorder (48.7%, 27.4%), OSA (30.5%, 26.3%), narcolepsy (11.5%, 13.7%), an experiment involving both OSA and periodic limb movement (5.5%, 1.1%), and rapid eye movement sleep behavior disorder (RBD) (3.8%, 16.8%). Additionally, behavioral decoding revealed that the sgACC cluster was primarily associated with reward processing, perception of gustatory stimuli, and reasoning (eFigure 1 in Supplement 1). The conjunctive connectivity of MACM and rsFC showed that the sgACC was connected with the bilateral amygdala and hippocampus, posterior cingulate cortex (PCC), nucleus accumbens, caudate nucleus, left orbitofrontal cortex, frontal pole, lateral occipital cortex, and precuneus (Figure 2B; eFigure 2 in Supplement 1).

The second consistent cluster across sleep disorders, the right amygdala and hippocampus (130 voxels, *z* score = 4.00), resulted from contribution of insomnia disorder (33.8%, 27.4%), OSA (25.3%, 26.3%), narcolepsy (22.2%, 13.7%), restless legs syndrome (RLS) (6.4%, 6.3%), an experiment with insomnia disorder and OSA (7.5%, 2.1%), RBD (2.4%, 16.8%), and congenital central (alveolar) hypoventilation syndrome (2.4%, 1.1%). The cluster was associated with the processing of negative emotions, such as anger, anxiety, fear, sadness, and disgust, as well as visual perception, respiratory interoception, and memory (eFigure 1 in Supplement 1). The left amygdala and hippocampus, PCC, nucleus accumbens, caudate nucleus, basal forebrain, fusiform gyrus, medial frontal cortex, Broca region, and left orbitofrontal cortex were all connected to the right amygdala and hippocampus cluster based on the conjunction of MACM and rsFC (Figure 2B; eFigure 3 in Supplement 1). Of note, both the sgACC and right amygdala and hippocampus clusters did not survive after adding 30% noise experiments in the fail-safe N analysis (eResults in Supplement 1).

### Convergent Regional Alterations Across Sleep Deprivation

**Figure 3.**
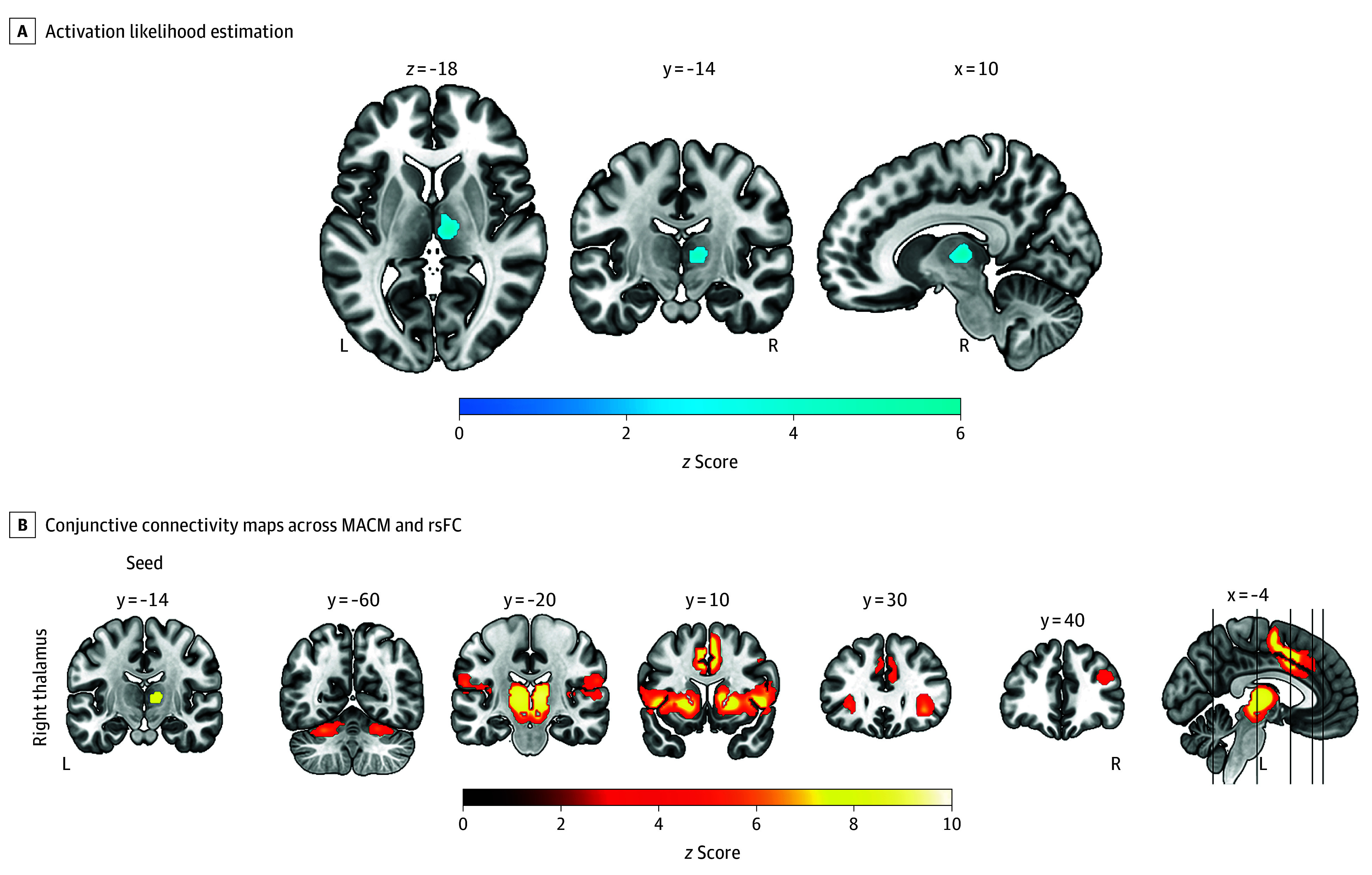
Convergent Regional Alterations Across Sleep Deprivation A, Activation likelihood estimation analyses across total and partial sleep deprivation identified 1 significant cluster in the right thalamus. B, Conjunctive connectivity maps were derived from task-based (meta-analytic connectivity modeling [MACM]) and task-free (resting-state functional connectivity [rsFC]) analyses. Coordinates are provided in Montreal Neurological Institute space. The statistical significance thresholds for activation likelihood estimation, MACM, and rsFC analyses were set at a voxel-level threshold of *P* < .001 and a cluster-level familywise error–corrected *P* < .05. L indicates left; R, right.

A cluster inside the right thalamus (153 voxels, *z* score = 5.21) was found in the ALE analysis across the 45 total and partial sleep deprivation experiments (Table 1 and Figure 3A), surviving after adding 30% noise experiments in the fail-safe N analysis (eResults in Supplement 1), highlighting that this cluster is more stable than the sleep disorder clusters. Both total (82.4%, 84.4%) and partial (17.6%, 15.6%) sleep deprivation contributed. This cluster was located in the ventral-anterior nucleus (56.2%), dorsal-anterior nucleus (32.7%), and ventral-posterior nucleus (11.1%), based on the Melbourne subcortex atlas, scale 2, 7 Tesla.^42^ The right thalamus cluster was associated with thermoregulation, pain perception, and action execution (eFigure 4 in Supplement 1) and functionally connected to the bilateral nucleus accumbens, left thalamus, insula, putamen, caudate nucleus, frontal operculum, dorsal anterior cingulate cortex, paracingulate, presupplementary motor area, inferior parietal cortex, cerebellum VI, right frontal pole, and right amygdala (Figure 3B; eFigure 5 in Supplement 1).

### Conjunction and Contrast Analyses Between Sleep Disorders and Sleep Deprivation

The conjunction ALE confirmed no overlap between the sleep disorder and deprivation clusters. The contrast analysis showed greater convergence in the sgACC and the right amygdala and hippocampus for sleep disorders, while the right thalamus displayed more convergence for sleep deprivation. The shared connectivity map of the sgACC and right amygdala and hippocampus clusters showed overlapping connectivity between these regions, along with connections to the left amygdala and hippocampus, PCC, nucleus accumbens, caudate nucleus, and left orbitofrontal cortex. In contrast, the right thalamus cluster displayed a largely distinct network of functional connections, overlapping with the sleep disorder–associated clusters only in the left nucleus accumbens and caudate nucleus. Additionally, the right thalamus network showed overlap with the right amygdala in the right thalamus and right amygdala and hippocampus map and with the right nucleus accumbens in the right thalamus and sgACC map (eFigure 6 in Supplement 1). These analyses further revealed distinct brain alterations between sleep disorders and sleep deprivation.

### Complementary Subanalyses

Of 46 subanalyses performed, 24 revealed significant clusters (Table 2; eTable 4 in Supplement 1). Key findings include the replication of the sgACC, right amygdala and hippocampus, and right thalamus clusters in various subanalyses across sleep disorders and deprivation as well as the sgACC cluster in insomnia disorder and the right thalamus in total sleep deprivation. Moreover, the contrast-specific analyses elucidated an association of the sgACC cluster with decreased activation, connectivity, and/or volume, while the right amygdala and hippocampus and right thalamus clusters showed the opposite pattern of increase. Further details and additional results, such as modality-specific patterns, are provided in the eResults, eTables 5 to 16, and eFigures 7 to 18 in Supplement 1.

**Table 2.** Performed Activation Likelihood Estimation Meta-Analyses^a^

Patient or experimental group	Analysis	Experiments, No.	Significant clusters
Sleep disorders	All	95	Bilateral subgenual anterior cingulate cortex, right amygdala and hippocampus
	Decrease	76	Bilateral subgenual anterior cingulate cortex
	Increase	69	Right amygdala and hippocampus
	rs-fMRI	38	Left insula and putamen
	t-fMRI	19	Not significant
	Tasks	20	Not significant
	Functional	66	Left insula and putamen
	sMRI	45	Left thalamus
	Local	91	Right amygdala and hippocampus, bilateral subgenual anterior cingulate cortex
	Adults	86	Bilateral subgenual anterior cingulate cortex
	Higher power	51	Bilateral subgenual anterior cingulate cortex
	Corrected	77	Not significant
Sleep deprivation	All	45	Right thalamus
	Decrease	33	Not significant
	Increase	35	Right thalamus
	t-fMRI	33	Right insula and frontal operculum
	Tasks	36	Right insula and frontal operculum
	Functional	45	Right thalamus
	Local	45	Right thalamus, right insula and frontal operculum
	Adults	42	Right thalamus, right insula and frontal operculum
	Higher power	24	Right thalamus
	Corrected	33	Right thalamus
Insomnia disorder	All	28	Bilateral subgenual anterior cingulate cortex
	Decrease	20	Right inferior and middle frontal gyrus
	Increase	24	Not significant
	Functional	25	Not significant
	Local	24	Not significant
	Adults	28	Bilateral subgenual anterior cingulate cortex
	Higher power	19	Not significant
	Corrected	23	Not significant
Obstructive sleep apnea	All	28	Not significant
	Decrease	25	Not significant
	Increase	18	Not significant
	Functional	17	Not significant
	Local	27	Not significant
	Adults	25	Not significant
	Higher power	19	Not significant
	Corrected	23	Not significant
Total sleep deprivation	All	38	Right thalamus
	Decrease	30	Not significant
	Increase	29	Not significant
	t-fMRI	27	Right insula and frontal operculum
	Tasks	30	Right insula and frontal operculum
	Functional	38	Right thalamus
	Local	38	Not significant
	Adults	37	Not significant
	Higher power	20	Right thalamus
	Corrected	29	Right thalamus
	22-26 h	20	Not significant

Abbreviations: rs-fMRI, resting-state functional magnetic resonance imaging; sMRI, structural magnetic resonance imaging; t-fMRI, task-based functional magnetic resonance imaging.

^a^ Decrease indicates analyses including any kind of experiment showing a decrease in activity, connectivity, metabolism, gray matter, or brain volume in the patient or experimental group; increase, analyses including any kind of experiment showing an increase in activity, connectivity, metabolism, gray matter, or brain volume in the patient or experimental group; local, analyses including only experiments of local voxelwise measures; corrected, analyses including only coordinates that are corrected for multiple comparisons; higher power, analyses including only experiments with a sample size of at least 21 participants, ie, higher power; 22-26 h, analyses including only experiments performed after approximately 22 to 26 hours of total sleep deprivation (time around the circadian nadir for alertness). Analyses were only performed when at least 17 experiments were included. The statistical significance threshold was set at *P* < .001 for the voxel level and *P* < .05 for cluster-level familywise error correction.

## Discussion

We observed unique brain alterations across sleep disorders (bilateral sgACC and right amygdala and hippocampus) and sleep deprivation (right thalamus). Interestingly, the identified convergent alterations were mostly located on the right side of the brain, suggesting an association between sleep disturbance conditions and brain asymmetry.^43,44^ Previous CBMAs also observed right-side OSA-associated alterations in the amygdala and hippocampus and the insula^23^ and sleep deprivation–associated alterations in the parietal cortex.^26^ Total sleep deprivation has been shown to impair lateralized spatial working memory.^44^

### Convergent Abnormality Across Sleep Disorders

The transdiagnostic analysis identified abnormalities in the sgACC and right amygdala and hippocampus. The sgACC was associated with reward, reasoning, and gustation, and the right amygdala and hippocampus cluster was associated with negative emotion processing, memory, and olfaction. Both clusters were connected to the respective contralateral areas and the DMN. The observed abnormalities could be secondary effects of common nocturnal symptoms (eg, abnormal timing, efficiency, alertness, duration, satisfaction, and regularity) and/or daytime symptoms (eg, sleepiness, fatigue, cognitive impairment, emotion dysregulation, and affective symptoms) across sleep disorders.^6,18,45^ Of note, our transdiagnostic CBMA aimed to identify potential regional convergence across sleep disorders,^46,47,48^ without dismissing their distinct etiologies and mechanisms.

Insomnia disorder, OSA, narcolepsy, RLS, congenital central (alveolar) hypoventilation syndrome, and RBD experiments formed these 2 clusters. These transdiagnostic patterns align with the diagnosis-specific abnormalities, such as the sgACC in insomnia disorder (51 articles), supported by our recent CBMA (39 articles).^22^ While OSA showed inconsistent patterns (49 articles), a previous multimodal CBMA across 16 articles on OSA observed the right amygdala and hippocampus and the right insula.^23^ This discrepancy could be attributable to the statistical power of these CBMAs, heterogeneity of included clinical populations, and variability in the experimental and statistical choices of included studies. Narcolepsy revealed no consistent regional abnormalities previously (15 articles),^21^ which needs further replication in the future. To our knowledge, there is no existing CBMA in other sleep disorders due to limited existing data.

Sleep modifies the synchronous activity of the amygdala-hippocampal-cortical circuit, which is essential for adaptive emotional processes and memory.^49^ Structural and functional abnormalities of the right amygdala and hippocampus have been reported in insomnia disorder,^50,51,52^ OSA,^25,53^ RLS and periodic limb movement,^54^ and narcolepsy.^55^ Altered gray matter volume, structural and functional dysconnectivity of the sgACC, and its abnormal neurotransmitter distribution in insomnia disorder,^56,57,58,59^ OSA,^60^ and RLS have been reported as well.^61^ The key role of the right amygdala and hippocampus and the sgACC in the interplay between sleep disorders and their most common comorbidities, such as anxiety and depression, has been well documented,^19,50,62,63^ which could be associated with negative emotion processing, rumination, and memory impairments that are common in patients with comorbid sleep and affective disorders.^64^ Our behavioral decoding analysis supports this evidence. Our connectivity analysis of the right amygdala and hippocampus and the sgACC pointed to the DMN, where abnormalities were observed across a wide range of sleep disorders.^18,65,66,67^

### Convergent Alteration Across Total or Partial Sleep Deprivation

The ALE analysis across sleep deprivation experiments revealed a significant cluster in the right thalamus, associated with thermoregulation, action, and pain perception and showing distinct connectivity with subcortical and (pre)motor regions. Previous CBMAs in sleep deprivation highlighted dysfunction of the right thalamus and parietal lobule.^26,28^ Sleep deprivation may lead to the synaptic molecular machinery in those regions.^68^ Altered right thalamus activity^69,70^ and disrupted thalamic-cortical connectivity^71,72,73^ following sleep deprivation have been associated with attention deficits and increased daytime sleepiness. Disrupted connectivity between the somatosensory thalamic area and the right middle temporal gyrus was associated with attention deficits after sleep deprivation. Our connectivity results align with the disrupted FC between the right thalamus and parietal, somatosensory, premotor, and subcortical regions due to sleep deprivation.^72^ Thus, the thalamic function appears to be greatly affected by sleep deprivation.

### Strengths and Limitations

We followed the best-practice guidelines^29,30^ by preregistering the study, searching various databases, merging overlapping samples, excluding non–whole-brain studies, using strict correction methods for multiple comparisons for robust findings, and providing the data and analyses for transparency. However, the study had several limitations. Our findings should be interpreted cautiously as the included studies do not represent the entire neuroimaging literature. Numerous studies were excluded because they performed (hidden) region-of-interest analyses. Moreover, the percentage of included experiments varied substantially among different disorders, sleep deprivation, and imaging modalities, which could affect the results (eg, insomnia disorder and OSA were overrepresented compared with other disorders). However, as the pathophysiology of sleep disorders is different in children vs adults (eg, OSA) and the impact of pharmacological and nonpharmacological treatment of sleep disorders on the brain is inevitable,^74,75^ the effects of these factors should be explored in future CBMAs. Additionally, the sleep deprivation ALE analyses included experiments with varying wake durations and circadian phases, potentially introducing confounding factors. A subanalysis focusing solely on approximately 24-hour experiments yielded nonsignificant results. However, the right thalamus cluster was consistently replicated across various subanalyses, including those limited to total sleep deprivation, and remained robust even with the addition of noise experiments, unlike the sleep disorder clusters. Lastly, the neurobiological influence of similar symptoms across sleep disorders should be further explored in the future. Interventions could target these regions to reduce the burden and symptoms of sleep disorders.

## Conclusions

This comprehensive CBMA demonstrated distinct neurobiological substrates for long-term sleep disorders and short-term sleep deprivation. Despite various etiologies, we identified consistent abnormalities in the sgACC and the right amygdala and hippocampus across sleep disorders, probably due to similar nocturnal and daytime symptoms. Those regions were associated with emotional and cognitive processes and were connected to the DMN. The right thalamus was consistently identified in short-term sleep deprivation and was associated with distinct behavioral domains and connectivity patterns.

## Notes

Supplement 1.
eMethods

eResults
**eTable 1.** Exclusion Criteria **eTable 2.** Included Sleep Disorder Experiments **eTable 3.** Included Sleep Deprivation Experiments **eTable 4.** Number of Experiments for Each Meta-Analysis **eTable 5.** Convergent Regional Alterations Based on Patient/Experimental Group **eTable 6.** Convergent Regional Alterations Based on “Control > Patient/Experimental” (Decrease) **eTable 7.** Convergent Regional Alterations Based on “Patient/Experimental > Control” (Increase) **eTable 8.** Convergent Regional Alterations Based on rs-fMRI **eTable 9.** Convergent Regional Alterations Based on t-fMRI **eTable 10.** Convergent Regional Alterations Based on Tasks **eTable 11.** Convergent Regional Alterations Based on Functional **eTable 12.** Convergent Regional Alterations Based on sMRI **eTable 13.** Convergent Regional Alterations Based on Local Measures **eTable 14.** Convergent Regional Alterations Based on Adults-Only **eTable 15.** Convergent Regional Alterations Based on Higher Power Experiments **eTable 16.** Convergent Regional Alterations Based on Corrected Coordinates **eFigure 1.** Significant Behavioural Decoding Domain Subcategories for the Meta-Analysis Across All Sleep Disorders **eFigure 2.** Task-Based and Task-Free Connectivity for the Bilateral Subgenual Anterior Cingulate Cortex Cluster **eFigure 3.** Task-Based and Task-Free Connectivity for the Right Amygdala/Hippocampus Cluster **eFigure 4.** Behavioural Decoding of Clusters From Sleep Deprivation Meta-Analysis **eFigure 5.** Task-Based and Task-Free Connectivity for the Right Thalamus Cluster **eFigure 6.** Shared Connectivity Maps of Clusters From Meta-Analyses Across Sleep Disorders and Sleep Deprivation **eFigure 7.** Convergent Regional Alterations Based on Patient/Experimental Group **eFigure 8.** Convergent Regional Alterations Based on “Control > Patient/Experimental” (Decrease) **eFigure 9.** Convergent Regional Alterations Based on “Patient/Experimental > Control” (Increase) **eFigure 10.** Convergent Regional Alterations Based on rs-fMRI **eFigure 11.** Convergent Regional Alterations Based on t-fMRI **eFigure 12.** Convergent Regional Alterations Based on Tasks **eFigure 13.** Convergent Regional Alterations Based on Functional **eFigure 14.** Convergent Regional Alterations Based on sMRI **eFigure 15.** Convergent Regional Alterations Based on Local Measures **eFigure 16.** Convergent Regional Alterations Based on Adults-Only **eFigure 17.** Convergent Regional Alterations Based on Higher Power Experiments **eFigure 18.** Convergent Regional Alterations Based on Corrected Coordinates
eReferences

Supplement 2. Data Sharing Statement
