# Supplementary material for: Distinct Convergent Brain Alterations in Sleep Disorders and Sleep Deprivation: A Meta-Analysis
Source: JAMA Psychiatry. 2025 Apr 23;82(7):681–91. doi: 10.1001/jamapsychiatry.2025.0488 (PMC12019678; doi:10.1001/jamapsychiatry.2025.0488)
Supplement: Supplement 1. — eMethods eResults eTable 1. Exclusion Criteria eTable 2. Included Sleep Disorder Experiments eTable 3. Included Sleep Deprivation Experiments eTable 4. Number of Experiments for Each Meta-Analysis eTable 5. Convergent Regional Alterations Based on Patient/Experimental Group eTable 6. Convergent Regional Alterations Based on “Control > Patient/Experimental” (Decrease) eTable 7. Convergent Regional Alterations Based on “Patient/Experimental > Control” (Increase) eTable 8. Convergent Regional Alterations Based on rs-fMRI eTable 9. Convergent Regional Alterations Based on t-fMRI eTable 10. Convergent Regional Alterations Based on Tasks eTable 11. Convergent Regional Alterations Based on Functional eTable 12. Convergent Regional Alterations Based on sMRI eTable 13. Convergent Regional Alterations Based on Local Measures eTable 14. Convergent Regional Alterations Based on Adults-Only eTable 15. Convergent Regional Alterations Based on Higher Power Experiments eTable 16. Convergent Regional Alterations Based on Corrected Coordinates eFigure 1. Significant Behavioural Decoding Domain Subcategories for the Meta-Analysis Across All Sleep Disorders eFigure 2. Task-Based and Task-Free Connectivity for the Bilateral Subgenual Anterior Cingulate Cortex Cluster eFigure 3. Task-Based and Task-Free Connectivity for the Right Amygdala/Hippocampus Cluster eFigure 4. Behavioural Decoding of Clusters From Sleep Deprivation Meta-Analysis eFigure 5. Task-Based and Task-Free Connectivity for the Right Thalamus Cluster eFigure 6. Shared Connectivity Maps of Clusters From Meta-Analyses Across Sleep Disorders and Sleep Deprivation eFigure 7. Convergent Regional Alterations Based on Patient/Experimental Group eFigure 8. Convergent Regional Alterations Based on “Control > Patient/Experimental” (Decrease) eFigure 9. Convergent Regional Alterations Based on “Patient/Experimental > Control” (Increase) eFigure 10. Convergent Regional Alterations Based on rs-fMRI eFigure 11. Convergent Regional Alte [file jamapsychiatry-e250488-s001.pdf]

Supplemental Online Content

Reimann GM, Hoseini A, Koçak M, et al. Distinct convergent brain alterations in sleep disorders and sleep deprivation: a meta-analysis . *JAMA Psychiatry*. Published online April 23, 2025.
doi:10.1001/jamapsychiatry.2025.0488

eMethods
eResults
eTable 1. Exclusion Criteria
eTable 2. Included Sleep Disorder Experiments
eTable 3. Included Sleep Deprivation Experiments
eTable 4. Number of Experiments for Each Meta-Analysis
eTable 5. Convergent Regional Alterations Based on Patient/Experimental Group
eTable 6. Convergent Regional Alterations Based on “Control > Patient/Experimental” (Decrease)
eTable 7. Convergent Regional Alterations Based on “Patient/Experimental > Control” (Increase)
eTable 8. Convergent Regional Alterations Based on rs-fMRI
eTable 9. Convergent Regional Alterations Based on t-fMRI
eTable 10. Convergent Regional Alterations Based on Tasks
eTable 11. Convergent Regional Alterations Based on Functional
eTable 12. Convergent Regional Alterations Based on sMRI
eTable 13. Convergent Regional Alterations Based on Local Measures
eTable 14. Convergent Regional Alterations Based on Adults-Only
eTable 15. Convergent Regional Alterations Based on Higher Power Experiments
eTable 16. Convergent Regional Alterations Based on Corrected Coordinates
eFigure 1. Significant Behavioural Decoding Domain Subcategories for the Meta-Analysis Across All Sleep Disorders
eFigure 2. Task-Based and Task-Free Connectivity for the Bilateral Subgenual Anterior Cingulate Cortex Cluster
eFigure 3. Task-Based and Task-Free Connectivity for the Right Amygdala/Hippocampus Cluster
eFigure 4. Behavioural Decoding of Clusters From Sleep Deprivation Meta-Analysis
eFigure 5. Task-Based and Task-Free Connectivity for the Right Thalamus Cluster
eFigure 6. Shared Connectivity Maps of Clusters From Meta-Analyses Across Sleep Disorders and Sleep Deprivation
eFigure 7. Convergent Regional Alterations Based on Patient/Experimental Group
eFigure 8. Convergent Regional Alterations Based on “Control > Patient/Experimental” (Decrease)
eFigure 9. Convergent Regional Alterations Based on “Patient/Experimental > Control” (Increase)
eFigure 10. Convergent Regional Alterations Based on rs-fMRI
eFigure 11. Convergent Regional Alterations Based on t-fMRI
eFigure 12. Convergent Regional Alterations Based on Tasks
eFigure 13. Convergent Regional Alterations Based on Functional
eFigure 14. Convergent Regional Alterations Based on sMRI
eFigure 15. Convergent Regional Alterations Based on Local Measures
eFigure 16. Convergent Regional Alterations Based on Adults-Only
eFigure 17. Convergent Regional Alterations Based on Higher Power Experiments
eFigure 18. Convergent Regional Alterations Based on Corrected Coordinates
eReferences

This supplemental material has been provided by the authors to give readers additional information about their work.

© 2025 Reimann GM et al. *JAMA Psychiatry*.

eMethods

Search terms

We searched PubMed, Web of Science, Embase, and Scopus with following search items slightly adjusted to fit each database: (sleep* OR somno* OR hypno* OR hypersomnia OR dyssomnia OR parasomnia OR insomnia OR narcolepsy OR "night terror" OR "periodic limb movements" OR "restless leg syndrome" OR nightmare OR OSA OR SDB OR RBD OR PLM OR RLS) AND (fMRI OR "Functional MRI" OR "functional magnetic resonance imaging" OR PET OR "positron emission tomography" OR "voxel-based morphometry" OR VBM). For database-specific prompts and BrainMap search strategy see https://osf.io/2w5v9/

Literature Search and Article Selection

A thorough literature search was carried out across PubMed, Web of Science, Embase, Scopus, and BrainMap, focusing on neuroimaging studies related to sleep disorders and deprivation up to January 2024. Additional studies were identified through reference tracing of relevant reviews and meta-analyses.

Initially, an in-house R script was employed to automatically remove duplicated reports and exclude abstracts based on metadata criteria (e.g., excluding non-English articles) and article type (e.g., excluding grey literature). This was followed by an initial screening of the titles and abstracts and, subsequently, a full-text screening based on our selection criteria. At least two raters independently screened each article (AH, MK, MB, VK). In case of disagreement between them, additional authors (GR and MT) were consulted. If reports were unclear or lacked coordinates, we contacted the authors to obtain the necessary details. Of note, reports that were already included in one of our previous meta-analyses[1-4] were isolated and double-checked by two raters (AH, GR), including their already extracted data, which we incorporated into our database.

We collected the following data from each included study: i) sample characteristics: this included details such as population type, sample size, female-to-male ratio, mean age, age range, and diagnostic information or extent of sleep deprivation; ii) methodological details: information about imaging modality, specific measures or tasks used, and, for sleep deprivation studies, the study design (e.g., before-after or case-control); iii) additional characteristics: number of foci reported, brain space utilized, statistical thresholds (e.g., p-values), and covariates included in the analyses; iv) peak coordinates: extracted as (X, Y, Z) values. The data extraction was initially performed by one author and subsequently independently reviewed and verified by a second author (GR and AH).

Experiment Merging

To account for sample size variations affecting probability distributions in the ALE analysis, we selected the smaller group size (either "patient/experimental" or "control") to represent each experiment's sample size, following previous recommendations.[5] For merged experiments, group-wise sample sizes were calculated independently before selection: the mean was used when two experiments were merged, and the median was applied when merging more than two experiments. In these cases, the smaller group-wise sample size was used as the representative sample size to ensure consistency in the ALE analysis.

Activation Likelihood Estimation Meta-Analysis

In this study, all meta-analyses were performed according to standard analysis procedures[6,7] using the revised version of activation likelihood estimation (ALE).[5] ALE was chosen for its ability to handle spatial uncertainty, being the most used coordinate-based meta-analysis (CBMA) method,[7] and its robust correction for multiple comparisons to minimize spurious findings.[5] The ALE analysis involved three main steps. Initially, coordinates reported from individual neuroimaging articles were pooled and mapped into a three-dimensional brain space. If any article reported Talairach coordinates, they were first transformed into MNI space to have all coordinates in the same reference space. The coordinates were then modelled as centres of three-dimensional Gaussian probability distributions to account for spatial uncertainty. The widths of these distributions were determined by empirical estimates of variations between subjects, different imaging techniques, templates, and normalization methods, with the between-subject variation of each focus being based on the sample size of its corresponding experiment (i.e., larger sample sizes resulting in more localized distributions). Next, all probability distributions in each experiment were aggregated into "modelled activation" (MA) maps so that for every voxel, only the highest probability from any focus within that experiment was considered. This method prevents the cumulative influence of multiple nearby foci within a single experiment on the probability values. By unifying the MA maps from all experiments into the ALE map, voxel-wise ALE scores were generated. These scores indicate the convergence of results at specific brain locations. Finally, the ALE map was compared to a null distribution, which represented random spatial association, using nonlinear histogram integration to determine probabilities that exceeded chance levels. To correct for multiple tests, the sizes of observed clusters were compared to a null distribution of cluster sizes derived from a permutation approach with 10,000 repetitions.[5] The significance threshold was set at $p < 0.05$ at the cluster-level family-wise error (cFWE) with a cluster-forming threshold of $p < 0.001$ at the voxel level.[6-8] Additionally, we used Bonferroni correction to correct for multiple comparisons across the performed sub-ALEs due to their high number.

Activation Likelihood Estimation Contrast and Conjunction Analyses

Contrast and conjunction meta-analyses can be used to compare two ALE results (e.g., sleep disorders vs. sleep deprivation) in order to identify regions of the brain that are uniquely or jointly activated across the conditions. In the contrast analysis, the difference between the ALE maps is calculated and then compared to the null distribution, with family-wise error (FWE) correction applied at $p < 0.05$. In the conjunction analysis, the cFWE-corrected Z-score volumes from the standard analyses are combined using a minimum conjunction approach to identify overlapping regions of activation.

© 2025 Reimann GM et al. *JAMA Psychiatry*.

Activation Likelihood Estimation Based on Local Measures

This type of sub-analysis included only local voxel-wise measures (such as structural magnetic resonance imaging (sMRI), task-based functional MRI (t-fMRI), and voxel-based physiology). Voxel-based physiology encompasses measures such as regional cerebral blood flow, regional homogeneity (ReHo), amplitude of low-frequency fluctuations (ALFF), dynamic ALFF, fractional ALFF, dynamic fractional ALFF, intrinsic connectivity contrast (ICC), brain entropy (BEN), short-range/local functional connectivity density (srFCD), and regional glucose metabolism from PET and fMRI, which have shown co-localized alterations with VBM in psychiatric disorders.[9] Therefore, global functional connectivity measures - including functional stability (FS), long-range FCD (lrFCD), global FCD (gFCD), functional connectivity strength (FCS), graph theory, independent component analysis (ICA), and voxel-mirrored homotopic connectivity (VMHC) - were excluded from the local measures analyses.

Contribution Analysis

We calculated the relative contribution of each experiment to each significant cluster by first dividing the ALE map without the respective experiment by the ALE map including all experiments of an analysis. This was then averaged across all voxels in each cluster to obtain the average ALE contribution for each experiment. Finally, we normalized these individual contributions by dividing them by the total average ALE contribution, scaling them to sum to 100% for comparison.

Fail-Safe N

Fail-Safe N (FSN)[10] analysis was utilized to assess the robustness of identified clusters and to address potential publication bias. FSN determines the maximum number of noise experiments that can be added to the dataset while retaining statistical significance for the cluster of interest. Noise experiments were generated and integrated into the primary analysis datasets using a Python-based in-house implementation. Consistent with recommendations from the original publication, the lower boundary for the number of noise experiments was set at 30% of the included experiments, while the upper boundary was defined to ensure that the original contributing experiments constituted at least 10% of the total dataset. FSN values were computed within these boundaries. If the FSN falls below the lower boundary, this indicates that the cluster may lack robustness. Conversely, if the FSN exceeds the upper boundary, it suggests that the observed results might be disproportionately influenced by a small subset of studies.

Behavioural Decoding

Behavioural decoding analysis was used to functionally examine the found ALE clusters. We used the BrainMap database,[11] which comprises over 15,000 functional and structural neuroimaging experiments to date. This database categorizes behaviour into five main domains (i.e., action, cognition, emotion, interoception, and perception), with several sub-domains.[12] First, we searched for task-based experiments that included our clusters, excluding those involving pharmacological interventions or between-group comparisons, to ensure the focus remained on brain areas under normal physiological conditions. Then, we tested whether the conditional probability of activation for specific sub-domains, P(Activation | Domain), was higher than the overall baseline probability of activation across all categories. Lastly, a binomial test was employed and corrected for a false discovery rate (FDR, $p < 0.05$).

Task-based Functional Connectivity - Meta-analytic Connectivity Modeling

To assess task-dependent functional connectivity (FC), we performed meta-analytic connectivity modeling (MACM)[12,13] on 7,937 task-based fMRI experiments in healthy adults in the BrainMap database. MACM identifies coactivated regions by conducting a meta-analysis across all foci from experiments that activate the cluster.[12,13] We applied a significance threshold of $p < 0.05$, cFWE-corrected.

Task-free Functional Connectivity - Resting-state Functional Connectivity

We conducted a voxel-wise seed-based resting-state FC (rsFC) analysis to determine task-free FC patterns of identified clusters across sleep disorders and sleep deprivation experiments. The rs-fMRI data came from the enhanced Nathan Kline Institute - Rockland sample (eNKI-RS).[14] This sample included 192 healthy adults (65.1% female, age 20–75, mean age 46.4 ± 16.7 years) who underwent a 10-minute rs-fMRI scan using a Siemens TimTrio 3 T scanner. The local ethics committee of Heinrich Heine University Hospital of Düsseldorf approved re-analyzing these pre-existing data. Images were pre-processed with SPM 12 and in-house MATLAB (version 9.4 (R2018a). Natick, Massachusetts: The MathWorks Inc.; 2018) scripts, including removing the first four scans, performing FIX (FMRIB's ICA-based Xnoiseifier) together with correcting for head motion, normalizing to MNI space, and spatial smoothing with a 5 mm Gaussian kernel. Subsequently, the cluster's time course was extracted, white matter and cerebrospinal fluid signals were removed, and then we applied a band-pass filter (0.01 to 0.08 Hz). Pearson's correlation between the cluster's time course and all other voxels' time series was calculated, creating rsFC maps. These maps were transformed into Fisher’s Z-scores and entered into a second-level ANCOVA for group analysis, considering age and sex as covariates. Results were corrected for multiple comparisons, with significance set at $p < 0.05$, cFWE-corrected.

© 2025 Reimann GM et al. *JAMA Psychiatry*.

eResults

Exclusion of Articles Included in Previous Meta-Analyses

Before this work, we already have performed multimodal meta-analyses on insomnia disorder,[15] narcolepsy,[4] OSA,[16] and sleep deprivation.[3] Some of these meta-analyses were conducted nearly a decade ago. Since then, new guidelines[6,7] have emerged, and with updated inclusion and exclusion criteria, experiments previously included may no longer be eligible. Therefore, we re-screened all previously included articles, excluding any that did not fulfil our criteria (eTable 1). One insomnia disorder article[17] was excluded based on our "no patient/experimental vs. control contrast" criterium. We excluded six narcolepsy articles, one for having no patient/experimental vs. control contrast,[18] one for having no peer-reviewed final results,[19] one using a non-eligible method,[20] and the remaining three for being region-of-interest analyses.[21-23] Of the sleep deprivation articles, nine were excluded; two had no patient/experimental vs. control contrast[24,25] and seven turned out to be region-of-interest analyses.[26-32] None of the OSA articles needed to be excluded.

Convergent Regional Alterations Based on Patient/Experimental Group

When focusing on specific patient/experimental groups, there were only enough insomnia disorder, OSA, and total sleep deprivation experiments (≥17) to perform an ALE analysis (eTable 4). For insomnia disorder, the analysis revealed significant convergence in the bilateral subgenual anterior cingulate cortex (sgACC) (eTable 5, eFigure 7A). In the case of total sleep deprivation, the analysis identified a cluster in the right thalamus (rThal). Both were also observed in their respective main analysis (eTable 5, eFigure 7B). The OSA analysis was not significant.

Convergent Regional Alterations Based on „Control > Patient/Experimental" (Decrease)

When including only foci of "Control > Patient/Experimental" contrasts, indicating a decrease in activity, connectivity, or grey matter in the patient/experimental group, there were enough experiments (≥17) for the analyses across all sleep disorders, sleep deprivation, insomnia disorder, OSA, and total sleep deprivation (eTable 4). Clusters were identified in the analyses across all sleep disorders and insomnia disorder (eTable 6), with the first identifying the sgACC cluster (eFigure 8A). In the analysis of insomnia disorder, a new cluster emerged in the right inferior frontal gyrus, pars opercularis, and middle frontal gyrus (rIFG/rMFG) (eFigure 8B).

Convergent Regional Alterations Based on „Patient/Experimental > Control" (Increase)

When focusing only on foci of "Patient/Experimental > Control" contrasts (i.e. increased activity, connectivity, or grey matter in the patient/experimental group), there were enough experiments to conduct analyses for all sleep disorders, sleep deprivation, insomnia disorder, OSA, and total sleep deprivation (eTable 4). We found significant clusters in the analyses of all sleep disorders, and sleep deprivation/restriction (eTable 7), which replicated most findings of the main text analyses. Specifically, the comprehensive analysis across all sleep disorders highlighted the rAmyg/rHipp cluster (eFigure 8A), while the sleep deprivation analysis confirmed the rThal cluster (eFigure 9B).

Convergent Regional Alterations Based on rs-fMRI

We performed ALE meta-analyses focusing solely on rs-fMRI experiments across all sleep disorders (eTable 4), which yielded a significant cluster located in the left insula cortex and left putamen (lIns/lPut) (eTable 8, eFigure 10).

Convergent Regional Alterations Based on t-fMRI

Another modality-specific analysis was based exclusively on t-fMRI experiments. There were sufficient experiments for analyses across all sleep disorders, sleep deprivation, and total sleep deprivation (eTable 4). A significant cluster was identified in both deprivation analyses but not across sleep disorders located in the rIns/rFO (eTable 9, eFigure 11), which was also observed in the main text analysis.

Convergent Regional Alterations Based on PET

There were not enough experiments to conduct a valid analysis for PET-only (eTable 4).

Convergent Regional Alterations Based on Tasks

A modality-specific analysis was conducted using only task-based experiments, including t-fMRI and PET studies that involved task performance. This approach enabled analyses for all sleep disorders, sleep deprivation, and total sleep deprivation (eTable 4). Significant clusters were identified in both sleep deprivation analyses, with a convergent alteration observed in the rIns/rFO (eTable 10, eFigure 12), but no significant cluster emerged from the analysis across all sleep disorders.

Convergent Regional Alterations Based on Functional Patterns

An analysis focused exclusively on functional imaging studies, incorporating rs-fMRI, t-fMRI, and PET experiments. This analysis was feasible for all sleep disorders, sleep deprivation, insomnia disorder, OSA, and total sleep deprivation (eTable 4). Across all sleep disorders, a significant cluster was identified in the lIns/lPut. Similarly, both sleep deprivation analyses revealed a significant cluster in the rThal, aligning with the findings reported in the main text (eTable 11, eFigure 13).

Convergent Regional Alterations Based on sMRI

sMRI-specific (voxel-based morphometry (VBM), deformation-based morphometry (DBM)) ALE meta-analyses were conducted across all sleep disorders (eTable 4), revealing a new cluster in the left thalamus (lThal) (eTable 12, eFigure 14).

© 2025 Reimann GM et al. *JAMA Psychiatry*.

Convergent Regional Alterations Based on Local Measures

Sub-analyses were performed, including only "local" measures. There were enough experiments for meaningful analyses across all sleep disorders, sleep deprivation, insomnia disorder, OSA, and total sleep deprivation (eTable 4). The analyses across all sleep disorders and sleep deprivation each showed significant convergence (eTable 13). The analysis across all sleep disorders replicated the rAmyg/rHipp cluster, as well as the bilateral sgACC found in the main text analysis (eFigure 15A). The sleep deprivation analysis again identified the same cluster as in the main text analysis in the rThal and another in the rIns/rFO (eFigure 15B).

Convergent Regional Alterations Based on Adults-only

Sub-analyses were conducted, focusing exclusively on adults (≥ 18 years). Sufficient experiments were available for meaningful comparisons across all sleep disorders, sleep deprivation, insomnia disorder, OSA, and total sleep deprivation (eTable 4). Significant convergence was observed in the analyses for all sleep disorders, sleep deprivation, and insomnia disorder (eTable 14). Specifically, the analysis of all sleep disorders replicated the bilateral sgACC cluster identified in the main analysis (eFigure 16A). Similarly, the sleep deprivation analysis confirmed the same cluster in the rThal as observed in the main analysis as well as a rIns/rFO cluster (eFigure 16B). Finally, the insomnia disorder analysis also highlighted the sgACC cluster (eFigure 16C).

Convergent Regional Alterations Based on Higher Power Experiments

We conducted sub-analyses on experiments with a minimum sample size of 21 participants, indicating sub-sample of enhanced statistical power. This approach provided sufficient data for sleep deprivation, insomnia disorder, OSA, and total sleep deprivation (eTable 4). The clusters identified in the main analyses were replicated (eTable 15), notably the sgACC across all sleep disorders (eFigure 17a) and the rThal in sleep deprivation (eFigure 17b) and total sleep deprivation (eFigure 17c).

Convergent Regional Alterations Based on Corrected Coordinates

We performed sub-analyses including only coordinates that were corrected for multiple comparisons across all conditions with enough experiments (i.e., sleep disorders, sleep deprivation, insomnia disorder, OSA, and total sleep deprivation) (eTable 4). Only the analyses of sleep deprivation and total sleep deprivation yielded a cluster (eTable 16) in the rThal (eFigure 18).

Convergent Regional Alterations Based on 22h-26h

As the human circadian nadir for alertness lies around 24h, we also conducted a new analysis of total sleep disorder studies whose participants were sleep-deprived for around 22h-26h (eTable 4). This analysis based on 20 experiments was not significant.

Fail-Safe N of Meta-analyses across Sleep Disorders and across Sleep Deprivation

The analysis across sleep disorders included 95 experiments, with 17 contributing to the sgACC cluster and 25 to the rAmyg/rHipp cluster. Accordingly, the lower boundary was set at n = 28 for both clusters, while the upper boundary was set at n = 255 for the sgACC and n = 153 for the rAmyg/rHipp. When applying the lower boundary, both clusters were no longer statistically significant. For the rThal cluster in the sleep deprivation analysis, derived from 45 experiments with 13 contributing to the cluster, the boundaries were set at n = 13 (based on 45 experiments) and n = 117 (based on 13 contributing experiments). The calculated FSN for the rThal cluster was 43 noise experiments.

eTable 1. Exclusion Criteria

Step	Exclusion Criterion	Further Details/Reason
**Abstract screening**	Animal research	We are only interested in human subjects.
	Case report	Only a single or few subjects, hence no generalizable inference and statistics that can be used.
	Different language	In this study, only English is included for transparency.
	No peer-reviewed final results	This includes:<br>1. Grey literature such as conference abstracts, preprints and preceding articles, editorial letters, as well as theses, as they are not peer-reviewed and may only report preliminary results that may change in future<br>2. Books, errata, and protocols, which usually lack results.
	No sleep disorder/ deprivation	Articles that do not fulfil the criteria for a clinical disorder based on established diagnostic criteria such as international classification of sleep disorders, second/third edition (ICSD-2/3) or diagnostic and statistical manual of mental disorders, fourth/fifth edition (DSM-IV/V), and/or do not perform a total/partial sleep deprivation experiment.
	Review or meta-analysis	Both reviews and meta-analyses do not include "novel" data.
	Sleep disorder with comorbidity/deprivation with disease	Articles that deal with sleep disorders/deprivation, but subjects are also diagnosed with a comorbid disease such as neurological disorders like epilepsy, migraine, Parkinson's disease, Alzheimer's disease, and multiple sclerosis, or psychiatric disorders like major depression, bipolar disorder, schizophrenia, anxiety, and autism.<br><br>Articles that perform a sleep deprivation paradigm on sleep disorder patients.<br><br>Only in case of rapid eye movement sleep behaviour disorder, articles with Parkinson's disease are included due to the strong comorbidity of both disorders and the indistinct transition.
**Full text screening**	No patient or experimental vs. control contrast	This includes:<br>1. Articles that do not include a clear condition vs. control contrast (e.g., "patient type 1 vs. patient type 2 vs. control").<br>2. Longitudinal articles or articles that perform interventions, if a baseline vs. baseline contrast does not exist.<br>3. Interactions (e.g., state x group, state/group x trial).<br>4. Articles that indirectly compare groups (e.g., exclusive masks such as “activated in A but not in B”).<br><br>In case of sleep deprivation:<br>5. Articles that have no baseline (i.e., no sleep deprivation) contrast (e.g., 24h vs. 48h of sleep deprivation).<br>6. Articles that scan sleep deprived participants after a recovery night instead directly after the total/partial deprivation.
	No reported coordinates	No coordinates in text, images, or tables of the full text, or its supplementary files, and when asking the authors.
	No reported difference	If the analysis led to no significant results.
	No standard space	Coordinates reported in a brain space that is not Montreal neurological institute (MNI, MNI305, ICBM152, etc.) or Talairach space.

© 2025 Reimann GM et al. *JAMA Psychiatry*.

Step	Exclusion Criterion	Further Details/Reason
**Full text screening**	Ineligible method	This includes:<br>1. Articles that do not perform any neuroimaging analysis.<br>2. Articles that perform neuroimaging analyses that do not result in brain coordinates (e.g., electroencephalography, most machine learning techniques, global graph theory measures).<br>3. White matter and perfusion-focused methods.<br>4. Single-photon emission computed tomography
	Region of interest	Region-of-interest analyses lead to inflated significance in the a priori defined regions and violate the assumption in activation likelihood estimation that every voxel is able to show convergence<br><br>This includes:<br>1. Region-of-interest analyses, such as seed-based functional connectivity or hidden region-of-interest analysis (e.g., volume size smaller than brain size [140 mm x 167 mm x 93 mm]).<br>2. Small-volume correction.<br>3. Spectroscopy.<br>4. Diffusion tensor imaging.
	Sample size <7	Any of the groups (patient/experimental or control) has less than seven participants.

Abbreviations: DSM-IV/V: diagnostic and statistical manual of mental disorders, fourth/fifth edition, ICSD-2/3: international classification of sleep disorders, second/third edition

© 2025 Reimann GM et al. *JAMA Psychiatry*.

eTable 2. Included Sleep Disorder Experiments

	Author, Year	Nr. of Subjects (Female)		Age, Mean ± SD (Range)		Imaging Modality	Brain Space	Nr. of Foci	Diagnostic Manual	P-value	Covariates
		Patients	Controls	Patients	Controls						
**Congenital Central (Alveolar) Hypoventilation Syndrome**											
1	Macey et al., 2004[33 a]	13 (6)	14 (7)	10.9±2.3 (8-15)	10.9±2.2 (8-15)	t-fMRI (forced expiratory loading / voluntary expiratory efforts)	MNI	17	American thoracic society criteria	p<0.1 FDR[c]	NA
	Macey et al., 2005a[34 a]	13 (6)	14 (7)	10.9±2.3 (8-15)	11.0±2.2 (8-15)	t-fMRI (forehead cold pressor)	MNI	22	American thoracic society criteria	p<0.01, 6 vox.[d]	NA
	Macey et al., 2005b[35 a]	14 (7)	14 (7)	11±2 (8-15)	11±2 (8-15)	t-fMRI (hypoxic challenge)	MNI	16	American thoracic society criteria	p<0.05 FDR[c]	NA
	Woo et al., 2005[36 a]	14 (7)	15 (7)	11±2 (8-15)	11±2 (8-15)	t-fMRI (hyperoxia challenge)	MNI	11	American thoracic society criteria	p<0.05 FDR[c]	NA
**Hypersomnia**											
1	Dauvilliers et al., 2017[37 a]	9 (7)	19 (3)	27 (20-60)	36 (20-60)	PET (resting glucose metabolism)	TAL	7	ICSD-3	p<0.001, 50 vox.[d], p<0.05, 50 vox.[c]	age
2	Trotti et al., 2021[38 a]	16 (14)	9 (6)	38.1±8.2 (NA)	32.2±15.5 (NA)	PET (resting glucose metabolism)	TAL	17	ICSD-3	p<0.005, 100 vox. cFWE[c]	NA
**Insomnia Disorder**											
1	Altena et al., 2008[39]	21 (17)	12 (9)	61±6.2 (adult)	60±8.2 (adult)	t-fMRI (category and letter fluency task)	MNI	12	Established qualitative and quantitative criteria	z>3.1[d]	NA
	Altena et al., 2010[40]	24 (17)	13 (9)	60.3±6.0 (52-74)	60.2±8.4 (50-76)	VBM (GMD)	MNI	3	DSM-IV	p<0.001, 25 vox.[d], p<0.05 FWE[c]	age, total GM
	Stoffers et al., 2014[41]	24 (17)	13 (9)	60.3±6.0 (52-74)	60.1±8.3 (50-76)	t-fMRI (Tower of London task)	MNI	1	DSM-IV-TR	z>3.1, p<0.05 cFWE[c]	NA

© 2025 Reimann GM et al. *JAMA Psychiatry*.

2	Baglioni et al., 2014[42]	22 (15)	38 (21)	40.7±12.6 (NA)	39.6±8.9 (NA)	t-fMRI (sleep-related pictures vs. neutral pictures)	MNI	1	Research diagnostic criteria	p<0.05, 100 vox.[d]	NA
3	Chen et al., 2014[43]	17 (17)	17 (17)	27.16±6.67 (18-40)	27.56±6.83 (18-40)	rs-fMRI (ICA)	MNI	2	DSM-IV-TR, ICSD-2	p<0.001, p<0.05 FWE[c]	NA
4	Chen Z et al., 2022[44 a]	30 (7)	34 (5)	39.5 (18-60)	35 (18-60)	rs-fMRI (gFCD)	MNI	1	DSM-V	p<0.005, p<0.05 GRF[c]	age, gender, education, head movement
	Feng et al., 2022[45 a]	31 (8)	30 (10)	44.81±11.67 (18-60)	42.27±9.91 (18-60)	rs-fMRI (ReHo)	MNI	3	DSM-V	p<0.05 FDR[c]	age, gender, education level, BMI, mean FD
	Li S et al., 2021[46]	101 (77)	63 (31)	42.76±13.69 (adult)	37.10±9.76 (adult)	VBM (GMV)	MNI	5	DSM-V	p<0.001, p<0.05 FWE[c]	age, gender, years of education, total TIV
	Li J et al., 2023[47 a]	93 (65)	78 (50)	43.21±15.18 [n=28], 45.05±12.74 [n=65] (NA)	41.07±13.12 [n=28], 42.10±12.27 [n=65] (NA)	VBM (GMV)	MNI	1	DSM-IV, ICSD-3	p<0.005, p<0.05 GRF[c]	age, years of education, TIV
5	Chen W et al., 2022[48 a]	30 (21)	27 (20)	51±13 (25-65)	49±12 (NA)	rs-fMRI (dfALFF)	MNI	6	DSM-V	p<0.001, p<0.05 GRF[c]	age, sex, education level, mean FD
6	Chou et al., 2021[49]	22 (15)	27 (21)	40.2±11.7 (22-57)	41.3±10.1 (21-62)	VBM (GMV)	MNI	3	DSM-V	p<0.005, p<0.05 FWE, 208 vox.[c]	age, sex, TIV, HADS
7	Dai et al., 2014[50]	24 (17)	24 (12)	54.8±9.8 (NA)	52.5±6.6 (NA)	rs-fMRI (ReHo)	MNI	3	ICSD-2	p<0.01, p<0.05, 1080 mm^3[c]	age, sex, years of education
	Dai et al., 2016[51]	42 (27)	42 (24)	49.21±10.96 (NA)	49.14±10.2 (NA)	rs-fMRI (ALFF)	MNI	4	ICSD-2	p<0.01, p<0.05, 1080 mm^3[c]	age, sex, and years of education
	Dai et al., 2018[52 a]	39 (10)	39 (13)	48.92±11.38 (NA)	47.87±9.15 (NA)	VBM (GMV)	MNI	6	ICSD-2	p<0.001, 100 vox.[d]	age, sex, years of education, TIV

© 2025 Reimann GM et al. *JAMA Psychiatry*.

	Dai et al., 2020[53 a]	48 (32)	48 (25)	46.48±12.6 (NA)	45.69±12.53 (NA)	rs-fMRI (VMHC)	MNI	6	ICSD-2	p<0.01, 540mm^3[d]	age, gender, years of education
	Liu X et al., 2018[54]	48 (32)	48 (25)	46.48±12.6 (NA)	45.69±12.53 (NA)	rs-fMRI (DC)	MNI	6	ICSD-3	p<0.01, 810mm^3[d]	age, sex, years of education
8	Grau-Rivera et al., 2020[55]	137 (87)	229 (117)	56.7±7.0 (45-75)	56.7±7.4 (45-75)	VBM (GMV)	MNI	7	DSM-IV, ICD-10	p<0.005, 100 vox.[d]	age, sex, education, number of APOE-ε4 alleles, GADS, BMI, age^2, TIV
9	Huang et al., 2017[56]	27 (17)	26 (16)	40.07±11.62 (25-65)	41.19±11.69 (NA)	rs-fMRI (gFCD)	MNI	4	DSM-V	p<0.01, p<0.05 GRF[c]	age, gender
	Zhou et al., 2016b[57]	29 (19)	29 (19)	43.1±11.4 (25-65)	41.6±11.5 (NA)	rs-fMRI (BEN, fALFF)	MNI	16	DSM-IV	p<0.0107, p<0.05 FWE/GRF[c]	age, gender
	Zhou et al., 2016a[58]	27 (10)	27 (10)	42.59±11.59 (25-65)	40.92±11.46 (NA)	rs-fMRI (ALFF)	MNI	12	DSM-IV	p<0.05, p<0.05 FWE[c]	age, gender, mean FD
	Zhou et al., 2018[59]	29 (10)	30 (10)	42.03±11.37 (25-60)	40.03±11.23 (NA)	rs-fMRI (VMHC)	MNI	2	DSM-V	p<0.001, p<0.05 GRF[c]	age, gender
	Zhou F et al., 2020[60]	27 (17)	27 (17)	42.59±11.59 (25-65)	40.92±11.46 (NA)	rs-fMRI (lrFCD, srFCD)	MNI	8	DSM-IV	p<0.001 FWE[c]	age, gender, mean FD
10	Ji et al., 2022[61 a]	32 (20)	34 (21)	37.5±8.9 (18-60)	35.8±7.1 (<60)	rs-fMRI (gFCD)	MNI	2	ICSD-2	p<0.005, p<0.05, 31 vox.[c]	age, sex, mean relative displacements of head motion
	Zhang Y et al., 2021[62]	32 (20)	34 (21)	37.5±8.9 (18-60)	35.8±7.1 (NA)	rs-fMRI (ReHo)	MNI	4	ICSD-2	p<0.005, p<0.05, 27 vox.[c]	NA
11	Jiang et al., 2023[63 a]	52 (35)	30 (23)	36.81±9.60 (18-60)	35.23±7.46 (NA)	rs-fMRI (ALFF)	MNI	3	DSM-V	p>0.005, p<0.05 FDR[c]	age, sex, education level, FD
12	Joo et al., 2013[64]	27 (25)	27 (23)	52.3±7.8 (40-70)	51.7±5.4 (NA)	VBM (GMD)	MNI	24	ICSD-2	p<0.001[d]	age, sex

© 2025 Reimann GM et al. *JAMA Psychiatry*.

13	Kay et al., 2016[65]	44 (24)	40 (25)	37±10 (21-60)	38±11 (21-60)	PET (glucose metabolism during wakefulness vs. non-REM sleep)	MNI	4	DSM-IV	p<0.005, p<0.05 cFWE, 699 vox.[c]	NA
	Kay et al., 2017[66]	32 (13)	30 (19)	37±9 (18-60)	39±11 (18-60)	PET (glucose metabolism during non-REM sleep)	MNI	1	DSM-IV	p<0.005 , p<0.05 cFWE[c]	NA
14	Kim et al., 2017[67]	14 (10)	18 (14)	49.0±12.3 (18-65)	42.7±12.3 (NA)	t-fMRI (sleep-related pictures vs. neutral pictures)	TAL	6	DSM-IV	p<0.001, 20 vox.[d]	NA
	Lee et al., 2022[68 a]	43 (33)	42 (27)	44.6±13.9 (18-65)	39.3±11.9 (NA)	t-fMRI (sleep-related pictures vs. neutral pictures)	MNI	2	ICSD-3	p<0.05 FDR[c]	age
15	Kim YB et al., 2021[69]	28 (20)	16 (11)	57.8±8.7 [n=13], 40.3±13.8 [n=15] (18-70)	41.3±14.7 (18-70)	t-fMRI (insomnia-related sentences vs. neutral sentences)	MNI	3	DSM-V	p<0.001, p<0.05 cFWE, 141 vox.[c]	age, sex, years of education
16	Li Y et al., 2016[70]	30 (13)	30 (NA)	39.36±8.53 (23-47)	36.15±8.61 (21-54)	t-fMRI (spatial memory task)	MNI	14	DSM-IV	p<0.01, 20 vox.[d]	NA
	Li G et al., 2018[71]	15 (9)	15 (8)	37.13±2.530 (22-50)	32.60±2.541 (21-49)	VBM (GMV)	MNI	29	DSM-IV	p<0.001, 50 vox.[d]	NA
17	Li C et al., 2016[72]	55 (31)	44 (33)	39.18±10.34 (25-60)	39.91±9.43 (NA)	rs-fMRI (ALFF)	MNI	6	DSM-IV	p<0.01, p<0.05, 540 mm^3[c]	sex, age, education
	Li M et al., 2018[73]	60 (32)	53 (35)	39.90±10.75 (NA)	39.55±8.77 (NA)	VBM (GMV)	MNI	6	DSM-IV	p<0.001, 25 vox.[d], p<0.05 GRF[c]	age, gender, TIV
	Li S et al., 2018[74]	59 (32)	53 (32)	39.78±10.63 (18-57)	39.70±8.84 (20-59)	rs-fMRI (ICA)	MNI	5	DSM-IV	p<0.05 FWE[c]	NA
	Li et al., 2019[75 a]	38 (22)	44 (33)	40.61±9.43 (<60)	39.91±9.43 (NA)	rs-fMRI (gFCD, SVM)	MNI	6	DSM-IV	p<0.01[d], p<0.05[c]	age, gender, years of education
	Meng et al., 2020[76]	59 (33)	47 (33)	39.27±10.72 (<60)	40.02±9.15 (NA)	rs-fMRI (dALFF)	MNI	2	DSM-IV	p<0.005, p<0.05 GRF[c]	age, sex, years of education
	Wang T et al., 2016[77]	59 (45)	47 (26)	40.0±9.1 (18-57)	39.3±10.7 (20-59)	rs-fMRI (ReHo)	MNI	7	DSM-IV	p<0.05[c]	NA
18	Qiu et al., 2023[78 a]	17 (12)	20 (13)	32.17±5.50 (23-47)	32.40±5.59 (24-46)	rs-fMRI (ReHo)	MNI	7	DSM-IV	p<0.01 FDR, 114 vox.[c]	NA
19	Ran et al., 2017[79]	21 (16)	20 (14)	40.62±7.52 (adult)	38.65±7.40 (adult)	rs-fMRI (ALFF)	MNI	5	DSM-IV	p<0.01, p<0.05, 148 vox.[c]	age, gender, education,

© 2025 Reimann GM et al. *JAMA Psychiatry*.

											GMV, mean head translation motion and rotation motion, SAS
20	Santarnecchi et al., 2018[80]	17 (NA)	17 (NA)	NA (18-45)	NA (NA)	rs-fMRI (ICC)	MNI	1	ICSD-3	p<0.05 FDR[c]	age, gender, BDI
21	Seo et al., 2018[81]	19 (14)	19 (14)	28.96±11.95 (18-65)	29.65±12.81 (18-65)	t-fMRI (2-day fear conditioning and extinction paradigm)	MNI	22	DSM-IV-TR, DSM-V, ICSD-3	p<0.005, 10 vox.[d]	age, sex, scanner type (Trio or Prisma)
22	Shao et al., 2022[82 a,b]	16 (3)	30 (15)	39.00 (18-60)	32.00 (18-60)	t-fMRI (sleep spindle-related connectivity)	MNI	1	DSM-V	P<0.005, p<0.05 FWE[c]	age, gender, education
23	Son et al., 2018[83]	21 (12)	26 (15)	36.6±9.8 (18-60)	33.2±7.1 (NA)	t-fMRI (working memory task)	MNI	2	DSM-V	p<0.001, p<0.01, 34 vox.[c]	NA
24	Wassing et al., 2019[84]	27 (17)	30 (13)	45.5±13.4 (18-70)	42.4±15.8 (18-70)	t-fMRI (novel and relived self-conscious emotions)	MNI	2	DSM-V, ICSD-3	p<0.001, p<0.05 GRF[c]	age, sex, participant's mean intensity difference between the emotional and neutral stimuli
25	Yan et al., 2018a[85]	26 (15)	28 (16)	48.85±12.02 (25-60)	49.07±11.81 (NA)	rs-fMRI (VMHC)	MNI	2	DSM-IV	p<0.01[d], p<0.05, 1296 mm^3[c]	age, gender, education level
	Yan et al., 2018b[86]	26 (15)	28 (16)	48.85±12.02 (25-60)	49.07±11.81 (NA)	rs-fMRI (DC)	MNI	3	DSM-IV	z>1.960, p<0.005 GRF[c]	age, gender, education level, SAS, SDS
26	Yu et al., 2018[87]	48 (29)	51 (22)	38.90±11.71 (18-60)	41.39±13.66 (NA)	rs-fMRI (gFCD)	MNI	12	DSM-IV	p<0.001[d], p<0.0006 FDR, 101 vox.[c]	sex, age, years of education
cf27	Zhao et al., 2020[88]	22 (13)	20 (12)	42.57±14.50 (18-65)	36.21±0.34 (NA)	rs-fMRI (ALFF)	TAL	1	DSM-V	p<0.01, p<0.05, 95 vox.[c]	NA

© 2025 Reimann GM et al. *JAMA Psychiatry*.

28	Zheng et al., 2023[a][89]	38 (31 [n=45])	36 (31 [n=41])	56.00±10.50 [n=45] (45-75)	58.90±8.40 [n=41] (adult)	rs-fMRI (ALFF)	MNI	2	DSM-IV	p<0.001, p<0.05 GRF[c]	NA
**Kleine-Levin Syndrome**											
1	Engström et al., 2009[90] [a]	8 (3)	12 (7)	27±4.2 (NA)	23.9±1.2 (18-30)	t-fMRI (working memory task)	MNI	6	ICSD-2	p<0.001, 5 vox.[d]	NA
**Narcolepsy**											
1	Brenneis et al., 2005[91]	12 (4)	12 (2)	35.0±8.4 (22-72)	35.8±13.2 (NA)	VBM (GMV)	TAL	2	ICSD 1997	P<0.05[c]	sex, global differences in voxel intensities
2	Dauvilliers et al., 2010[92]	21 (11)	21 (11)	40.2 ±18.1 (NA)	40.6±17.8 (NA)	PET (resting glucose metabolism)	TAL	5	Presence of EDS and clear-cut cataplexy	p<0.05 FDR, 101 vox.[c]	NA
	Dauvilliers et al., 2017[37]	16 (4)	19 (3)	30 (17-78)	36 (20-60)	PET (resting glucose metabolism)	TAL	7	Presence of EDS and clear-cut cataplexy	p<0.001, 50 vox.[d], p<0.05, 50 vox.[c]	age
3	Fulong et al., 2018[93]	26 (8)	30 (12)	25.77±6.64 (adult)	25.37±4.31 (adult)	rs-fMRI (fALFF)	MNI	11	ICSD-3	p<0.05 FDR[c]	age, gender, years of education
	Xiao et al., 2019[94]	26 (8)	30 (12)	25.77±6.64 (adult)	25.37±4.31 (adult)	rs-fMRI (ICA)	MNI	4	ICSD-3	P<0.05 FDR, 10 vox.[c]	age, gender, BMI
4	Fulong et al., 2018[93]	25 (5)	30 (6)	14±2.7 (children)	13.3±2.3 (children)	rs-fMRI (fALFF)	MNI	13	ICSD-3	p<0.05 FDR[c]	age, gender, years of education
	Fulong et al., 2020[95] [a]	26 (5)	30 (6)	13.9±2.7 (children)	13.3±2.3 (NA)	rs-fMRI (ICA)	MNI	3	ICSD-3	p<0.01 FDR[c]	NA
5	Gool et al., 2020[96]	12 (4)	11 (4)	33.25±10.5 (18-65)	31.82±13.39 (18-65)	t-fMRI (sustained attention to response task)	MNI	16	ICSD-3	p<0.05 FWE, 20 vox.[c]	NA
	Gool et al., 2022[97] [a]	12 (4)	12 (4)	33.25±10.5 (adult)	32.75±13.16 (adult)	t-fMRI (active sleep resistance paradigm)	MNI	1	ICSD-3	p<0.05 FWE, 21 vox.[c]	NA
	Van Holst et al., 2018[98]	23 (11)	20 (10)	33.83±8.36 (18-60)	36.75±12.14 (18-60)	t-fMRI (Stroop task [classic, food])	MNI	2	ICSD-3	p<0.001, P<0.05 FWE[c]	NA
6	Joo et al., 2004[99]	24 (8)	24 (8)	32 (14-56)	32 (14-56)	PET (resting glucose metabolism)	TAL	8	ICSD	p<0.001, 101 vox.[d]	NA

© 2025 Reimann GM et al. *JAMA Psychiatry*.

	Joo et al., 2009[100]	29 (14)	29 (14)	31.2 (NA)	31.2 (NA)	VBM (GMD)	MNI	11	ICSD-2	p<0.001, 201 vox.[d]	age
7	Kaufmann et al., 2002[101]	12 (6)	32 (16)	36.9±15.8 (22-65)	36.2±14.7 (21-65)	VBM (GMV)	TAL	19	Presence of EDS and clear-cut cataplexy	p<0.001, 100 vox.[d]	age, sex
8	Kim et al., 2009[102]	17 (4)	17 (4)	24.6±4.9 (17-35)	26.6±5.2 (17-35)	VBM (GMV)	TAL	29	Presence of EDS and clear-cut cataplexy	p<0.001[d]	age, sex
9	Reiss et al., 2008[103]	10 (7)	12 (7)	29.8±6.5 (adult)	25.9±4.1 (adult)	t-fMRI (responses to humor)	TAL	2	ICSD-2	p<0.01[c]	gender
10	Scherfler et al., 2012[104]	16 (4)	12 (5)	56.8±10.1 (40-70)	59.8±4.4 (NA)	VBM (GMD)	MNI	3	ICSD-2	p<0.001, p<0.01 FDR[c]	age, TIV
11	Schwartz et al., 2008[105]	12 (6)	12 (6)	32.6±8.3 (19-45)	33.8±6.9 (NA)	t-fMRI (responses to humor)	MNI	17	ICSD-2	p<0.001[d]	NA
12	Tondelli et al., 2018[106 a]	20 (7)	19 (8)	11.19±2.91 (children)	13.05±2.17 (children)	VBM (GMV)	MNI	6	ICSD-3	p<0.05 TFCE[c]	Age, sex, TIV
13	Trotti et al., 2021[38]	14 (10)	9 (6)	30.0±8.3 (NA)	32.2±15.5 (NA)	PET (resting glucose metabolism)	TAL	24	ICSD-3	p<0.005, 100 vox. cFWE[c]	NA
14	Wu et al., 2022[107 a]	18 (8)	18 (7)	21.39±5.37 (14-35)	22.30±4.56 (NA)	rs-fMRI (fALFF)	MNI	14	ICSD-3	p<0.001, p<0.05 GRF[c]	age, gender, educational level, total GMV
15	Xu et al., 2024[108 a]	34 (10)	34 (10)	30±12 (12-59)	29±12 (12-63)	rs-fMRI (fALFF, ReHo)	MNI	17	ICSD-3	p<0.001, p<0.05 FDR[c]	age, sex
**Nightmare Disorder (Parasomnia)**											
1	Shen et al., 2016[109 a]	15 (11)	15 (11)	20.07±1.10 (18-22)	20.60±1.55 (18-23)	rs-fMRI (ReHo)	MNI	6	DSM-V	p<0.01, p<0.05, 13 vox.[c]	NA
**Obstructive Sleep Apnea**											
1	André et al., 2020[110 a]	69 (56 [n=96])	18 (24 [n=31])	69.00±3.98 [n=96] (65<)	69.19±3.53 [n=31] (65<)	PET (resting glucose metabolism)	MNI	1	AHI ≥ 15, ICSD-3	p<0.005, p<0.05 FWE[c]	age, sex, education, BMI, sleep medication use, APOE4 status

© 2025 Reimann GM et al. *JAMA Psychiatry*.

	André et al., 2020[110 a]	96 (56)	31 (24)	69.00±3.98 (65<)	69.19±3.53 (65<)	VBM (GMV)	MNI	1	AHI ≥ 15, ICSD-3	p<0.005, p<0.05 FWE[c]	age, sex, education, BMI, sleep medication use, APOE4 status
2	Ayalon et al., 2006[111]	12 (1)	12 (1)	44.2±11.9 (adult)	43±9.1 (adult)	t-fMRI (verbal learning)	TAL	23	PSG	p<0.05, 12 vox./768 mm[3d]	NA
	Ayalon et al., 2009a[112 a]	14 (1)	14 (1)	45.6±11.7 (NA)	43.6±8.6 (NA)	t-fMRI (Go-NoGo task)	TAL	6	AHI ≥ 10	p<0.05, p<0.05, 12 vox./768mm[3c]	NA
	Ayalon et al., 2009b[113]	14 (1)	14 (1)	45.6±11.7 (NA)	43.6±8.6 (NA)	t-fMRI (sustained attention)	TAL	10	AHI > 10	p<0.05, 4 vox./256mm[3] (subcortical), 12 vox./768mm[3] (cortical)[d]	NA
3	Bai et al., 2021[114 a]	31 (12)	33 (16)	5.65±2.82 (3-10)	6.01±2.43 (2-11)	rs-fMRI (ALFF, fALFF, ReHo)	MNI	5	(OAI > 1 or AHI > 5) and minimal SpO2 < 92%	p<0.001, p<0.05 GRF[c]	age, gender, mean FD
	Ji et al., 2021[115 a]	20 (8)	29 (17)	7.2±3.1 (4-14)	7.7±2.8 (4-14)	rs-fMRI (ALFF, ReHo)	MNI	5	(OAI > 1 or AHI > 5) and minimal SpO2 < 92%	p<0.05, 52 vox. (ALFF), 84 vox. (ReHo)[c]	age, gender, BMI
4	Baima et al., 2020[116 a]	18 (7)	32 (20)	49.9±9.3 (NA)	33.4±9.4 (NA)	VBM (GMV)	MNI	6	PSG	p<0.001[d]	age, sex, TIV
5	Canessa et al., 2011[117 a]	17 (0)	15 (0)	44±7.63 (30-55)	42.15±6.64 (NA)	VBM (GMV)	MNI	6	AHI > 30	p<0.005[d], p<0.05 FWE[c]	age
	Castronovo et al., 2009[118 a]	14 (0)	14 (0)	43.93±7.78 (NA)	42.15±6.64 (NA)	t-fMRI (N-back task (N=2))	MNI	15	AHI > 30	p<0.001, 4 vox.[d]	NA
6	Celle et al., 2009[119 a]	25 (14)	127 (83)	66.1±0.7 (NA)	66.0±0.6 (NA)	VBM (GMV)	MNI	2	ODI > 14	p<0.05[c]	gender, BMI, ambulatory blood pressure, total GMV
7	Chen YS et al., 2022[120 a]	30 (3)	34 (17)	41.93±1.65 (adult)	43.21±2.25 (adult)	VBM (GMV)	MNI	2	(AHI > 5 and OSA symptoms) or AHI > 15	p<0.001, p<0.05 FWE, 228 vox.[c]	age, sex, TIV

© 2025 Reimann GM et al. *JAMA Psychiatry*.

	Lin et al., 2016[121] [a]	21 (3)	15 (4)	40.14±10.80 (adult)	39.80±9.53 (adult)	VBM (GMV)	MNI	2	AHI > 5	p<0.01, p<0.05 FWE, 453 vox.[c]	age, sex, and BMI
8	Fatouleh et al., 2014[122]	17 (2)	15 (3)	55±12.4 (35–69)	53±11.6 (35–68)	VBM (GMV)	MNI	13	AHI > 5	p<0.005, p<0.0005 FDR, 20 vox.[c]	age, sex, total brain volume
9	Fernandes et al., 2022a[123] [a]	34 (7)	34 (7)	68.44±9.98 (adult)	69.02±6.08 (adult)	PET (resting glucose metabolism)	TAL	9	AHI ≥ 15	p<0.05 FDR/FWE[c]	sex, MMSE
	Fernandes et al., 2022b[124] [a]	20 (6)	15 (7)	58.75±3.53 (adult)	63.8±8.46 (adult)	PET (resting glucose metabolism)	TAL	19	AHI ≥ 15, ICSD-3	p<0.05 FDR/ p<0.0001 FWE[c]	age, sex, CSF levels
10	Gao et al., 2023[125] [a]	50 (0)	50 (0)	42.90±9.25 (18-60)	43.48±9.54 (18-60)	DBM (brain volume)	MNI	3	AHI ≥ 30	p<0.001, 121 vox.[d]	age, educational level
11	Harper et al., 2003[126]	10 (0)	16 (0)	46±12 (28-64)	47±10 (30-60)	t-fMRI (responses to cold pressor challenges)	MNI	19	Confirmed sleep laboratory diagnosis	NA	NA
	Henderson et al., 2003[127]	8 (0)	15 (0)	44±11.3 (31-63)	45±11.6 (30-58)	t-fMRI (responses to the Valsalva maneuver)	MNI	12	AASM clinical practice guideline, PSG	p<0.05[d]	NA
	Macey et al., 2003[128]	9 (0)	16 (0)	45±12 (28-64)	46±11 (29-63)	t-fMRI (responses to expiratory loading)	MNI	12	PSG	P<0.01[d]	NA
	Macey et al., 2006[129]	7 (0)	11 (0)	46±13.2 (28-63)	47±9.9 (30-63)	t-fMRI (responses to inspiratory loading)	MNI	37	PSG	p<0.05, 12 vox.[d]	NA
12	Huynh et al., 2014[130] [a]	27 (0)	7 (0)	44.0±2.0 [n=13], 42.9±2.2 [n=14] (NA)	41.4±3.1 (NA)	VBM (GMV)	MNI	4	AHI > 15	p<0.001, 20 vox.[d]	NA
	Prilipko et al., 2011[131]	17 (0)	7 (0)	43.2±8.4 (NA)	NA (NA)	t-fMRI (N-back task (visuospatial, 4 levels))	TAL	25	AHI ≥ 15	p<0.001, 10 vox.[d]	NA
13	Joo et al., 2010[132]	36 (0)	31 (0)	44.7±6.7 (18-55)	44.8±5.4 (NA)	VBM (GMD)	MNI	27	AHI > 30	p<0.05 FDR, 201 vox.[c]	age
	Kim et al., 2016[133] [a]	21 (0)	59 (0)	49.8±7.7 (31-61)	44.3±10.1 (25-76)	DBM (brain volume)	MNI	17	AHI > 15	p<0.05 FDR[c]	age, depressive mood scores
14	Kang et al., 2020[134] [a]	14 (0)	16 (0)	48.71±6.71 (adult)	44.75±9.26 (adult)	rs-fMRI (ALFF, ReHo)	MNI	12	AHI ≥5 accompanied by	p<0.05, p<0.05 GRF, 390 vox.[c]	BMI

© 2025 Reimann GM et al. *JAMA Psychiatry*.

									daytime sleepiness and other symptoms		
15	Li et al., 2015[135]	25 (0)	25 (0)	39.4±1.7 (23-59)	39.5±1.6 (NA)	rs-fMRI (ALFF)	MNI	2	AHI > 30	p<0.001, p<0.05 FDR, 270 mm^3[c]	age, education
	Li H et al., 2016[136] [a]	36 (0)	40 (0)	39.0±8.1 (21-59)	38.8±11.2 (21-59)	rs-fMRI (DC)	MNI	46	AASM clinical practice guideline	p<0.001, 41 vox.[c]	age, ESS, years of educational, mean FD
	Li H et al., 2021[137] [a]	21 (1)	21 (1)	40.1±8.4 (20-60)	40.1±8.6 (NA)	rs-fMRI (ReHo)	MNI	13	AASM clinical practice guideline, AHI > 15	p<0.01, p<0.05 GRF[c]	BMI, educational years
	Li P et al., 2022[138] [a]	21 (1)	21 (1)	40.1±8.4 (22-60)	40.1±8.6 (22-60)	rs-fMRI (DC)	MNI	5	AHI > 15	p<0.01, 40 vox.[c]	NA
	Li K et al., 2022[139] [a]	79 (0)	84 (0)	38.5±9.3 (18-60)	45.3±12.1 (NA)	rs-fMRI (dReHo)	MNI	6	AHI > 15	p<0.01, p<0.05 GRF[c]	age, head movement parameters, years of education
	Peng et al., 2014[140] [a]	25 (0)	25 (0)	39.4±1.7 (23-59)	39.5±1.6 (NA)	rs-fMRI (ReHo)	MNI	8	AHI > 30	p<0.001, p<0.05 FDR, 405mm^3[c]	age
	Shu et al., 2022[141] [a]	26 (0)	26 (0)	36.15±9.48 (18-65)	39.84±11.18 (18-65)	rs-fMRI (dALFF)	MNI	1	AHI > 30	p<0.005, p<0.05 GRF[c]	age, education level, head motion
	Xie et al., 2022[142] [a]	52 (0)	61 (0)	37 (18-65)	42 (18-65)	rs-fMRI (PerAF)	MNI	4	AHI ≥ 15	p<0.001, p<0.05 GRF[c]	age, head motion, years of education
	Zeng et al., 2022[143] [a]	52 (0)	62 (0)	37.71±9.90 (18-65)	39.69±8.77 (NA)	rs-fMRI (ALFF)	MNI	37	AASM clinical practice guideline, AHI > 15	p<0.01, p<0.05 GRF[c]	age, years of education
16	Liu YT et al., 2018[144] [a]	29 (6)	26 (8)	39.62±9.95 (20-60)	34.46±9.97 (20-60)	rs-fMRI (VMHC)	MNI	4	AASM criteria	p<0.05, 63 vox.[c]	NA
17	Morrell et al., 2010[145]	60 (3)	60 (5)	47.3±12.1 (NA)	46.1±11.5 (NA)	VBM (GMV)	MNI	2	AHI > 30	p<0.05 FDR[c]	age, sex, site, TIV

© 2025 Reimann GM et al. *JAMA Psychiatry*.

18	Philby et al., 2017[146 a]	16 (8)	200 (116)	8.1±2.2 (children)	8.2±2.0 (children)	VBM (GMV)	MNI	26	AHI > 2 and minimal SpO2 < 92% and/or a respiratory arousal index > 2	p<0.05 FWE, 92.4 vox.[c]	sex, age, TIV
19	Qin et al., 2020[147 a]	36 (0)	38 (0)	48.50±7.15 (NA)	46.13±7.02 (NA)	rs-fMRI (ALFF, ReHo)	MNI	18	AASM clinical practice guideline (2007)	p<0.001, 23 vox.[c]	NA
20	Ruan et al., 2023[148]	20 (2)	20 (3)	44.70±8.95 (NA)	46.80±9.23 (NA)	rs-fMRI (ReHo)	MNI	5	PSG	p<0.05[d]	NA
21	Santarnecchi et al., 2013[149]	19 (3)	19 (5)	43.2±8 (30-55)	41±6 (NA)	rs-fMRI (ReHo)	MNI	16	AHI > 30	p<0.01, p<0.05 FDR[c]	age, gender, total brain volume, BMI
	Santarnecchi et al., 2022[150 a]	20 (3)	20 (4)	42.9 ±7 (30-55)	41 ±6 (NA)	rs-fMRI (fALFF)	MNI	7	AHI > 30	p<0.05, p<0.05, 27 vox.[c]	age, gender, BMI
22	Sun et al., 2022[151 a]	30 (0)	19 (0)	42.50±5.82 (20-60)	40.32±2.89 (20-60)	rs-fMRI (ALFF)	MNI	17	AHI>30	p<0.001, p<0.05 GRF[c]	age, education level, head motion
	Sun et al., 2023[152 a]	30 (0)	19 (0)	42.5±5.8 (20-60)	40.3±2.9 (20-60)	rs-fMRI (fALFF, ReHo)	MNI	4	AHI > 30	p<0.01, p<0.05 GRF[c]	age, education level, head motion
23	Torelli et al., 2011[153]	16 (3)	14 (5)	55.8±6.7 (NA)	57.6±5.2 (NA)	VBM (GMV)	MNI	1	AHI ≥ 15	p<0.05 FWE[c]	NA
24	Xiao et al., 2022[154 a]	18 (5)	36 (9)	43.00±15.73 (NA)	40.61±12.01 (NA)	VBM (GMV)	MNI	3	AHI > 15	p<0.001, p<0.05 FWE, 166 vox.[c]	age, sex, years of education, TIV
25	Yaouhi et al., 2009[155]	16 (1)	14 (1)	54.75±5.71 (NA)	52.71±7.01 (NA)	PET (resting glucose metabolism), VBM (GMD)	MNI	10	AHI > 10	P<0.005, p<0.05[c]	NA
26	Yu et al., 2023[156 a]	52 (25)	31 (17)	8.77±1.436 (children)	9.13±1.176 (children)	VBM (GMV)	MNI	1	OAHI > 1	p<0.001 GRF[c]	age, sex, BMI
27	Zhang et al., 2013[157]	24 (0)	21 (0)	44.6±7.4 (30-60)	40.6±11.4 (30-60)	rs-fMRI (ICA), VBM (GMV)	MNI	12	AHI ≥ 15	p<0.0001[d], p<0.05 FWE[c]	age

© 2025 Reimann GM et al. *JAMA Psychiatry*.

28	Zhou L et al., 2020[158 a]	33 (3)	22 (4)	43.64±10.78 (NA)	39.73±11.22 (NA)	rs-fMRI (ReHo)	MNI	4	AASM clinical practice guideline (2015), PSG	p<0.001, p<0.05 GRF[c]	age, sex, education level, BMI
**Periodic Limb Movement Disorder**											
1	Fernandes et al., 2022b[124 a]	12 (5)	15 (7)	72.23±5.29 (adult)	63.8±8.46 (adult)	PET (resting glucose metabolism)	TAL	3	PLMI ≥ 15, ICSD-3	p<0.05 FDR/ p<0.0001 FWE[c]	age, sex, CSF levels
**(Idiopathic/Isolated) Rapid Eye Movement Sleep Behaviour Disorder**											
1	Bourgouin et al., 2019[159 a]	16 (4)	31 (10)	65.65±6.61 (NA)	63.28±8.37 (NA)	VBM (GMV)	MNI	1	ICSD-3	p<0.001, 100 vox.[d]	age, gender, TIV, education, MCI status
	Bourgouin et al., 2019[159 a]	17 (5)	31 (10)	67.35±5.32 (NA)	63.28±8.35 (NA)	VBM (GMV)	MNI	13	ICSD-3	p<0.001, 100 vox.[d], p<0.05 FWE[c]	age, gender, TIV, education, MCI status
	Bourgouin et al., 2019[159 a]	30 (6)	31 (10)	66.48±6.37 (NA)	63.28±8.38 (NA)	VBM (GMV)	MNI	1	ICSD-3	p<0.001, 100 vox.[d]	age, gender, TIV, education, MCI status
	Bourgouin et al., 2019[159 a]	27 (5)	31 (10)	65.85±6.25 (NA)	63.28±8.36 (NA)	VBM (GMV)	MNI	1	ICSD-3	p<0.001, 100 vox.[d]	age, gender, TIV, education, MCI status
	Rahayel et al., 2015[160 a]	24 (4)	42 (14)	64.2±7.0 (NA)	63.3±7.1 (NA)	VBM (GMV)	MNI	1	ICSD-2	p<0.05[c]	age, gender, education, MoCA
	Rahayel et al., 2018[161 a]	41 (8)	41 (15)	65.2±6.4 (NA)	63.4±9.0 (NA)	VBM (GMV)	MNI	6	ICSD-2	p<0.05 FWE/TFCE[c]	age, TIV
2	Brcina et al., 2021[162 a]	13 (0)	13 (0)	63.69±12.13 (32-78)	60.85±13.84 (31-77)	t-fMRI (bilateral hand-movement task)	MNI	9	AASM criteria	p<0.001, p<0.05 FWE[c]	age
3	Chen M et al., 2022[163 a]	27 (8)	33 (13)	65.89±8.54 (NA)	68.25±7.80 (NA)	rs-fMRI (ALFF), VBM (GMV)	MNI	21	ICSD-2	p<0.001, p<0.05 GRF[c]	age, sex, (GMD for ALFF)

© 2025 Reimann GM et al. *JAMA Psychiatry*.

4	Ge et al., 2015[164 a]	21 (4)	21 (4)	65.0±5.6 (NA)	62.5±7.5 (NA)	PET (resting glucose metabolism)	MNI	18	ICSD-2	p<0.001, 60 vox./460mm^3[d], p<0.05 FWE[c]	NA
	Wu et al., 2014[165 a]	21 (4)	21 (4)	65.0±5.6 (NA)	62.5±7.5 (NA)	PET (RBD-RP)	MNI	26	ICSD-2	p<0.01[d]	NA
5	Han et al., 2019[166 a]	19 (3)	20 (15)	66.6±7.0 (NA)	63.7±5.9 (NA)	VBM (GMV)	MNI	6	ICSD-3	p<0.01, 160 vox.[d]	sex, TIV
6	Hanyu et al., 2012[167 a]	20 (3)	18 (9)	66±7 (NA)	71±8 (55-82)	VBM (GMV)	MNI	8	ICSD-2	p<0.05, 100 vox.[c]	NA
7	Jiang et al., 2021[168 a]	24 (4)	26 (12)	66.04±5.73 (51-74)	62.73±6.33 (51-74)	VBM (GMV)	MNI	6	ICSD-3	ANOVA: p<0.001, p<0.05[c], post-hoc t-test: p<0.017 Bonferroni[c]	age, gender, education, TIV
	Jiang et al., 2022[169 a]	23 (5)	26 (12)	66.65±7.06 (50-75)	62.73±6.33 (NA)	rs-fMRI (ReHo)	MNI	12	AASM criteria, ICSD-3	p<0.005, p<0.01[c]	age, sex, education, GMV
8	Kim R et al., 2021[170 a]	25 (13)	24 (17)	69.2±5.3 (NA)	69.5±4.3 (NA)	PET (RBD-RP)	MNI	14	ICSD-2	p<0.05[d]	NA
	Lim et al., 2016[171 a]	24 (12)	25 (12)	69.8±6.4 (NA)	68.5±6.6 (NA)	VBM (GMV)	MNI	7	RBDSQ + audio-visual PSG	p<0.001, 50 vox.[d]	age, gender, Parkinson's disease duration, Hoehn and Yahr stage
	Yoon et al., 2019[172 a]	28 (14)	24 (17)	69.8±5.6 (50-80)	69.5±4.3 (NA)	PET (RBD-RP)	MNI	14	ICSD-2	p<0.05[d]	NA
9	Li et al., 2017[173 a]	18 (8)	19 (8)	63.9±8.2 (NA)	62.7±8.1 (NA)	rs-fMRI (ALFF)	MNI	3	AASM criteria	p<0.005, p<0.05, 28 vox.[c]	NA
10	Li et al., 2020[174 a]	15 (6)	15 (6)	64.27±1.87 (NA)	64.80±1.83 (NA)	rs-fMRI (ReHo)	MNI	3	ICSD-3	p<0.05 FDR, 11 vox.[c]	age, gender, education
11	Mattioli et al., 2021[175 a]	22 (4)	42 (NA)	69.6±6.1 (NA)	69.6±8.5 (NA)	PET (resting glucose metabolism)	TAL	10	ICSD-3	p<0.0001, p<0.5 FWE, 100 vox.[c]	age
12	Rahayel et al., 2019[176 a]	15 (5)	41 (6)	66.7±7.6 (45-85)	63.3±8.1 (45-85)	VBM (GMV), DBM (brain volume)	MNI	8	ICSD-3	p<0.05 FWE/TFCE[c]	age, gender, education, (TIV for VBM)

© 2025 Reimann GM et al. *JAMA Psychiatry*.

13	Salsone et al., 2014[177 a]	11 (3)	18 (5)	66.6±7.4 (NA)	65.1±7.8 (NA)	VBM (GMV)	MNI	2	RBD-1Q + audio-visual PSG	p<0.001, 10 vox.[d], p<0.05 FWE[c]	age, TIV
14	Scherfler et al., 2011[178 a]	26 (5)	14 (4)	67.4±4.9 (NA)	64.5±5.2 (NA)	VBM (GMD)	TAL	2	audio-visual PSG	p<0.001[c]	age, TIV
15	Woo et al., 2023[179 a]	51 (22)	39 (NA)	70.24±5.985 (50-80)	NA (NA)	VBM (GMV)	MNI	3	ICSD-2	p<0.01 FWE/TFCE[c]	age, TIV
16	Zhang HJ et al., 2021[180 a]	21 (7)	22 (13)	64 (NA)	60 (NA)	rs-fMRI (ReHo)	MNI	4	ICSD-3	p<0.01, p<0.05 GRF, 11 vox.[c]	age, gender, education years, head motion parameters
**Restless Legs Syndrome**											
1	Etgen et al., 2005[181 a]	51 (38)	51 (38)	53.3±8.0 [n=28], 59.3±10.1[n=23] (NA)	52±8.3 [n=28], 59±10.2 [n=23] (NA)	VBM (GMV)	TAL	2	IRLSSG	p<0.001[d]	NA
2	Hornyak et al., 2007[182 a]	14 (9)	14 (9)	49.6±12.5 (NA)	49.1±12.8 (NA)	VBM (GMD)	MNI	4	IRLSSG	p<0.05 FDR, 5 vox.[c]	global GM
3	Li T et al., 2018[183 a]	20 (15)	18 (13)	56.60±9.86 (NA)	57.28±4.63 (NA)	VBM (GMV)	MNI	5	IRLSSG	p<0.05 FDR, 100 vox.[c]	NA
	Liu et al., 2015[184 a,b]	15 (12)	14 (8)	56.53±9.75 (35-72)	59.43±9.83 (35-71)	rs-fMRI (ALFF)	MNI	9	IRLSSG	p<0.05, 2214mm^3[c]	age, gender
	Liu C et al., 2018[185 a]	16 (12)	26 (7)	55±10.35 (35-72)	58.62±7 (35-71)	rs-fMRI (gFCD)	MNI	5	IRLSSG	p<0.001, p<0.05[c]	age, gender
	Zhang et al., 2023[186 a]	32 (23)	33 (21)	59.38±8.69 (40-77)	60.18±6.82 (35-71)	rs-fMRI (dALFF)	MNI	2	IRLSSG	p<0.001, p<0.05[c]	NA
4	Stefani et al., 2019[187 a]	87 (47)	87 (47)	52 (18<)	50 (18<)	VBM (GMV)	MNI	4	IRLSSG	p<0.001 FWE[c]	age, TIV, treatment
	Tuovinen et al., 2021[188 a]	82 (43)	82 (43)	51.9±10.8 (18-75)	50.2±10.0 (18-75)	rs-fMRI (ICA)	MNI	10	IRLSSG	p<0.05 TFCE/FDR[c]	NA
5	Yang et al., 2018[189 a]	27 (20)	45 (33)	44.5±12.5 (NA)	40.6±10.7 (NA)	VBM (GMV)	MNI	5	IRLSSG	p<0.005, p<0.05 FWE, 210 vox.[c]	age, gender, TIV
6	Zhuo et al., 2017[190 a]	32 (22 [n=35])	26 (15 [n=27])	51.83±16.48 [n=35]	49±16.27 (25-75)	rs-fMRI (ReHo)	MNI	12	IRLSSG	p<0.05[c]	NA

© 2025 Reimann GM et al. *JAMA Psychiatry*.

				(NA)							
**Sleepwalking/Somnambulism (Parasomnia)**											
1	Heidbreder et al., 2017[191 a]	14 (6)	14 (6)	29±4.2 (21-39)	29.5±4 (24-39)	VBM (GMV)	MNI	2	ICSD-3	p<0.001 FWE[c]	age, TIV

[a] Newly added experiments, that have not been included in any of our previous meta-analyses
[b] Coordinates received from the authors
[c] Corrected p-value and threshold
[d] Uncorrected p-value and threshold

Abbreviations: AASM: American Academy of Sleep Medicine; AHI: apnea-hypopnea index; ALFF: amplitude of low-frequency fluctuations; APOE4: apolipoprotein E4; BDI: Beck Depression Inventory; BEN: brain entropy ; BMI: body mass index; cFWE: cluster-level family-wise error; CSF: cerebrospinal fluid; dALFF: dynamic amplitude of low-frequency fluctuations; DBM: deformation-based morphometry; DC: degree centrality; dfALFF: dynamic fractional amplitude of low-frequency fluctuations; dReHo: dynamic regional homogeneity; DSM-IV / DSM-IV-TR / DSM-V: Diagnostic and Statistical Manual of Mental Disorders, 4th Edition/4th Edition, Text Revision/5th Edition; ESS: Epworth Sleepiness Scale; fALFF: fractional amplitude of low-frequency fluctuations; FD: framewise displacement; FDR: false discovery rate; FWE: family-wise error; gFCD: global functional connectivity density (functional connectivity strength); GM / GMD / GMV: gray matter (density/volume); GRF: Gaussian random field; ICA: independent component analysis; ICC: intrinsic connectivity contrast , ICSD-2 / ICSD-3: International Classification of Sleep Disorders, 2nd Edition/3rd Edition; IRLSSG: International Restless Legs Syndrome Study Group; lrFCD: long-range functional connectivity density; MCI: mild cognitive impairment; MMSE: Mini-Mental State Examination; MNI: Montreal Neurological Institute; MoCA: Montreal Cognitive Assessment; NA: not available; Nr.: Number; OAHI: obstructive apnea/hypopnea index; OAI: obstructive apnea index; PerAF: percent amplitude of fluctuation; PET: positron emission tomography; PLMI: Periodic limb movement index; PSG: polysomnography; RBD-RP: RBD-related covariance pattern; ReHo: regional homogeneity; REM: rapid eye movement; rs-fMRI: resting-state functional magnetic resonance imaging; SAS: Self-Rating Anxiety Scale; SDS: Self-Rating Depression Scale; SpO2: peripheral capillary oxygen saturation; srFCD: short-range/local functional connectivity density; SVM: support vector machine; TAL: Talairach; TFCE: threshold-free cluster enhancement; t-fMRI: task-based functional magnetic resonance imaging; TIV: total intracranial volume; VBM: voxel-based morphometry; VMHC: voxel-mirrored homotopic connectivity; vox.: voxels

© 2025 Reimann GM et al. *JAMA Psychiatry*.

eTable 3. Included Sleep Deprivation Experiments

	Author, Year	Study design	Nr. of Subjects (Female)		Age, Mean ± SD (Range)		Imaging Modality	Brain Space	Nr. of Foci	Deprived Sleep	P-value	Covariates
			Before	After	Before	After						
**Total Sleep Deprivation**												
1	Almklov et al., 2015[192 a]	before-after	56 (41)		28.4±4.8 [n=28], 67.6±5.4 [n=28] (18-39, 59<)		t-fMRI (Go-NoGo task)	TAL	4	36h	p<0.01, p<0.006, 4 vox.[b]	NA
2	Bell-McGinty et al., 2004[193]	before-after	19 (5)		25.05±2.7 [n=19] (21-30)		t-fMRI (non-verbal recognition task)	TAL	12	48h	p<0.05[c]	NA
	Habeck et al., 2004[194]	before-after	18 (NA)		26.3±4.9 [n=18] (20-35)		t-fMRI (delayed-match-to-sample task)	TAL	62	48h	p<0.0268[b]	NA
3	Ben Simon et al., 2018[195 a]	before-after	18 (9)		20.2±1.5 (18-24)		t-fMRI (social distance [human vs. object])	MNI	18	24h	p<0.001[c]	NA
	Ben Simon et al., 2020[196 a]	before-after	18 (9)		20.2±1.5 (18-24)		t-fMRI (emotional [adversive] vs. neutral videos)	MNI	22	24h	p<0.005, 5 vox.[c]	NA
	Ben Simon et al., 2022[197 a]	before-after	24 (13)		20.6±0.35 (18-26)		t-fMRI (social judgment/mentalizing)	MNI	31	24h	p<0.005, 5 vox.[c]	NA
	Greer et al., 2016[198]	case-control	14 (10)	15 (7)	20.86±1.8 [n=7], 20.57±1.3 [n=7] (NA)	20.86±2.9 [n=7], 19.63±1.2 [n=8] (NA)	t-fMRI (monetary incentive delay task)	MNI	16	24.9h±1.2 h	p<0.001, 20mm[3c]	NA
4	Cai et al., 2021[199 a]	before-after	42 (23)		21.57±2.25 (NA)		rs-fMRI (ALFF)	MNI	7	24 h	p<0.001, p<0.05 GRF[b]	NA

© 2025 Reimann GM et al. *JAMA Psychiatry*.

5	Cao et al., 2024[200 a]	before-after	90 (0)	21.3±1.7 (18-28)	rs-fMRI (ALFF)	MNI	8	36h	p<0.0001, p<0.05 FDR[b]	NA
6	Chee et al., 2004[201]	before-after	14 (5)	23 (19-24)	t-fMRI (verbal working memory)	TAL	8	22.9h±0.8 h	p<0.005[c]	NA
	Choo et al., 2005[202]	before-after	12 (5 [n=14])	21.8±0.8 [n=14] (NA)	t-fMRI (N-back task)	TAL	6	24.4h±0.3 h	p<0.001[c]	NA
7	Chen et al., 2018[203 a]	before-after	22 (22)	26.91±6.05 (NA)	rs-fMRI (ALFF)	MNI	7	24h	p<0.01, p<0.05, 20 vox.[b]	age, gender, education
	Dai et al., 2012[204]	before-after	8 (8)	22 [n=16] (21-25)	rs-fMRI (ReHo)	MNI	6	24h	P<0.001, 270mm[3c]	NA
	Dai et al., 2012[204]	before-after	8 (0)	22 [n=16] (21-25)	rs-fMRI (ReHo)	MNI	1	24h	P<0.001, 270mm[3c]	NA
	Dai et al., 2018[52 a]	before-after	22 (13)	21.91±1.38 (NA)	VBM (GMV)	MNI	11	24h, 36h	p<0.05 FWE, 100 vox.[b]	NA
	Gao et al., 2015[205]	before-after	16 (8)	22.1±0.8 (NA)	rs-fMRI (ALFF)	MNI	25	24h	p<0.001, p<0.05, 39 vox./1053mm[3b]	NA
	Kong et al., 2018[206 a]	before-after	20 (12)	20.6±1.9 (NA)	rs-fMRI (lrFCD, srFCD)	MNI	15	24h	p<0.05, p<0.05[b]	NA
	Zeng et al., 2021[207 a]	before-after	20 (8)	22.25±1.12 (NA)	rs-fMRI (PerAF)	MNI	5	36h	p<0.001, p<0.001 GRF, 7101mm[3b]	NA
8	Chen et al., 2023[208 a]	before-after	16 (0)	21.79±2.37 [n=19] (adult)	rs-fMRI (ReHo)	MNI	1	36h	p<0.001, p<0.05 FDR[b]	NA
	Dai et al., 2023[209 a]	before-after	41 (0)	20.55±1.36 [n=28], 22.71±2.21 [n=20] (18-30)	t-fMRI (anticipation task)	MNI	5	36h	p<0.0001, 16 vox.[c], p<0.05 FWE[b]	age

© 2025 Reimann GM et al. *JAMA Psychiatry*.

	Liu et al., 2014[210 a]	before-after	11 (0)	24.83±2.88 [n=12] (20-32)	VBM (GMV)	MNI	8	72h±0.8h	p<0.001, 40mm[3c]	NA
	Shao et al., 2009[211 a]	before-after	13 (0)	25.9±2.3 [n=14] (18-28)	t-fMRI (Go-NoGo task [visual])	TAL	13	36h	p<0.05, p<0.05, 680mm[3b]	NA
9	Czisch et al., 2012[212]	before-after	14 (6)	25.0±2.9 (21-30)	t-fMRI (oddball task [acoustic])	MNI	5	36h	p<0.005, p<0.05 FWE[b]	NA
10	Dai et al., 2015[213 a]	before-after	12 (0)	24.83±2.88 (20-32)	rs-fMRI (ALFF, ICA)	MNI	1	72h	P<0.005, p<0.05[b]	NA
11	Drummond et al., 1999[214 a]	before-after	13 (6)	27.2±4.4 (21-35)	t-fMRI (serial subtraction / arithmetic performance)	TAL	16	35h	p<0.025, p<0.0005, 6 vox./506mm[3b]	NA
	Drummond et al., 2001[215 a]	before-after	13 (6)	27.2±4.4 (NA)	t-fMRI (divided attention [verbal learning + arithmetic])	TAL	11	35h	p<0.001[b]	NA
12	Drummond et al., 2004[216 a]	before-after	16 (7)	27.6±6.1 (adult)	t-fMRI (Baddeley's logical reasoning task / grammatical transformation task)	TAL	18	35h±0.7h	p<0.01 Bonferroni, 9 vox./576mm[3b]	NA
	Drummond et al., 2005[217]	before-after	32 (14)	27.6±6.6 (18-45)	t-fMRI (verbal learning task)	TAL	5	35.7h±0.8h	p<0.05, 12 vox./768mm[3c]	NA
13	Fischer et al., 2005[218 s]	before-after	10 (12 [n=20])	21.4±4.1 [n=20] (18-28)	t-fMRI (learning retention [sequential finger-tapping])	MNI	12	24h	p<0.005[c]	NA
14	Gazdzinski et al., 2021[219 a]	before-after	17 (0)	32.9±4.4 (NA)	t-fMRI (detection of visual feature conjunctions)	MNI	1	1 night	z>3.1, p<0.05[b]	NA
15	Gazes et al., 2012[220 a]	before-after	19 (8)	23±3.6 (20-34)	t-fMRI (dual-tasking [continuous visuomotor tracking / colour-matching visual detection])	TAL	39	49h	p<0.05 Bonferroni, 20 vox.[b]	NA

© 2025 Reimann GM et al. *JAMA Psychiatry*.

16	Goldstein et al., 2013[221 a]	before-after	18 (9)		19.6±1.45 (18-30)	t-fMRI (emotional-anticipation task)	MNI	25	24h	p<0.001[c]	NA
	Gujar et al., 2010[222]	case-control	14 (14 [n=28])	12 (14 [n=28])	22.3±2.8 [n=28] (18-30)	t-fMRI (memory encoding task)	MNI	2	35.2h±0.95h	p<0.001, 5 vox.[c]	NA
	Gujar et al., 2011[223 a]	case-control	14 (7)	13 (7)	23.6±1.4 [n=27] (18-30)	t-fMRI (emotional visual stimuli)	MNI	9	31.9h±1.31h	p<0.001, 5 vox.[c]	NA
	Yoo et al., 2007[224 a]	case-control	14 (14 [n=28])	14 (14 [n=28])	22.3±2.8 [n=28] (NA)	t-fMRI (episodic memory encoding)	MNI	8	~35h	p<0.001, 5 vox.[c]	NA
17	Guo et al., 2019[225 a]	before-after	17 (13)		23.00±1.37 (21-26)	rs-fMRI (ALFF)	MNI	19	24h	p<0.05, 213 vox.[b]	NA
18	Huang et al., 2022[226 a]	before-after	24 (24)		20±0.81 (NA)	rs-fMRI (FS)	MNI	11	24h	p<0.001, p<0.05 GRF[b]	FD value
	Yan et al., 2022[227 a]	before-after	20 (20)		20±0.81 (NA)	rs-fMRI (dALFF)	MNI	30	24h	p<0.05 FDR[b]	FD value
19	Jackson et al., 2011[228 a]	before-after	12 (0)		42.6±9.6 (27-56)	t-fMRI (cross-modal divided attention task [visual and auditory])	MNI	1	27h	p<0.001, P<0.05 FWE[b]	NA
20	Klumpers et al., 2015[229]	before-after	12 (6)	11 (NA)	28.5±4.8 [n=6, male], 29.2±10.2 [n=6, female] (adult)	t-fMRI (semantic emotional classification)	MNI	12	25h	p<0.005, 10 vox.[c]	NA
21	Kong et al., 2012[230]	before-after	22 (11)		20±1.3 (NA)	t-fMRI (attending face vs. house)	TAL	2	1 night	p<0.000001[c]	NA
22	Lei et al., 2017[231 a]	before-after	31 (0)		23.1±1.9 [n=37] (18-28)	t-fMRI (balloon analogue risk task)	MNI	6	36-40h	p<0.005, 45 vox.[b]	NA
	Shen et al., 2017[232 a]	before-after	26 (0)		23.1±1.9 [n=37] (adult)	rs-fMRI (group-SSR)	MNI	21	36h	p<0.01 FDR[b]	NA

© 2025 Reimann GM et al. *JAMA Psychiatry*.

23	Lythe et al., 2012[233]	before-after	20 (0)	26.7±6.7 (NA)	t-fMRI (N-back task)	MNI	2	30.8h±1.3 h	p<0.001, p<0.05[b]	NA
24	Mao et al., 2023[234 a]	before-after	56 (30)	32.59±7.91 (21-50)	t-fMRI (risky decision-making)	MNI	14	24h	p<0.001, p<0.05 FWE[b]	NA
25	Menz et al., 2012[235]	before-after	22 (0)	26.6±4.22 (NA)	t-fMRI (risky choice task)	MNI	41	24h	p<0.001, 10 vox./80mm[3c]	task order (control/sleep-deprived)
	Rihm et al., 2019[236 a]	before-after	32 (0)	26.13±3.80 (19-33)	t-fMRI (value-based decision making [food vs. trinkets])	MNI	35	1 night	p<0.001, 5 vox.[c]	NA
26	Mi et al., 2023[237 a]	before-after	20 (14 [n=22])	19.05±1.19 [n=22] (18-28)	t-fMRI (social feedback task)	MNI	8	24h	p<0.006, p<0.05 FWE[b]	NA
27	Mu et al., 2005a[238 a]	before-after	10 (0)	27.8±1.7 (18-45)	t-fMRI (Sternberg working memory task)	MNI	4	30h + SR	p<0.01, p<0.05[b]	NA
	Mu et al., 2005a[238 a]	before-after	10 (0)	28.2±1.9 (18-45)	t-fMRI (Sternberg working memory task)	MNI	6	30h + SR	p<0.01, p<0.05[b]	NA
	Mu et al., 2005b[239]	before-after	33 (0)	28.6±6.6 (NA)	t-fMRI (Sternberg working memory task)	MNI	22	30h + SR	p<0.01, p<0.05[b]	NA
28	Mullin et al., 2013[240]	before-after	25 (16)	23.1±1.6 [n=27] (18-25)	t-fMRI (monetary reward task)	MNI	12	25.5-27h	p<0.005, 20 vox.[c]	NA
29	Nakashima et al., 2018[241 a]	before-after	12 (3)	31.5±8.4 (NA)	t-fMRI (task switching)	MNI	4	24h	p<0.001, p<0.05 FWE, 106 vox.[b]	NA
30	Shermohammed et al., 2020[242 a]	before-after	34 (17)	NA (18-30)	t-fMRI (cognitive reappraisal task [emotion regulation])	MNI	7	24h	p<0.05 FWE[b]	NA
31	Sun et al., 2020[243 a]	before-after	23 (13)	20.3±1.64 (17-23)	VBM (GMD)	MNI	3	24h	p<0.05 TFCE[b]	NA

© 2025 Reimann GM et al. *JAMA Psychiatry*.

	Zhao et al., 2019[244 a]	before-after	20 (9)	19.9±1.77 (17-23)	t-fMRI (stop-signal task)	MNI	33	24h	p<0.001 FDR, 10 vox.[b]	age, gender
32	Thomas et al., 2000[245]	before-after	17 (0)	24.7±2.8 (21-29)	PET (glucose metabolism during serial addition subtraction task)	TAL	53	24h	p<0.05[b]	NA
33	Vartanian et al., 2014[246]	before-after	13 (3)	32.23±8.45 (NA)	t-fMRI (divergent thinking task / cognitive information processing)	MNI	2	1 night	p<0.001, 40 vox.[c]	NA
34	Venkatraman et al., 2007[247]	before-after	26 (12)	21.3±1.6 (NA)	t-fMRI (gambling task: decision making)	TAL	3	24h	p<0.001[c]	NA
	Venkatraman et al., 2007[247]	before-after	13 (3)	21.77±1.5 (NA)	t-fMRI (gambling task: reward processing without decision making)	TAL	6	24h	p<0.001[c]	NA
	Venkatraman et al., 2011[248]	before-after	29 (14)	22.34±1.23 (adult)	t-fMRI (decision making)	MNI	5	1 night	z>2.3 GRF[b]	NA
35	Wang L et al., 2016[249]	before-after	16 (8)	24.51±2.75 (NA)	rs-fMRI (ALFF)	MNI	5	24h	p<0.001, p<0.05, 351mm^3[b]	NA
36	Wu et al., 2006[250]	before-after	32 (17)	28.3±9.4 (19-47)	PET (glucose metabolism during visual vigilance task)	TAL	17	29-34h	p<0.05, p<0.05[b]	NA
37	Xu et al., 2016[251]	before-after	22 (9)	22.5±1.7 (18-35)	PET (glucose metabolism during mathematical processing task)	TAL	19	24h	p<0.05 FWE, 10 vox.[b]	NA
	Zhu et al., 2016[252 a]	before-after	28 (14)	22.1±1.6 (18-35)	rs-fMRI (VMHC)	TAL	10	1 night	p<0.05 FWE, 3 vox.[b]	mean FD
38	Ye et al., 2022[a253]	before-after	21 (15)	28.9±3.4 (18<)	rs-fMRI (gFCD, lrFCD)	MNI	2	1 night	p<0.001, p<0.05 GRF[b]	age, sex, education

© 2025 Reimann GM et al. *JAMA Psychiatry*.

	Ye et al., 2023[254 a]	before-after	22 (16)		29.0±3.7 (20-40)		VBM (GMD)	**MNI**	**14**	**1 night**	**p<0.001, p<0.01 GRF[b]**	**NA**
**Partial Sleep Deprivation**												
1	Alsameen et al., 2021[255 a]	before-after	36 (24)		15.7±0.8 (14-16.9)		t-fMRI (N-Back task)	MNI	6	6.5h x 5d	p<0.05 FWE[b]	gender, race (dichotomized for analysis as Caucasian vs. other), measured IQ
	DiFrancesco et al., 2023[256 a]	before-after	39 (22)		16.0±1.1 (14.1-17.9)		t-fMRI (visual food paradigm [food vs. non-food])	MNI	1	6.5h x 5d	p<0.005, p<0.05 FWE[b]	NA
2	Demos et al., 2017[257 a]	before-after	30 (22)		36.7±10.83 (21-55)		t-fMRI (food visual stimuli [food vs. object])	MNI	2	6h x 4d	p<0.001, p<0.05 FDR, 5 vox.[b]	NA
3	Li XY et al., 2023[258 a]	before-after	26 (10)		29.6±5.3 (20-40)		t-fMRI (visual food vs. non-food items)	MNI	2	6h12±26m in x 6 weeks	p<0.01, 11 vox.[c]	NA
4	Peters et al., 2014[259 a]	case-control	16 (17 [n=32])	16 (17 [n=32])	24.47±2.97 (21-30 [n=32])	24.38±2.52 (NA)	t-fMRI (fear conditioning / associative learning)	MNI	1	4h x 1d	p<0.01, p<0.05 FWE[b]	chronotype
5	Poudel et al., 2013[260 a]	before-after	20 (10)		24.9 (20-37)		t-fMRI (visuomotor tracking)	MNI	5	4h x 1d	p<0.05[b]	NA
6	Robinson et al., 2018[261 a]	before-after	18 (9)		14.40±1.94 (13-15)		rs-fMRI (ReHo)	MNI	10	4h x 1d	p<0.0001[c]	NA
7	St-Onge et al., 2012[262 a]	before-after	26 (12)		35.1±5.1 (30-45)		t-fMRI (food visual stimuli [food vs. object))	MNI	59	4h x 6d	p<0.01, 11 vox.[c]	NA
	St-Onge et al., 2014[263 a]	before-after	25 (12)		34.7±4.7 (30-45)		t-fMRI (food visual stimuli [healthy vs. unhealthy])	MNI	4	4h x 5d	p<0.05, 10 vox.[c]	NA

[a] Newly added experiments, that have not been included in any of our previous meta-analyses
[b] Corrected p-value and threshold
[c] Uncorrected p-value and threshold

© 2025 Reimann GM et al. *JAMA Psychiatry*.

Abbreviations: ALFF: amplitude of low-frequency fluctuations; dALFF: dynamic amplitude of low-frequency fluctuations; FD: framewise displacement; FDR: false discovery rate; FS: functional stability; FWE: family-wise error; gFCD: global functional connectivity density (functional connectivity strength); GMD / GMV: gray matter density/volume; GRF: Gaussian random field; group-SSR: group spatial sparse representation; ICA: independent component analysis; IQ: intelligence quotient; lrFCD: long-range functional connectivity density; MNI: Montreal Neurological Institute; NA: not available; Nr.: Number; PerAF: percent amplitude of fluctuation; PET: positron emission tomography; ReHo: regional homogeneity; rs-fMRI: resting-state functional magnetic resonance imaging; srFCD: short-range/local functional connectivity density; TAL: Talairach; TFCE: threshold-free cluster enhancement; t-fMRI: task-based functional magnetic resonance imaging; VBM: voxel-based morphometry; VMHC: voxel-mirrored homotopic connectivity; vox.: voxels

© 2025 Reimann GM et al. *JAMA Psychiatry*.

eTable 4. Number of Experiments for Each Meta-Analysis

	All	Decrease	Increase	rs-fMRI	t-fMRI	PET	Tasks	Functional	sMRI	Local	Adults	Higher Power	Corrected	22h-26h
Congenital Central (Alveolar) Hypoventilation Syndrome	1	1	1	0	1	0	1	1	0	1	0	0	1	x
Hypersomnia	2	0	2	0	0	2	0	2	0	2	2	0	2	x
Insomnia Disorder	28 (1[a])	20 (1)	24 (0)	15	9	1	10	25 (0)	8	24 (0)	28 (1[a])	19 (0)	23 (0)	x
Kleine-Levine Syndrome	1	1	1	0	1	0	1	1	0	1	1	0	0	x
Narcolepsy	15	13	10	4	3	3	3	10	6	15	10	5	11	x
Nightmare Disorder	1	1	1	1	0	0	0	1	0	1	1	0	1	x
Obstructive Sleep Apnea	28 (0)	25 (0)	18 (0)	10	4	3	4	17 (0)	15	27 (0)	25 (0)	14	23 (0)	x
Periodic Limb Movement Disorder	1	0	1	0	0	1	0	1	0	1	1	0	1	x
Rapid Eye Movement Behaviour Disorder	16	13	9	5	1	3	1	9	10	16	16	8	13	x
Restless Legs Syndrome	6	2	6	3	0		0	3	5	6	6	5	5	x
Sleepwalking	1	1	0	0	0	0	0	0	1	1	1	0	1	x
Partial Sleep Deprivation	7	3	6	1	6	0	6	7	0	7	5	4	4	x
Total Sleep Deprivation	38 (1)	30 (0)	29 (0)	11	27 (1[a])	3	30 (1)	38 (1)	4	38 (0)	37 (0)	20 (1)	29 (1)	20 (0)
**Sleep Disorders**	95 (2)	76 (1)	69 (1)	38 (1)	19 (0)	10	20 (0)	66 (1)	45 (1)	91 (2)	86 (1)	51 (1)	77 (0)	x
**Sleep Deprivation**	45 (1)	33 (0)	35 (1)	12	33 (1)	3	36 (1)	45 (1)	4	45 (2)	42 (2)	24 (1)	33 (1)	x

Decrease: analyses including any kind of experiment showing a decrease in activity, connectivity, metabolism, grey matter, or brain volume in the patient/experimental group; Increase: analyses including any kind of experiment showing an increase in activity, connectivity, metabolism, grey matter, or brain volume in the patient/experimental group; Local: analyses including only experiments of local voxel-wise measures; Corrected: analyses including only coordinates that are corrected for multiple comparisons; Higher Power: analyses including only experiments with a sample size of at least 21 participants, i.e. higher power; 22h-26h: analyses including only experiments performed after around 22h-26h of total sleep deprivation (time around the circadian nadir for alertness). Colours: Red: analysis was not performed due to non-sufficient number of experiments (<17); Yellow: analysis was performed (number of experiments ≥ 17) but yielded non-significant results; Green: analysis was performed and yielded at least one significant cluster. Analyses were only performed when at least 17 experiments were included. Threshold was set at p < 0.001 for the voxel level and p < 0.05 for cluster-level family-wise error correction.
[a] at least one significant cluster survived sub-analyses Bonferroni correction (p = 0.001 voxel-wise, p = 0.05 / 46 = 0.0011 cluster-level)

Abbreviations: PET: positron emission tomography; rs-fMRI: resting-state functional magnetic resonance tomography; sMRI: structural magnetic resonance tomograph; t-fMRI: task-based functional magnetic resonance tomography

© 2025 Reimann GM et al. *JAMA Psychiatry*.

eTable 5. Convergent Regional Alterations Based on Patient/Experimental Group

Region	Cluster Size	Peak Z	MNI Coordinates			Nr. of Foci	Nr. of Experiments in Analysis	Nr. of Contributing Experiments	Macroanatomy	Cytoarchitecture
			X	Y	Z					
**Sleep Disorders**										
Bilateral Subgenual Anterior Cingulate Cortex (sgACC)	176	4.86	2	34	-20	1417	95	17	Frontal Medial Cortex (62.3%), Paracingulate Gyrus (11.4%)	Area s32 (48.7%), Area s24 (11.2%), Area Fo1 (4.2%)
Right Amygdala/ Hippocampus (rAmyg/rHipp)	130	4.00	18	-2	-18	1417	95	25	Right Amygdala (47.2%), Right Hippocampus (47.1%)	Amygdala (LB) (210%), Amygdala (SF) (17.1%), CA1 (Hippocampus (16.2%), Amygdala (VTM) (11.2%), DG (Hippocampus) (9.3%)
**Sleep Deprivation**										
Right Thalamus (rThal)	153	5.21	10	-18	6	962	45	13	Right Thalamus (100%)	
**Insomnia Disorder**										
Bilateral Subgenual Anterior Cingulate Cortex (sgACC)[a]	158	4.25	2	34	-14	296	28	7	Frontal Medial Cortex (55.0%), Paracingulate Gyrus (20.4%)	Area s32 (48.1%), Area s24 (10.8%), Area Fo1 (10.7%)
**Total Sleep Deprivation**										
Right Thalamus (rThal)	102	4.96	10	-18	8	872	38	11	Right Thalamus (100%)	

[a] Survived Bonferroni-correction for sub-analyses ($p = 0.001$ voxel-wise, $p = 0.05 / 46 = 0.0011$ cluster-level)
Macroanatomy and cytoarchitecture based on maximum probability maps of SPM Anatomy Toolbox v3.0.

Abbreviations: CA1: cornu ammonis; DG: dentate gyrus; Fo1: frontal gyrus orbital part one; LB: laterobasal group; rAmyg/rHipp: right amygdala/hippocampus; rThal: right thalamus; s24/32: subgenual area 24/32; sgACC: bilateral subgenual anterior cingulate cortex; SF: superficial group; VTM: ventromedial part

© 2025 Reimann GM et al. *JAMA Psychiatry*.

eTable 6. Convergent Regional Alterations Based on “Control > Patient/Experimental” (Decrease)

Region	Cluster Size	Peak Z	MNI Coordinates			Nr. of Foci	Nr. of Experiments in Analysis	Nr. of Contributing Experiments	Macroanatomy	Cytoarchitecture
			X	Y	Z					
**Sleep Disorders**										
Bilateral Subgenual Anterior Cingulate Cortex (sgACC)	122	4.62	2	34	-20	802	76	12	Frontal Medial Cortex (48.3%), Subcallosal Cortex (17.4%)	Area s32 (25.7%), Area Fo1 (21.8%), Area Fo2 (9.0%)
**Insomnia Disorder**										
Right Inferior & Middle Frontal Gyrus (rIFG/rMFG)	97	4.46	50	18	28	173	20	6	Inferior Frontal Gyrus, pars opercularis (67.4%), Middle Frontal Gyrus (32.5%)	Area 45 (36.7%), Area 44 (20.4%)

Macroanatomy and cytoarchitecture based on SPM Anatomy Toolbox v3.0 (https://www.fz-juelich.de/en/inm/inm-7/resources/jubrain-anatomy-toolbox).

Abbreviations: Fo1/2: frontal gyrus orbital part one/two; rIFG/rMFG: right inferior & middle frontal gyrus; sgACC: subgenual anterior cingulate cortex

© 2025 Reimann GM et al. *JAMA Psychiatry*.

eTable 7. Convergent Regional Alterations Based on "Patient/Experimental > Control" (Increase)

Region	Cluster Size	Peak Z	MNI Coordinates			Nr. of Foci	Nr. of Experiments in Analysis	Nr. of Contributing Experiments	Macroanatomy	Cytoarchitecture
			X	Y	Z					
**Sleep Disorders**										
Right Amygdala/ Hippocampus (rAmyg/rHipp)	137	4.53	28	-14	-16	614	69	15	Right Hippocampus (61.7%), Right Amygdala (6.1%)	CA1 (Hippocampus (33.6%), Amygdala (VTM) (12.0%), DG (Hippocampus) (6.8%)
**Sleep Deprivation**										
Right Thalamus (rThal)	127	4.42	8	-14	6	458	35	7	Right Thalamus (99.9%)	

Macroanatomy and cytoarchitecture based on SPM Anatomy Toolbox v3.0 (https://www.fz-juelich.de/en/inm/inm-7/resources/jubrain-anatomy-toolbox).

Abbreviations: CA1: cornu ammonis; DG: dentate gyrus; rAmyg/rHipp: right amygdala/hippocampus; rThal: right thalamus; VTM: ventromedial part

© 2025 Reimann GM et al. *JAMA Psychiatry*.

eTable 8. Convergent Regional Alterations Based on rs-fMRI

Region	Cluster Size	Peak Z	MNI Coordinates			Nr. of Foci	Nr. of Experiments in Analysis	Nr. of Contributing Experiments	Macroanatomy	Cytoarchitecture
			X	Y	Z					
**Sleep Disorders**										
Left Insula/Putamen (lIns/lPut)	95	4.02	-36	2	-2	494	38	8	Insular Cortex (19.7%), Left Putamen (13.7%)	

Macroanatomy and cytoarchitecture based on SPM Anatomy Toolbox v3.0 (https://www.fz-juelich.de/en/inm/inm-7/resources/jubrain-anatomy-toolbox).

Abbreviations: lIns/lPut: left insula/putamen

© 2025 Reimann GM et al. *JAMA Psychiatry*.

eTable 9. Convergent Regional Alterations Based on t-fMRI

Region	Cluster Size	Peak Z	MNI Coordinates			Nr. of Foci	Nr. of Experiments in Analysis	Nr. of Contributing Experiments	Macroanatomy	Cytoarchitecture
			X	Y	Z					
**Sleep Deprivation**										
Right Insula/Frontal Operculum (rIns/rFO)	165	4.43	46	18	-8	653	33	13	Insular Cortex (50.8%), Frontal Operculum Cortex (20.0%)	Area OP8 (10.7%), Area Id7 (9.7%)
**Total Sleep Deprivation**										
Right Insula/Frontal Operculum (rIns/rFO)[a]	163	4.50	46	18	-8	573	27	11	Insular Cortex (48.1%), Frontal Operculum Cortex (16.8%)	Area Id7 (12.1%), Area OP8 (8.7%)

[a] Survived Bonferroni correction for sub-analyses (p = 0.001 voxel-wise, p = 0.05 / 46 = 0.0011 cluster-level)
Macroanatomy and cytoarchitecture based on SPM Anatomy Toolbox v3.0 (https://www.fz-juelich.de/en/inm/inm-7/resources/jubrain-anatomy-toolbox).

Abbreviations: rIns/rFO: right insula/frontal operculum; OP8: frontal operculum area 8

© 2025 Reimann GM et al. *JAMA Psychiatry*.

eTable 10. Convergent Regional Alterations Based on Tasks

Region	Cluster Size	Peak Z	MNI Coordinates			Nr. of Foci	Nr. of Experiments in Analysis	Nr. of Contributing Experiments	Macroanatomy	Cytoarchitecture
			X	Y	Z					
**Sleep Deprivation**										
Right Insula/Frontal Operculum (rIns/rFO)	129	4.29	46	18	-8	742	36	11	Insular Cortex (50.8%), Frontal Operculum Cortex (20.0%)	Area OP8 (10.7%), Area Id7 (9.7%)
**Total Sleep Deprivation**										
Right Insula/Frontal Operculum (rIns/rFO)	136	4.35	46	18	-8	662	30	11	Insular Cortex (48.1%), Frontal Operculum Cortex (16.8%)	Area Id7 (12.1%), Area OP8 (8.7%)

Macroanatomy and cytoarchitecture based on SPM Anatomy Toolbox v3.0 (https://www.fz-juelich.de/en/inm/inm-7/resources/jubrain-anatomy-toolbox).

Abbreviations: lIns/lPut: left insula/putamen; OP8: frontal operculum area 8

© 2025 Reimann GM et al. *JAMA Psychiatry*.

eTable 11. Convergent Regional Alterations Based on Functional

Region	Cluster Size	Peak Z	MNI Coordinates			Nr. of Foci	Nr. of Experiments in Analysis	Nr. of Contributing Experiments	Macroanatomy	Cytoarchitecture
			X	Y	Z					
**Sleep Disorders**										
Left Insula/Putamen (lIns/lPut)	118	4.79	-36	2	-4	1026	66	14	Insular Cortex (19.7%), Left Putamen (13.7%)	
**Sleep Deprivation**										
Right Thalamus (rThal)	150	5.18	10	-18	6	926	45	11	Right Thalamus (99.9%)	
**Total Sleep Deprivation**										
Right Thalamus (rThal)	100	4.82	10	-18	6	836	38	9	Right Thalamus (99.9%)	

Macroanatomy and cytoarchitecture based on SPM Anatomy Toolbox v3.0 (https://www.fz-juelich.de/en/inm/inm-7/resources/jubrain-anatomy-toolbox).

Abbreviations: rIns/rFO: right insula/frontal operculum; rThal: right thalamus

© 2025 Reimann GM et al. *JAMA Psychiatry*.

eTable 12. Convergent Regional Alterations Based on sMRI

Region	Cluster Size	Peak Z	MNI Coordinates			Nr. of Foci	Nr. of Experiments in Analysis	Nr. of Contributing Experiments	Macroanatomy	Cytoarchitecture
			X	Y	Z					
**Sleep Disorders**										
Left Thalamus (lThal)	94	5.32	-16	-24	12	391	45	6	Left Thalamus (100%)	

Macroanatomy and cytoarchitecture based on SPM Anatomy Toolbox v3.0 (https://www.fz-juelich.de/en/inm/inm-7/resources/jubrain-anatomy-toolbox).

Abbreviations: lThal: left thalamus

© 2025 Reimann GM et al. *JAMA Psychiatry*.

eTable 13. Convergent Regional Alterations Based on Local Measures

Region	Cluster Size	Peak Z	MNI Coordinates			Nr. of Foci	Nr. of Experiments in Analysis	Nr. of Contributing Experiments	Macroanatomy	Cytoarchitecture
			X	Y	Z					
**Sleep Disorders**										
Right Amygdala/ Hippocampus (rAmyg/rHipp)	186	4.18	18	-2	-18	1227	91	23	Right Amygdala (45.9%) Right Hippocampus (45.3%),	Amygdala (LB) (21.0%), Amygdala (SF) (16.0%), CA1 (Hippocampus (15.0%), Amygdala (VTM) (12.5%), DG (Hippocampus) (9.0%)
Bilateral Subgenual Anterior Cingulate Cortex (sgACC)	161	4.98	2	34	-20	1227	91	16	Frontal Medial Cortex (65.5%), Subcallosal Cortex (9.3%)	Area s32 (37.9%), Area Fo1 (19.1%), Area Fo2 (2.6%)
**Sleep Deprivation**										
Right Thalamus (rThal)	122	5.25	10	-18	6	914	45	13	Right Thalamus (100%)	
Right Insula/Frontal Operculum (rIns/rFO)	105	3.97	46	18	-8	914	45	13	Insular Cortex (46.7%), Frontal Operculum Cortex (26.4%)	Area OP8 (8.9%), Area Id7 (7.1%)

Macroanatomy and cytoarchitecture based on SPM Anatomy Toolbox v3.0 (https://www.fz-juelich.de/en/inm/inm-7/resources/jubrain-anatomy-toolbox).

Abbreviations: CA1: cornu ammonis; DG: dentate gyrus; Fo1/2: frontal gyrus orbital part one/two; LB: laterobasal group; rAmyg/rHipp: right amygdala/hippocampus; rIns/rFO: right insula/frontal operculum; OP8: frontal operculum area 8; rThal: right thalamus; s32: subgenual area 32; SF: superficial group; sgACC: subgenual anterior cingulate cortex; VTM: ventromedial part

© 2025 Reimann GM et al. *JAMA Psychiatry*.

eTable 14. Convergent Regional Alterations Based on Adults-Only

Region	Cluster Size	Peak Z	MNI Coordinates			Nr. of Foci	Nr. of Experiments in Analysis	Nr. of Contributing Experiments	Macroanatomy	Cytoarchitecture
			X	Y	Z					
**Sleep Disorders**										
Bilateral Subgenual Anterior Cingulate Cortex (sgACC)	184	4.61	2	34	-20	1217	86	16	Frontal Medial Cortex (63.9 %), Paracingulate Gyrus (13.2%)	Area s32 (47.0%), Area Fo1 (11.8%), Area s24 (5.3%), Area Fo2 (3.5%)
**Sleep Deprivation**										
Right Thalamus (rThal)	248	5.19	10	-18	8	909	42	12	Right Thalamus (99.9%)	
Right Insula/Frontal Operculum (rIns/rFO)	105	3.97	46	18	-8	909	42	12	Insular Cortex (46.7%), Frontal Operculum Cortex (26.4%)	Area OP8 (8.9%), Area Id7 (7.1%)
**Insomnia Disorder**										
Bilateral Subgenual Anterior Cingulate Cortex (sgACC)[a]	158	4.25	2	34	-14	296	28	7	Frontal Medial Cortex (55.0%), Paracingulate Gyrus (20.4%)	Area s32 (48.1%), Area s24 (10.8%), Area Fo1 (10.7%)

[a] Survived Bonferroni correction for sub-analyses (p = 0.001 voxel-wise, p = 0.05 / 46 = 0.0011 cluster-level)
Macroanatomy and cytoarchitecture based on SPM Anatomy Toolbox v3.0 (https://www.fz-juelich.de/en/inm/inm-7/resources/jubrain-anatomy-toolbox).

Abbreviations: Fo1/2: frontal gyrus orbital part one/two; rIns/rFO: right insula/frontal operculum; OP8: frontal operculum area 8; rThal: right thalamus; s24/32: subgenual area 24/32; sgACC: subgenual anterior cingulate cortex

© 2025 Reimann GM et al. *JAMA Psychiatry*.

eTable 15. Convergent Regional Alterations Based on Higher Power Experiments

Region	Cluster Size	Peak Z	MNI Coordinates			Nr. of Foci	Nr. of Experiments in Analysis	Nr. of Contributing Experiments	Macroanatomy	Cytoarchitecture
			X	Y	Z					
**Sleep Disorders**										
Bilateral Subgenual Anterior Cingulate Cortex (sgACC)	131	4.32	2	34	-20	684	51	11	Frontal Medial Cortex (55.2 %), Subcallosal Cortex (14.3 %)	Area s32 (46.0%), Area Fo1 (11.6%), Area s24 (6.2%)
**Sleep Deprivation**										
Right Thalamus (rThal)	119	4.18	8	-10	4	396	24	7	Right Thalamus (99.7%)	
**Total Sleep Deprivation**										
Right Thalamus (rThal)	90	4.07	10	-18	6	322	20	6	Right Thalamus (100%)	

Macroanatomy and cytoarchitecture based on SPM Anatomy Toolbox v3.0 (https://www.fz-juelich.de/en/inm/inm-7/resources/jubrain-anatomy-toolbox).

Abbreviations: Fo1: frontal gyrus orbital part one/two; rThal: right thalamus; s24/32: subgenual area 24/32; sgACC: subgenual anterior cingulate cortex

© 2025 Reimann GM et al. *JAMA Psychiatry*.

eTable 16. Convergent Regional Alterations Based on Corrected Coordinates

Region	Cluster Size	Peak Z	MNI Coordinates			Nr. of Foci	Nr. of Experiments in Analysis	Nr. of Contributing Experiments	Macroanatomy	Cytoarchitecture
			X	Y	Z					
**Sleep Deprivation**										
Right Thalamus (rThal)	169	5.75	10	-18	8	581	33	10	Right Thalamus (99.9%)	
**Total Sleep Deprivation**										
Right Thalamus (rThal)	248	5.34	10	-18	8	566	29	9	Right Thalamus (100%)	

Macroanatomy and cytoarchitecture based on SPM Anatomy Toolbox v3.0 (https://www.fz-juelich.de/en/inm/inm-7/resources/jubrain-anatomy-toolbox).

Abbreviations: rThal: right thalamus

© 2025 Reimann GM et al. *JAMA Psychiatry*.

eFigure 1. Significant Behavioural Decoding Domain Subcategories for the Meta-Analysis Across All Sleep Disorders

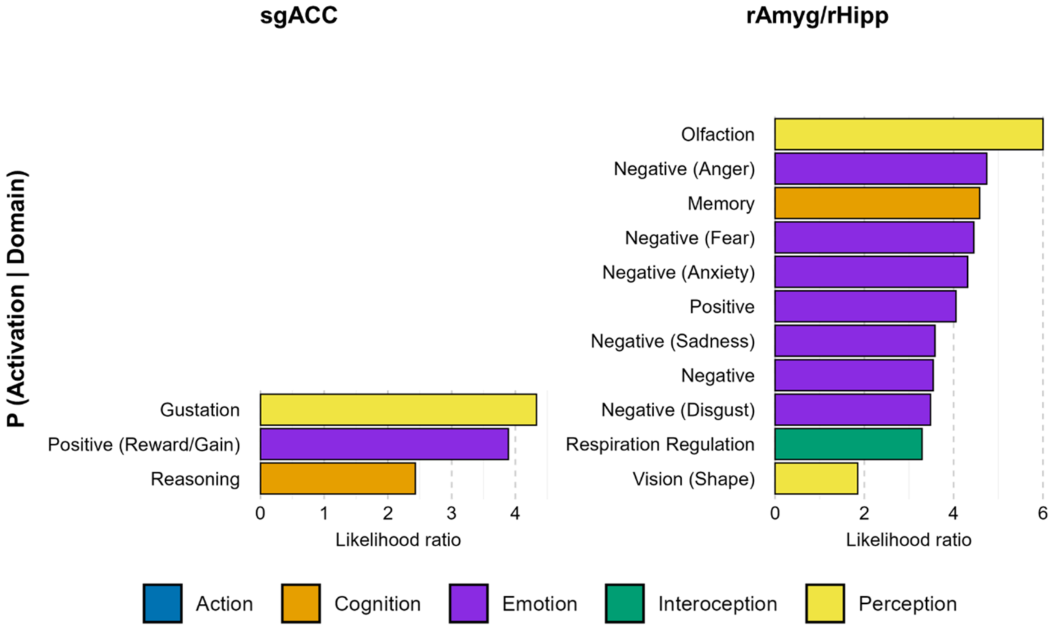

Threshold set to false discovery rate-corrected p-value < 0.05.

Abbreviations: rAmyg/rHipp: right amygdala/hippocampus; sgACC: subgenual anterior cingulate cortex

© 2025 Reimann GM et al. *JAMA Psychiatry*.

eFigure 2. Task-Based and Task-Free Connectivity for the Bilateral Subgenual Anterior Cingulate Cortex Cluster

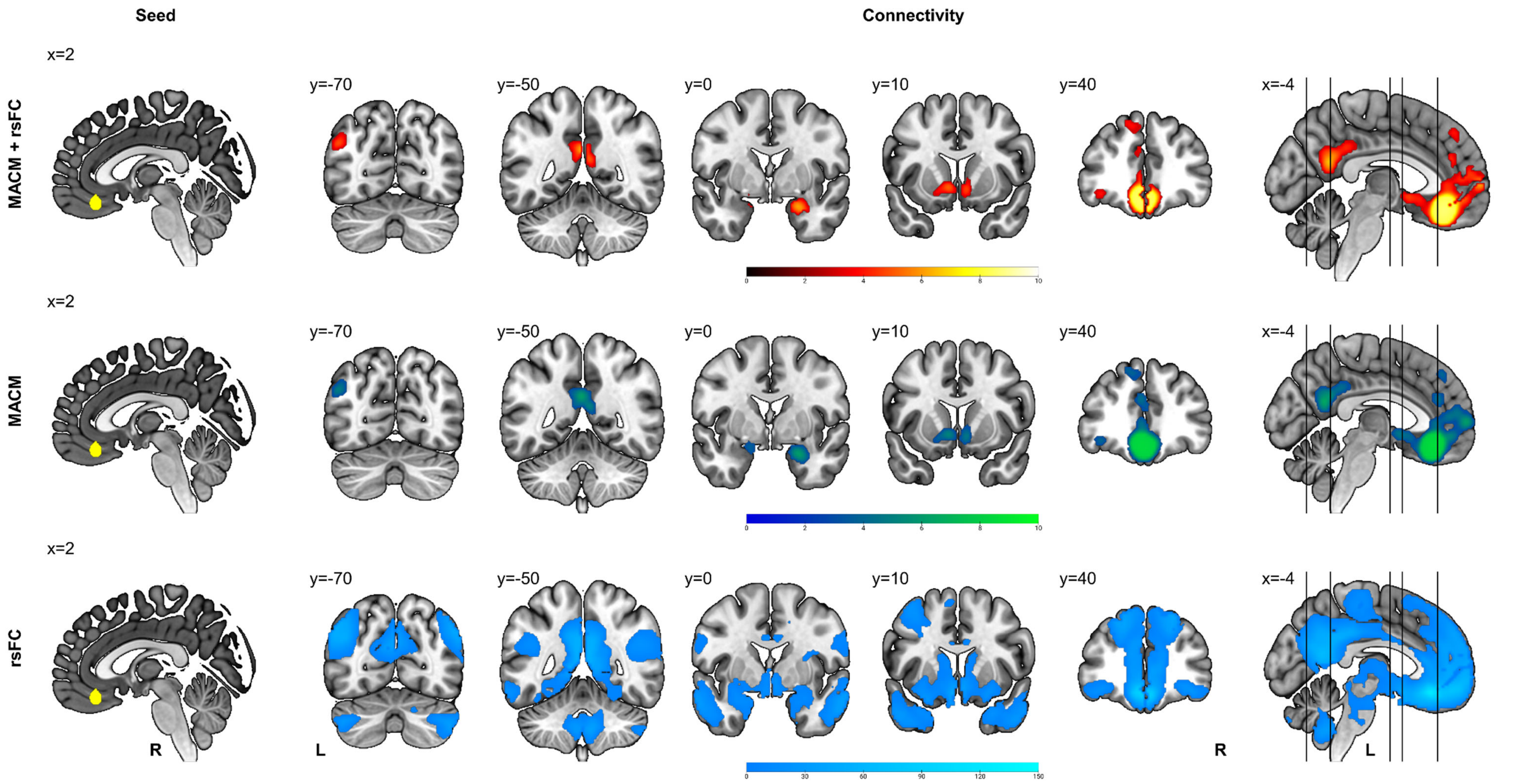

The task-based and task-free connectivity results of the bilateral subgenual anterior cingulate cortex cluster derived from the activation likelihood estimation across all sleep disorders. The top shows the conjunctive connectivity map of meta-analytic connectivity modelling (MACM) and resting-state functional connectivity (rsFC). Coordinates are provided in MNI space. Thresholds for MACM and rsFC were set at $p < 0.001$ for the voxel level and $p < 0.05$ for cluster-level family-wise error correction.

Abbreviations: MACM: meta-analytic connectivity modelling; rsFC: resting-state functional connectivity

© 2025 Reimann GM et al. *JAMA Psychiatry*.

eFigure 3. Task-Based and Task-Free Connectivity for the Right Amygdala/Hippocampus Cluster

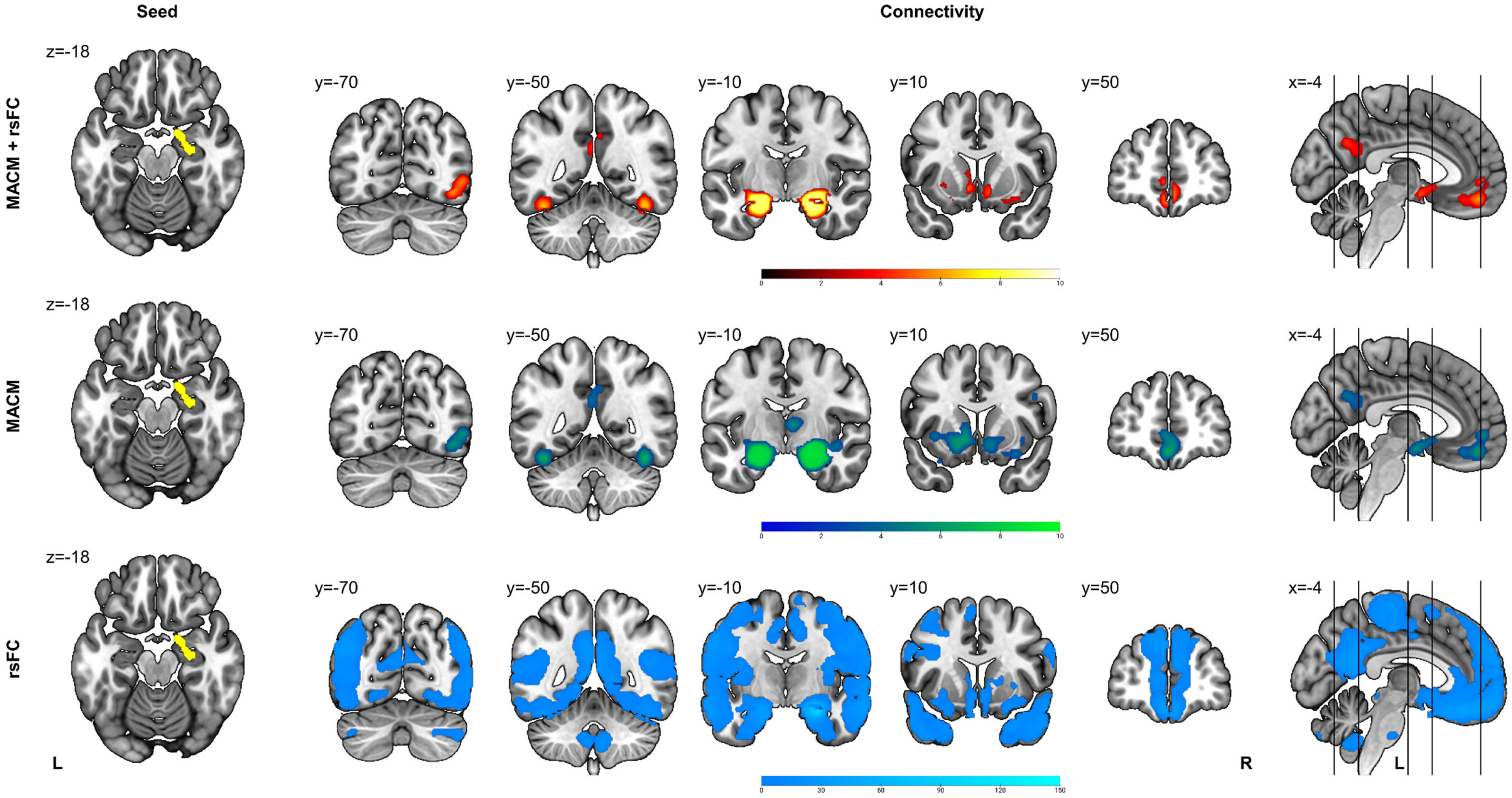

The task-based and task-free connectivity results of the right amygdala/hippocampus cluster derived from the activation likelihood estimation across all sleep disorders. The top shows the conjunctive connectivity map of meta-analytic connectivity modelling (MACM) and resting-state functional connectivity (rsFC). Coordinates are provided in MNI space. Thresholds for MACM and rsFC were set at $p < 0.001$ for the voxel level and $p < 0.05$ for cluster-level family-wise error correction.

Abbreviations: MACM: meta-analytic connectivity modelling; rsFC: resting-state functional connectivity

© 2025 Reimann GM et al. *JAMA Psychiatry*.

eFigure 4. Behavioural Decoding of Clusters From Sleep Deprivation Meta-Analysis

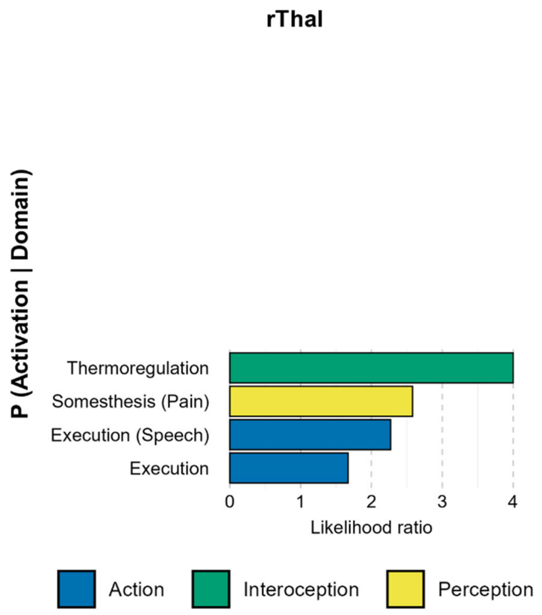

Threshold set to false discovery rate-corrected p-value < 0.05.

Abbreviations: rThal: right thalamus

© 2025 Reimann GM et al. *JAMA Psychiatry*.

eFigure 5. Task-Based and Task-Free Connectivity for the Right Thalamus Cluster

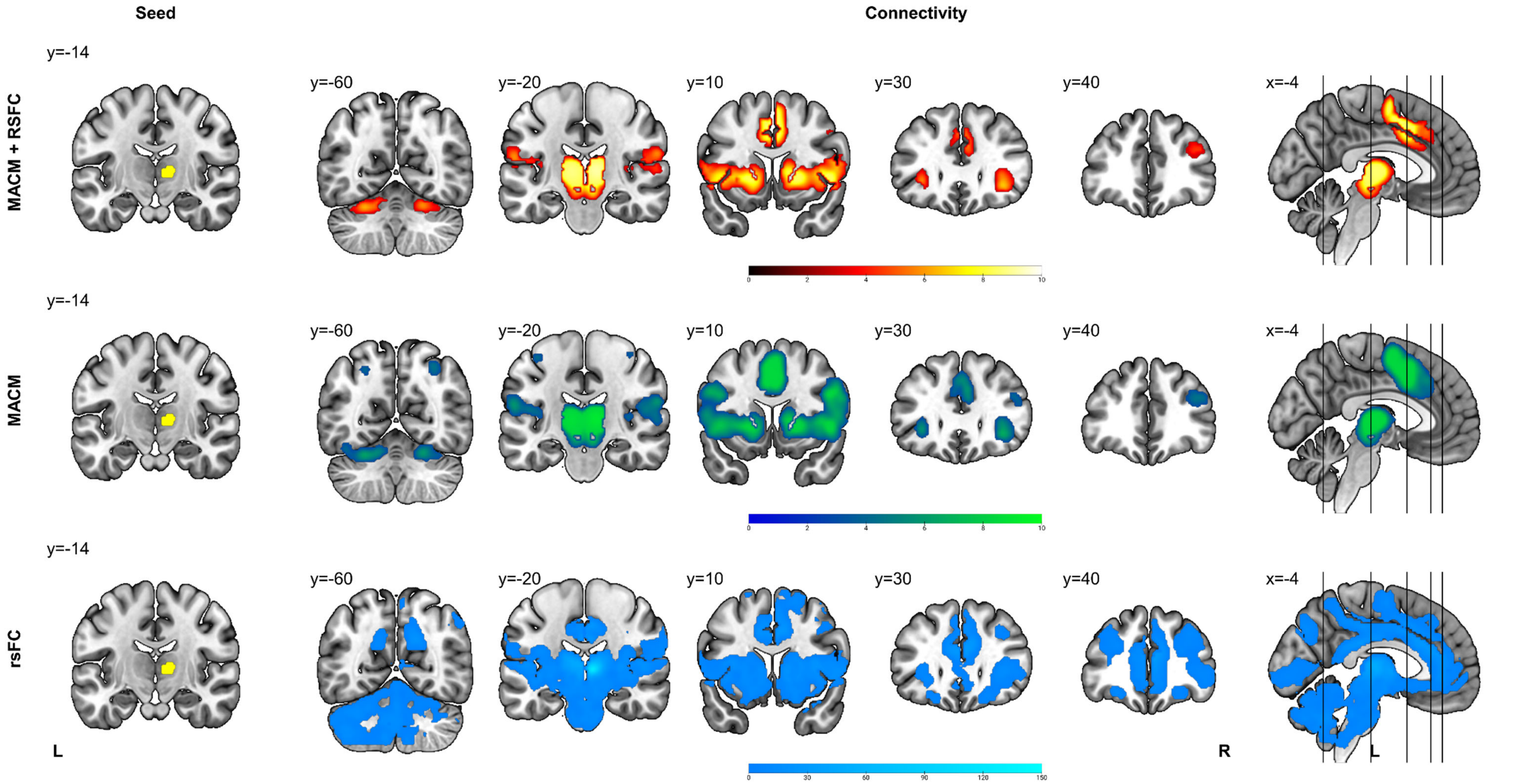

The task-based and task-free connectivity results of the right thalamus cluster derived from the activation likelihood estimation across sleep deprivation. The top shows the conjunctive connectivity map meta-analytic connectivity modelling (MACM) and resting-state functional connectivity (rsFC). Coordinates are provided in MNI space. Thresholds for MACM and rsFC were set at $p < 0.001$ for the voxel level and $p < 0.05$ for cluster-level family-wise error correction.

Abbreviations: MACM: meta-analytic connectivity modelling; rsFC: resting-state functional connectivity

© 2025 Reimann GM et al. *JAMA Psychiatry*.

eFigure 6. Shared Connectivity Maps of Clusters From Meta-Analyses Across Sleep Disorders and Sleep Deprivation

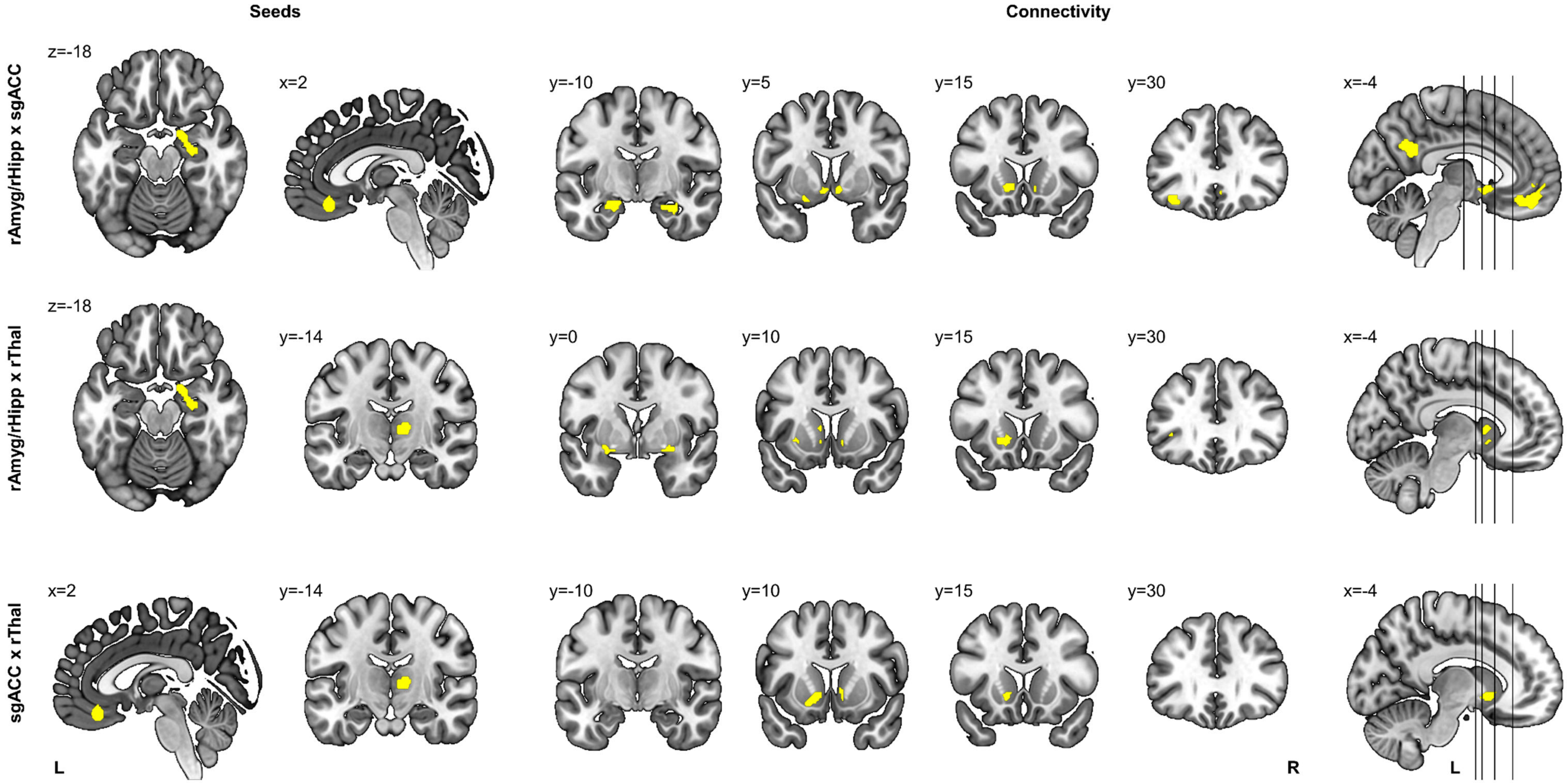

The clusters are: 1) right amygdala/hippocampus (rAmyg/rHipp), 2) bilateral subgenual anterior cingulate cortex (sgACC), and 3) right thalamus (rThal). Each row shows the shared connectivity map based on two clusters. The coordinates are in MNI space. Coordinates are provided in MNI space. Thresholds for MACM and rsFC were set at $p < 0.001$ for the voxel level and $p < 0.05$ for cluster-level family-wise error correction.

Abbreviations: MACM: meta-analytic connectivity modelling; rAmyg/rHipp: right amygdala/hippocampus; rsFC: resting-state functional connectivity; rThal: right thalamus; sgACC: subgenual anterior cingulate cortex

© 2025 Reimann GM et al. *JAMA Psychiatry*.

eFigure 7. Convergent Regional Alterations Based on Patient/Experimental Group

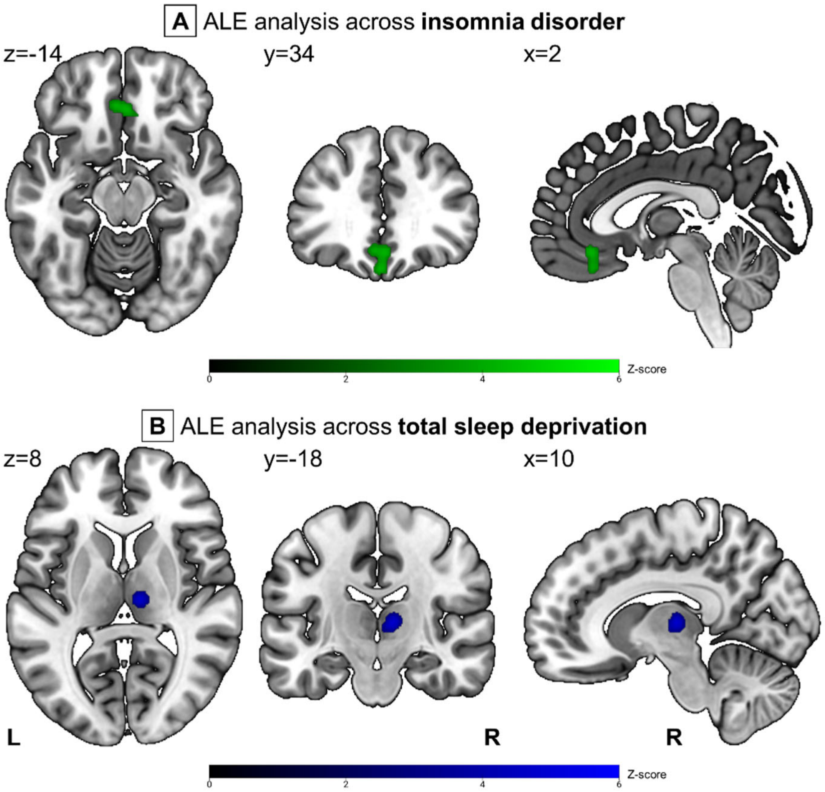

Activation likelihood estimation (ALE) results for each patient/experimental group; **A.** Across insomnia disorder identified one cluster, lying in the subgenual anterior cingulate cortex; **B.** Across sleep deprivation identified one cluster, lying in the right thalamus. Coordinates are provided in MNI space. Threshold was set at p < 0.001 for the voxel level and p < 0.05 for cluster-level family-wise error correction.

Abbreviations: ALE: activation likelihood estimation

© 2025 Reimann GM et al. *JAMA Psychiatry*.

eFigure 8. Convergent Regional Alterations Based on "Control > Patient/Experimental" (Decrease)

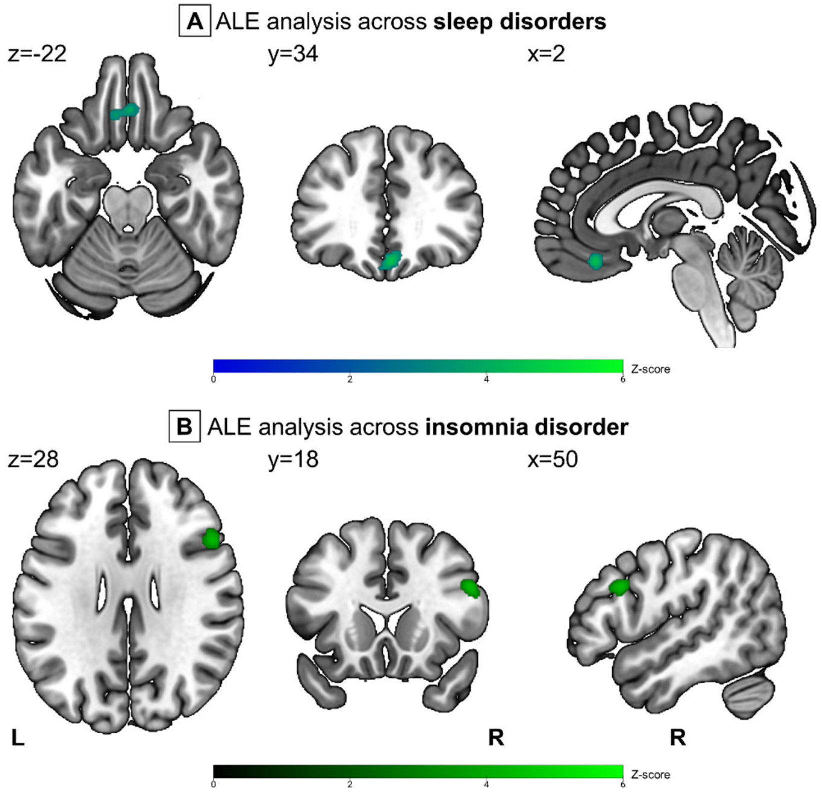

Activation likelihood estimation (ALE) results for experiments showing a decrease („control > patient/experimental"); **A.** Across all sleep disorders identified one cluster, lying in the subgenual anterior cingulate cortex; **B.** Across insomnia disorder identified one cluster, lying in the right inferior & middle frontal gyrus. Coordinates are provided in MNI space. Threshold was set at p < 0.001 for the voxel level and p < 0.05 for cluster-level family-wise error correction.

Abbreviations: ALE: activation likelihood estimation

© 2025 Reimann GM et al. *JAMA Psychiatry*.

eFigure 9. Convergent Regional Alterations Based on “Patient/Experimental > Control” (Increase)

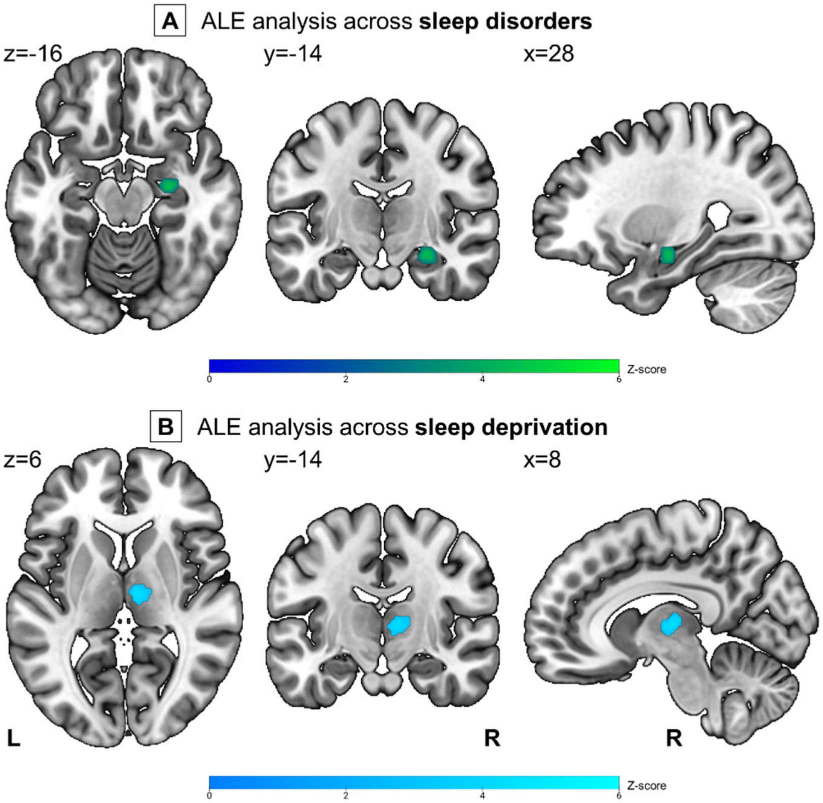

Activation likelihood estimation (ALE) results for experiments showing an increase („patient/experimental > control”); **A.** Across all sleep disorders identified one cluster, lying in the right amygdala/hippocampus; **B.** Across sleep deprivation identified one cluster, lying in the right thalamus. Coordinates are provided in MNI space. Threshold was set at p < 0.001 for the voxel level and p < 0.05 for cluster-level family-wise error correction.

Abbreviations: ALE: activation likelihood estimation

© 2025 Reimann GM et al. *JAMA Psychiatry*.

eFigure 10. Convergent Regional Alterations Based on rs-fMRI

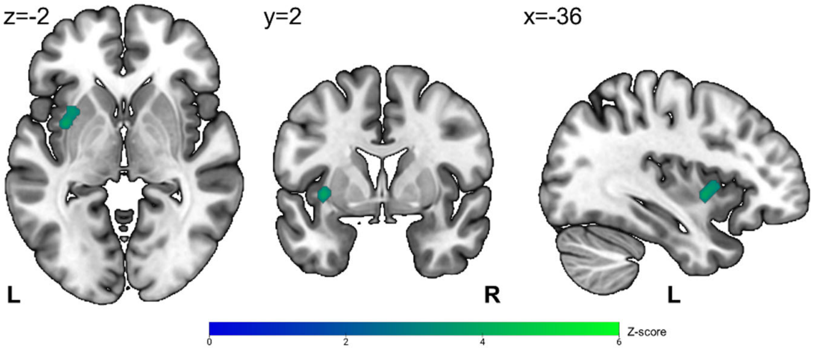

Activation likelihood estimation (ALE) results for rs-fMRI experiments across all sleep disorders identified one cluster, lying in the left insula/putamen. Coordinates are provided in MNI space. Threshold was set at $p < 0.001$ for the voxel level and $p < 0.05$ for cluster-level family-wise error correction.

Abbreviations: ALE: activation likelihood estimation

© 2025 Reimann GM et al. *JAMA Psychiatry*.

eFigure 11. Convergent Regional Alterations Based on t-fMRI

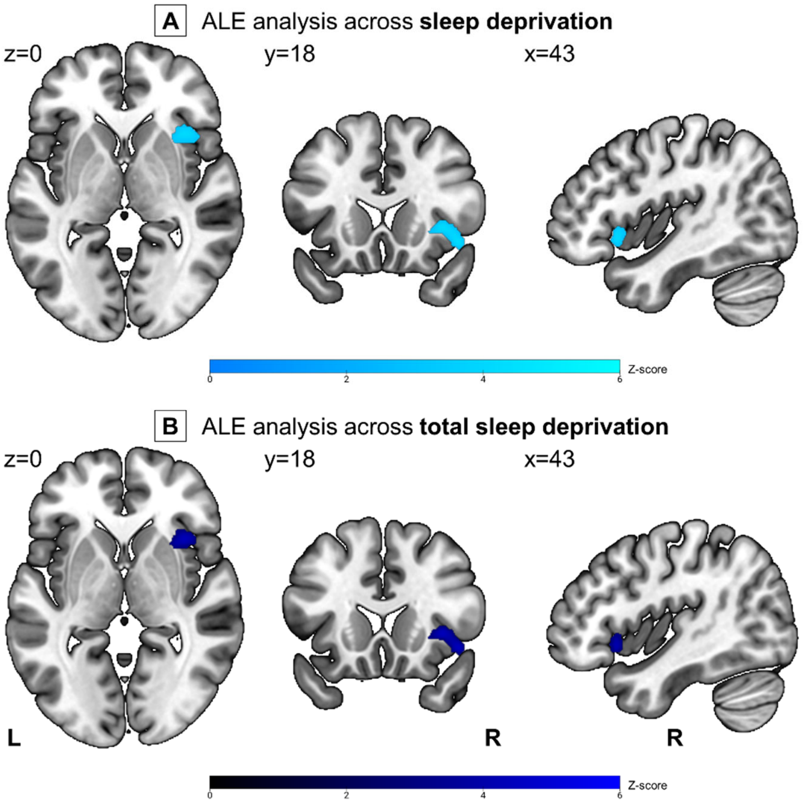

Activation likelihood estimation (ALE) results for t-fMRI experiments; **A.** Across sleep deprivation identified one cluster, lying in the right insula/frontal operculum; **B.** Across total sleep deprivation identified one cluster, lying in the right insula/frontal operculum. Coordinates are provided in MNI space. Threshold was set at $p < 0.001$ for the voxel level and $p < 0.05$ for cluster-level family-wise error correction.

Abbreviations: ALE: activation likelihood estimation

© 2025 Reimann GM et al. *JAMA Psychiatry*.

eFigure 12. Convergent Regional Alterations Based on Tasks

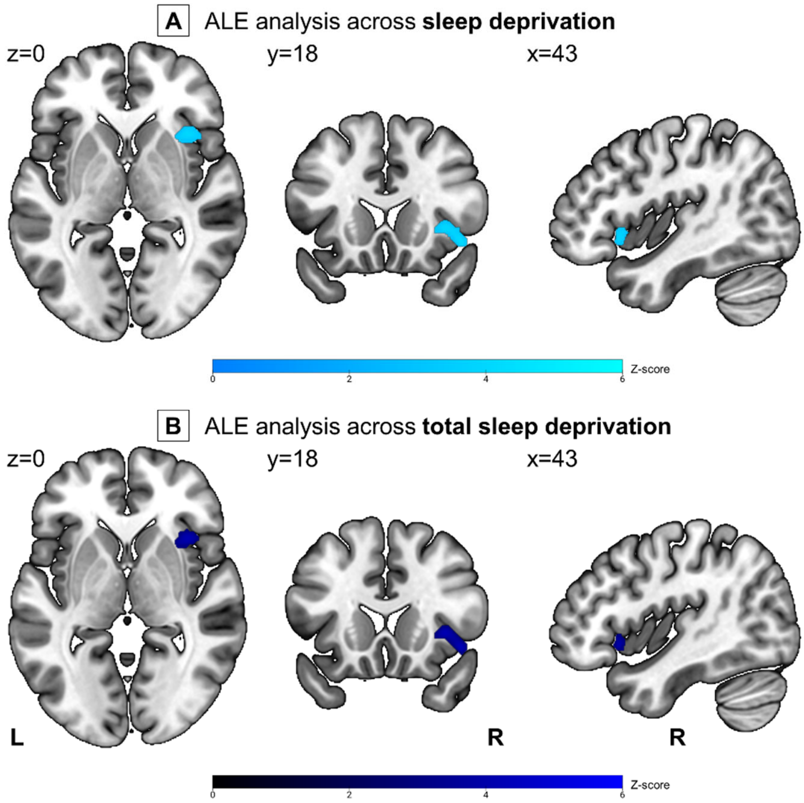

Activation likelihood estimation (ALE) results for experiments in which tasks are performed (t-fMRI, t-PET); **A.** Across sleep deprivation identified one cluster, lying in the right insula/frontal operculum; **B.** Across total sleep deprivation identified one cluster, lying in the right insula/frontal operculum. Coordinates are provided in MNI space. Threshold was set at $p < 0.001$ for the voxel level and $p < 0.05$ for cluster-level family-wise error correction.

Abbreviations: ALE: activation likelihood estimation

© 2025 Reimann GM et al. *JAMA Psychiatry*.

eFigure 13. Convergent Regional Alterations Based on Functional

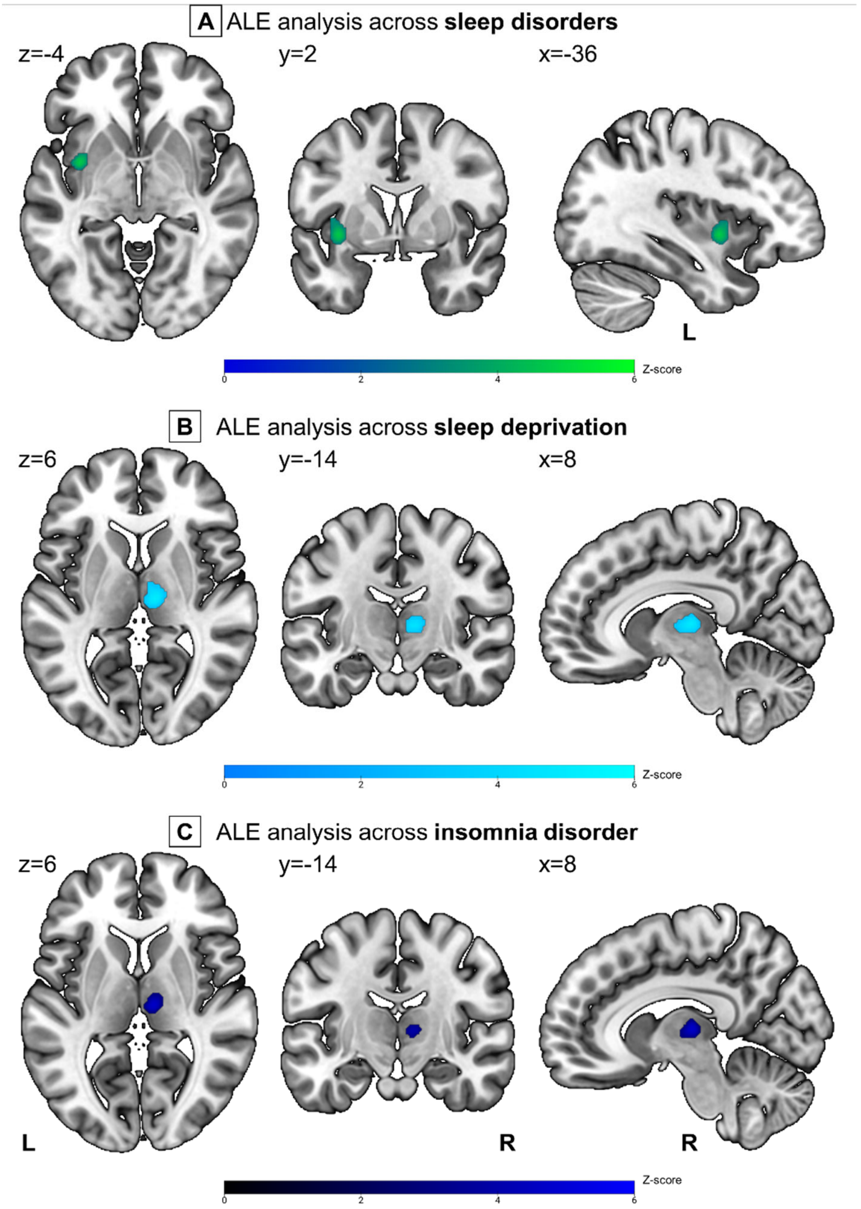

Activation likelihood estimation (ALE) results for functional experiments (i.e. rs.fMRI, t-fMRI, PET); **A.** Across all sleep disorders identified one cluster, lying in the left insula/putamen; **B.** Across sleep deprivation identified one cluster, lying in the right thalamus; **C.** Across total sleep deprivation identified one cluster, lying in the right thalamus. Coordinates are provided in MNI space. Threshold was set at $p < 0.001$ for the voxel level and $p < 0.05$ for cluster-level family-wise error correction.

Abbreviations: ALE: activation likelihood estimation

© 2025 Reimann GM et al. *JAMA Psychiatry*.

eFigure 14. Convergent Regional Alterations Based on sMRI

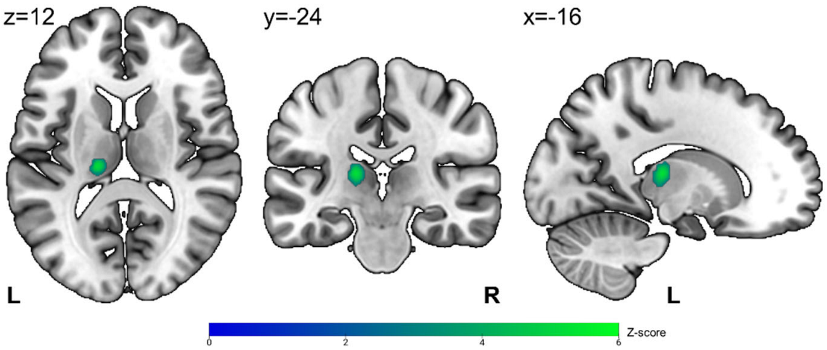

Activation likelihood estimation (ALE) results for sMRI experiments across all sleep disorders identified one cluster, lying in the left thalamus. Coordinates are provided in MNI space. Threshold was set at $p < 0.001$ for the voxel level and $p < 0.05$ for cluster-level family-wise error correction..

Abbreviations: ALE: activation likelihood estimation

© 2025 Reimann GM et al. *JAMA Psychiatry*.

eFigure 15. Convergent Regional Alterations Based on Local Measures

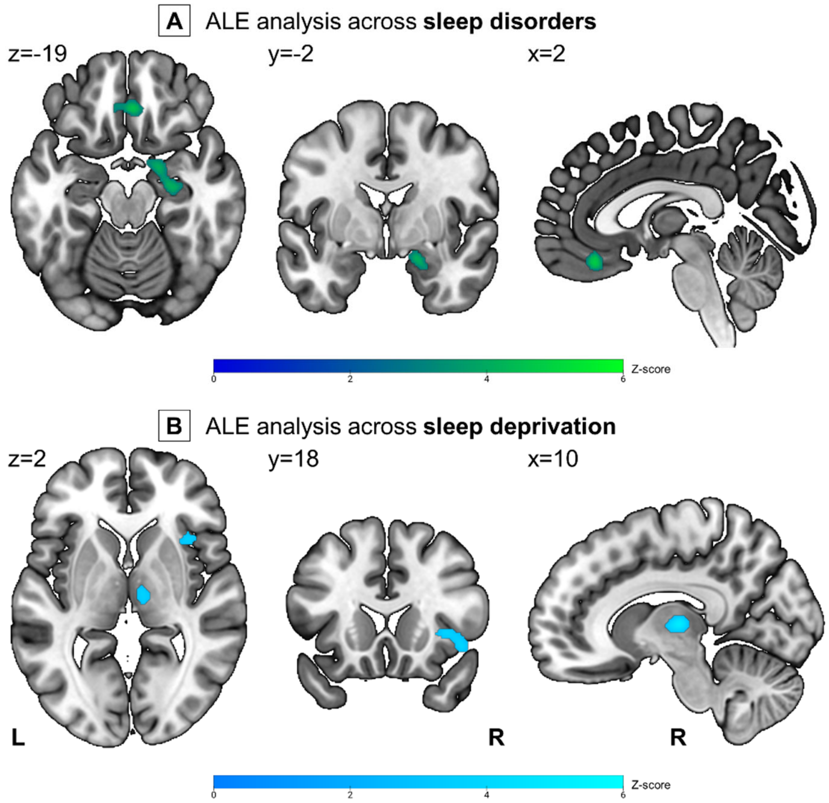

Activation likelihood estimation (ALE) results for local measure experiments; **A.** Across all sleep disorders identified two clusters, lying in the right amygdala/hippocampus and subgenual anterior cingulate cortex; **B.** Across sleep deprivation identified one cluster, lying in the right thalamus. Coordinates are provided in MNI space. Threshold was set at p < 0.001 for the voxel level and p < 0.05 for cluster-level family-wise error correction.

Abbreviations: ALE: activation likelihood estimation

© 2025 Reimann GM et al. *JAMA Psychiatry*.

eFigure 16. Convergent Regional Alterations Based on Adults-Only

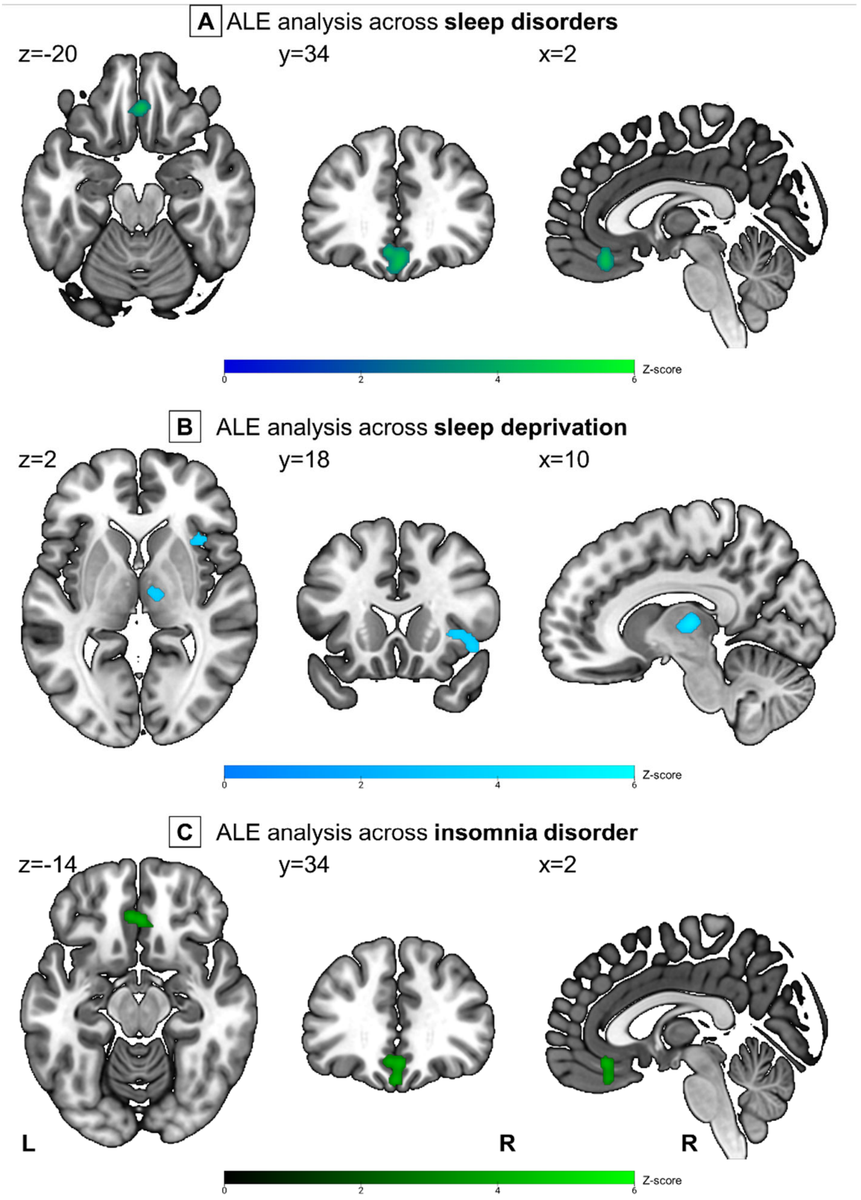

Activation likelihood estimation (ALE) results for adults-only experiments; **A.** Across all sleep disorders identified one clusters, lying in the bilateral subgenual anterior cingulate cortex; **B.** Across sleep deprivation identified one cluster, lying in the right thalamus; **C.** Across insomnia disorder identified one cluster, lying in the bilateral subgenual anterior cingulate cortex. Coordinates are provided in MNI space. Threshold was set at $p < 0.001$ for the voxel level and $p < 0.05$ for cluster-level family-wise error correction.

Abbreviations: ALE: activation likelihood estimation

© 2025 Reimann GM et al. *JAMA Psychiatry*.

eFigure 17. Convergent Regional Alterations Based on Higher Power Experiments

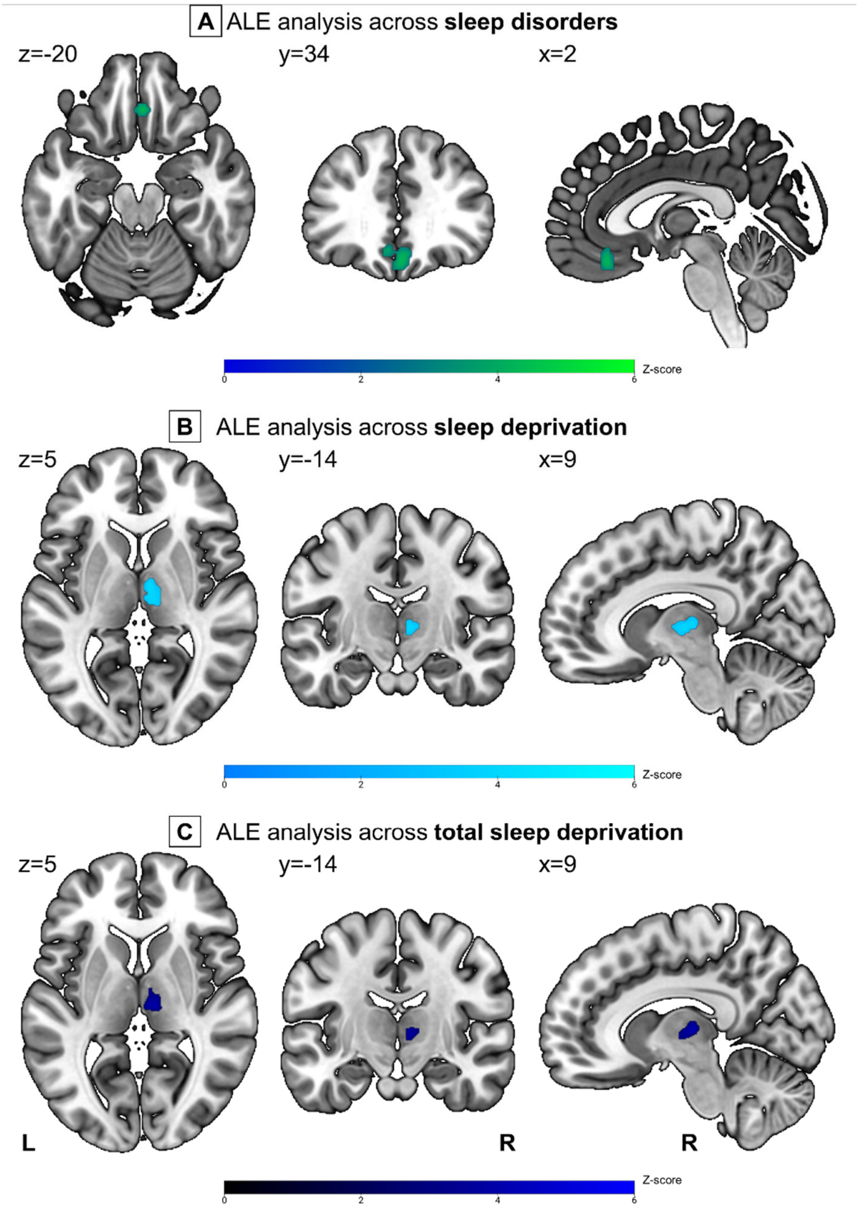

Activation likelihood estimation (ALE) results for experiments of higher power, i.e., sample size ≥ 21; **A.** Across all sleep disorders identified one clusters, lying in the subgenual anterior cingulate cortex; **B.** Across experiments of sleep deprivation identified one cluster, lying in the right thalamus; **C.** Across total sleep deprivation identified one cluster, lying in the right thalamus. The coordinates are in MNI space. Threshold set to cluster-level family-wise error-corrected p-value < 0.05, and of p < 0.001 at the voxel level.

Abbreviations: ALE: activation likelihood estimation

© 2025 Reimann GM et al. *JAMA Psychiatry*.

eFigure 18. Convergent Regional Alterations Based on Corrected Coordinates

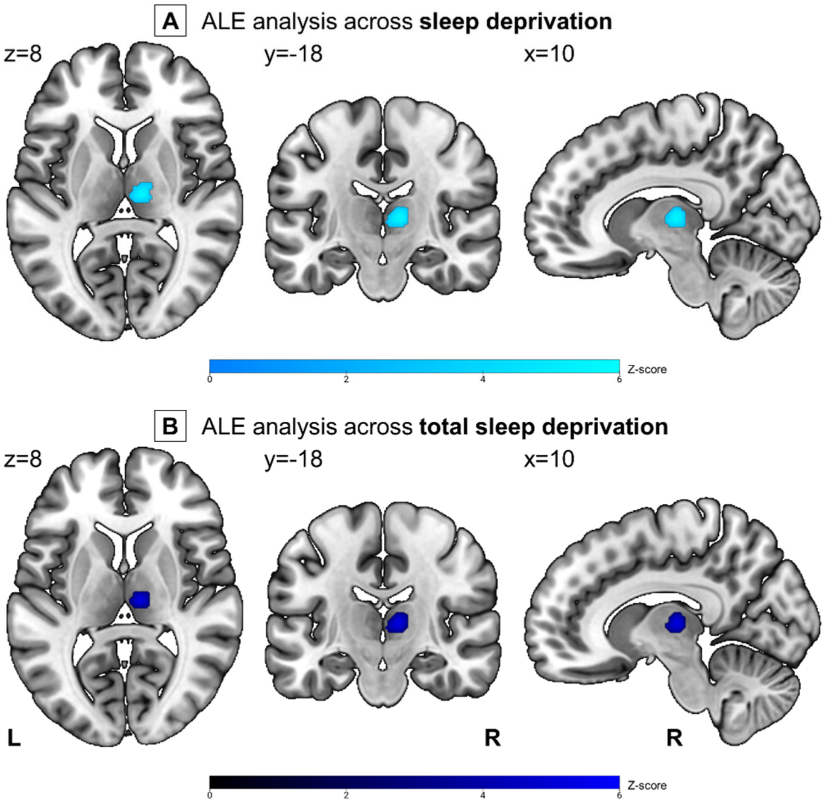

Activation likelihood estimation (ALE) results of only corrected coordinates; **A.** Across sleep deprivation identified one clusters, lying in the right thalamus; **B.** Across total sleep deprivation identified one cluster, lying in the right thalamus. The coordinates are in MNI space. Threshold set to cluster-level family-wise error-corrected p-value < 0.05, and of p < 0.001 at the voxel level.

Abbreviations: ALE: activation likelihood estimation

© 2025 Reimann GM et al. *JAMA Psychiatry*.

eReferences

1. Reimann GM, Küppers V, Camilleri JA, et al. Convergent abnormality in the subgenual anterior cingulate cortex in insomnia disorder: A revisited neuroimaging meta-analysis of 39 studies. *Sleep Medicine Reviews*. 2023/07/15/ 2023:101821. doi:https://doi.org/10.1016/j.smrv.2023.101821
2. Tahmasian M, Rosenzweig I, Eickhoff SB, et al. Structural and functional neural adaptations in obstructive sleep apnea: an activation likelihood estimation meta-analysis. *Neurosci Biobehav Rev*. Mar 30 2016;doi:10.1016/j.neubiorev.2016.03.026
3. Javaheripour N, Shahdipour N, Noori K, et al. Functional brain alterations in acute sleep deprivation: An activation likelihood estimation meta-analysis. *Sleep Med Rev*. Aug 2019;46:64-73. doi:10.1016/j.smrv.2019.03.008
4. Rahimi-Jafari S, Sarebannejad S, Saberi A, et al. Is there any consistent structural and functional brain abnormality in narcolepsy? A meta-analytic perspective. *Neurosci Biobehav Rev*. Jan 2022;132:1181-1182. doi:10.1016/j.neubiorev.2021.10.034
5. Eickhoff SB, Bzdok D, Laird AR, Kurth F, Fox PT. Activation likelihood estimation meta-analysis revisited. *Neuroimage*. Feb 1 2012;59(3):2349-61. doi:10.1016/j.neuroimage.2011.09.017
6. Muller VI, Cieslik EC, Laird AR, et al. Ten simple rules for neuroimaging meta-analysis. *Neurosci Biobehav Rev*. Jan 2018;84:151-161. doi:10.1016/j.neubiorev.2017.11.012
7. Tahmasian M, Sepehry AA, Samea F, et al. Practical recommendations to conduct a neuroimaging meta-analysis for neuropsychiatric disorders. *Hum Brain Mapp*. Dec 1 2019;40(17):5142-5154. doi:10.1002/hbm.24746
8. Eickhoff SB, Nichols TE, Laird AR, et al. Behavior, sensitivity, and power of activation likelihood estimation characterized by massive empirical simulation. *NeuroImage*. Aug 15 2016;137:70-85. doi:10.1016/j.neuroimage.2016.04.072
9. Gray JP, Muller VI, Eickhoff SB, Fox PT. Multimodal Abnormalities of Brain Structure and Function in Major Depressive Disorder: A Meta-Analysis of Neuroimaging Studies. *Am J Psychiatry*. May 1 2020;177(5):422-434. doi:10.1176/appi.ajp.2019.19050560
10. Acar F, Seurinck R, Eickhoff SB, Moerkerke B. Assessing robustness against potential publication bias in Activation Likelihood Estimation (ALE) meta-analyses for fMRI. *PLOS ONE*. 2018;13(11):e0208177. doi:10.1371/journal.pone.0208177
11. Laird AR, Lancaster JL, Fox PT. BrainMap: the social evolution of a human brain mapping database. *Neuroinformatics*. 2005;3(1):65-78. doi:10.1385/ni:3:1:065
12. Laird AR, Eickhoff SB, Li K, Robin DA, Glahn DC, Fox PT. Investigating the functional heterogeneity of the default mode network using coordinate-based meta-analytic modeling. *J Neurosci*. Nov 18 2009;29(46):14496-505. doi:10.1523/JNEUROSCI.4004-09.2009
13. Langner R, Camilleri JA. Meta-Analytic Connectivity Modelling (MACM): A Tool for Assessing Region-Specific Functional Connectivity Patterns in Task-Constrained States. *Brain Network Dysfunction in Neuropsychiatric Illness*. 2021:93-104:chap Chapter 5.
14. Nooner KB, Colcombe SJ, Tobe RH, et al. The NKI-Rockland Sample: A Model for Accelerating the Pace of Discovery Science in Psychiatry. *Front Neurosci*. 2012;6:152. doi:10.3389/fnins.2012.00152
15. Reimann GM, Kuppers V, Camilleri JA, et al. Convergent abnormality in the subgenual anterior cingulate cortex in insomnia disorder: A revisited neuroimaging meta-analysis of 39 studies. *Sleep Med Rev*. Oct 2023;71:101821. doi:10.1016/j.smrv.2023.101821
16. Tahmasian M, Rosenzweig I, Eickhoff SB, et al. Structural and functional neural adaptations in obstructive sleep apnea: An activation likelihood estimation meta-analysis. *Neurosci Biobehav Rev*. Jun 2016;65:142-56. doi:10.1016/j.neubiorev.2016.03.026
17. Nofzinger EA, Buysse DJ, Germain A, Price JC, Miewald JM, Kupfer DJ. Functional neuroimaging evidence for hyperarousal in insomnia. *Am J Psychiatry*. Nov 2004;161(11):2126-8. doi:10.1176/appi.ajp.161.11.2126
18. Ponz A, Khatami R, Poryazova R, et al. Abnormal activity in reward brain circuits in human narcolepsy with cataplexy. *Ann Neurol*. Feb 2010;67(2):190-200. doi:10.1002/ana.21825
19. Draganski B, Geisler P, Hajak G, et al. Hypothalamic gray matter changes in narcoleptic patients. *Nat Med*. Nov 2002;8(11):1186-8. doi:10.1038/nm1102-1186
20. Joo EY, Hong SB, Tae WS, et al. Cerebral perfusion abnormality in narcolepsy with cataplexy. *NeuroImage*. Nov 1 2005;28(2):410-6. doi:10.1016/j.neuroimage.2005.06.019
21. Schaer M, Poryazova R, Schwartz S, Bassetti CL, Baumann CR. Cortical morphometry in narcolepsy with cataplexy. *J Sleep Res*. Oct 2012;21(5):487-94. doi:10.1111/j.1365-2869.2012.01000.x
22. Joo EY, Jeon S, Lee M, et al. Analysis of cortical thickness in narcolepsy patients with cataplexy. *Sleep*. Oct 1 2011;34(10):1357-64. doi:10.5665/SLEEP.1278
23. Kim YK, Yoon IY, Shin YK, Cho SS, Kim SE. Modafinil-induced hippocampal activation in narcolepsy. *Neurosci Lett*. Jul 11 2007;422(2):91-6. doi:10.1016/j.neulet.2007.04.085
24. Thomas M. Neural basis of alertness and cognitive performance impairments during sleepiness II. Effects of 48 and 72 h of sleep deprivation on waking human regional brain activity. *Thalamus & Related Systems*. 2003;2(3):199-229. doi:10.1016/s1472-9288(03)00020-7
25. Rauchs G, Orban P, Schmidt C, et al. Sleep modulates the neural substrates of both spatial and contextual memory consolidation. *PLOS ONE*. Aug 13 2008;3(8):e2949. doi:10.1371/journal.pone.0002949
26. Muto V, Shaffii-le Bourdiec A, Matarazzo L, et al. Influence of acute sleep loss on the neural correlates of alerting, orientating and executive attention components. *J Sleep Res*. Dec 2012;21(6):648-58. doi:10.1111/j.1365-2869.2012.01020.x
27. Benedict C, Brooks SJ, O'Daly OG, et al. Acute sleep deprivation enhances the brain's response to hedonic food stimuli: an fMRI study. *J Clin Endocrinol Metab*. Mar 2012;97(3):E443-7. doi:10.1210/jc.2011-2759
28. Chee MW, Tan JC, Zheng H, et al. Lapsing during sleep deprivation is associated with distributed changes in brain activation. *J Neurosci*. May 21 2008;28(21):5519-28. doi:10.1523/JNEUROSCI.0733-08.2008

29. Chee MW, Tan JC. Lapsing when sleep deprived: neural activation characteristics of resistant and vulnerable individuals. *NeuroImage*. Jun 2010;51(2):835-43. doi:10.1016/j.neuroimage.2010.02.031
30. Albouy G, Vandewalle G, Sterpenich V, et al. Sleep stabilizes visuomotor adaptation memory: a functional magnetic resonance imaging study. *J Sleep Res*. Apr 2013;22(2):144-54. doi:10.1111/j.1365-2869.2012.01059.x
31. Vandewalle G, Archer SN, Wuillaume C, et al. Functional magnetic resonance imaging-assessed brain responses during an executive task depend on interaction of sleep homeostasis, circadian phase, and PER3 genotype. *J Neurosci*. Jun 24 2009;29(25):7948-56. doi:10.1523/JNEUROSCI.0229-09.2009
32. Reichert CF, Maire M, Gabel V, et al. Cognitive brain responses during circadian wake-promotion: evidence for sleep-pressure-dependent hypothalamic activations. *Sci Rep*. Jul 17 2017;7(1):5620. doi:10.1038/s41598-017-05695-1
33. Macey KE, Macey PM, Woo MA, et al. fMRI signal changes in response to forced expiratory loading in congenital central hypoventilation syndrome. *J Appl Physiol (1985)*. Nov 2004;97(5):1897-907. doi:10.1152/japplphysiol.00359.2004
34. Macey PM, Macey KE, Woo MA, Keens TG, Harper RM. Aberrant neural responses to cold pressor challenges in congenital central hypoventilation syndrome. *Pediatr Res*. Apr 2005;57(4):500-9. doi:10.1203/01.PDR.0000155757.98389.53
35. Macey PM, Woo MA, Macey KE, et al. Hypoxia reveals posterior thalamic, cerebellar, midbrain, and limbic deficits in congenital central hypoventilation syndrome. *J Appl Physiol (1985)*. Mar 2005;98(3):958-69. doi:10.1152/japplphysiol.00969.2004
36. Woo MA, Macey PM, Macey KE, et al. FMRI responses to hyperoxia in congenital central hypoventilation syndrome. *Pediatr Res*. Apr 2005;57(4):510-8. doi:10.1203/01.PDR.0000155763.93819.46
37. Dauvilliers Y, Evangelista E, de Verbizier D, Barateau L, Peigneux P. [18F]Fludeoxyglucose-Positron Emission Tomography Evidence for Cerebral Hypermetabolism in the Awake State in Narcolepsy and Idiopathic Hypersomnia. *Front Neurol*. 2017/07/20/ 2017;8:350. doi:10.3389/fneur.2017.00350
38. Trotti LM, Saini P, Crosson B, Meltzer CC, Rye DB, Nye JA. Regional brain metabolism differs between narcolepsy type 1 and idiopathic hypersomnia. *Sleep*. Aug 13 2021;44(8):zsab050. doi:10.1093/sleep/zsab050
39. Altena E, Van Der Werf YD, Sanz-Arigita EJ, et al. Prefrontal hypoactivation and recovery in insomnia. *Sleep*. Sep 2008;31(9):1271-6.
40. Altena E, Vrenken H, Van Der Werf YD, van den Heuvel OA, Van Someren EJ. Reduced orbitofrontal and parietal gray matter in chronic insomnia: a voxel-based morphometric study. *Biol Psychiatry*. Jan 15 2010;67(2):182-5. doi:10.1016/j.biopsych.2009.08.003
41. Stoffers D, Altena E, van der Werf YD, et al. The caudate: a key node in the neuronal network imbalance of insomnia? *Brain*. Feb 2014;137(Pt 2):610-20. doi:10.1093/brain/awt329
42. Baglioni C, Spiegelhalder K, Regen W, et al. Insomnia disorder is associated with increased amygdala reactivity to insomnia-related stimuli. *Sleep*. Dec 1 2014;37(12):1907-17. doi:10.5665/sleep.4240
43. Chen MC, Chang C, Glover GH, Gotlib IH. Increased insula coactivation with salience networks in insomnia. *Biol Psychol*. Mar 2014;97:1-8. doi:10.1016/j.biopsycho.2013.12.016
44. Chen Z, Feng Y, Li S, et al. Altered functional connectivity strength in chronic insomnia associated with gut microbiota composition and sleep efficiency. *Front Psychiatry*. 2022/11/22/ 2022;13:1050403. doi:10.3389/fpsyt.2022.1050403
45. Feng Y, Fu S, Li C, et al. Interaction of Gut Microbiota and Brain Function in Patients With Chronic Insomnia: A Regional Homogeneity Study. *Front Neurosci*. 2022/01/05/ 2021;15:804843. doi:10.3389/fnins.2021.804843
46. Li S, Wang BA, Li C, et al. Progressive gray matter hypertrophy with severity stages of insomnia disorder and its relevance for mood symptoms. *Eur Radiol*. Aug 2021;31(8):6312-6322. doi:10.1007/s00330-021-07701-7
47. Li J, Wang X, Liu M, et al. Sex-specific grey matter abnormalities in individuals with chronic insomnia. *Neurol Sci*. May 2024;45(5):2301-2310. doi:10.1007/s10072-023-07224-7
48. Chen W, Wang H, Sun T, et al. Dynamic changes in fractional amplitude of low-frequency fluctuations in patients with chronic insomnia. *Front Neurosci*. 2022/11/29/ 2022;16:1050240. doi:10.3389/fnins.2022.1050240
49. Chou KH, Lee PL, Liang CS, et al. Identifying neuroanatomical signatures in insomnia and migraine comorbidity. *Sleep*. Mar 12 2021;44(3):zsaa202. doi:10.1093/sleep/zsaa202
50. Dai XJ, Peng DC, Gong HH, et al. Altered intrinsic regional brain spontaneous activity and subjective sleep quality in patients with chronic primary insomnia: a resting-state fMRI study. *Neuropsychiatr Dis Treat*. 2014/11// 2014;10:2163-75. doi:10.2147/NDT.S69681
51. Dai XJ, Nie X, Liu X, et al. Gender Differences in Regional Brain Activity in Patients with Chronic Primary Insomnia: Evidence from a Resting-State fMRI Study. *J Clin Sleep Med*. Mar 2016;12(3):363-74. doi:10.5664/jcsm.5586
52. Dai XJ, Jiang J, Zhang Z, et al. Plasticity and Susceptibility of Brain Morphometry Alterations to Insufficient Sleep. *Front Psychiatry*. 2018/06/27/ 2018;9:266. doi:10.3389/fpsyt.2018.00266
53. Dai XJ, Liu BX, Ai S, et al. Altered inter-hemispheric communication of default-mode and visual networks underlie etiology of primary insomnia : Altered inter-hemispheric communication underlie etiology of insomnia. *Brain Imaging Behav*. Oct 2020;14(5):1430-1444. doi:10.1007/s11682-019-00064-0
54. Liu X, Zheng J, Liu BX, Dai XJ. Altered connection properties of important network hubs may be neural risk factors for individuals with primary insomnia. *Sci Rep*. Apr 12 2018;8(1):5891. doi:10.1038/s41598-018-23699-3
55. Grau-Rivera O, Operto G, Falcon C, et al. Association between insomnia and cognitive performance, gray matter volume, and white matter microstructure in cognitively unimpaired adults. *Alzheimers Res Ther*. Jan 7 2020;12(1):4. doi:10.1186/s13195-019-0547-3
56. Huang S, Zhou F, Jiang J, et al. Regional impairment of intrinsic functional connectivity strength in patients with chronic primary insomnia. *Neuropsychiatr Dis Treat*. 2017/06// 2017;13:1449-1462. doi:10.2147/NDT.S137292

© 2025 Reimann GM et al. *JAMA Psychiatry*.

57. Zhou F, Huang S, Gao L, Zhuang Y, Ding S, Gong H. Temporal regularity of intrinsic cerebral activity in patients with chronic primary insomnia: a brain entropy study using resting-state fMRI. *Brain Behav*. Oct 2016;6(10):e00529. doi:10.1002/brb3.529
58. Zhou F, Huang S, Zhuang Y, Gao L, Gong H. Frequency-dependent changes in local intrinsic oscillations in chronic primary insomnia: A study of the amplitude of low-frequency fluctuations in the resting state. *Neuroimage Clin*. 2017 2017;15:458-465. doi:10.1016/j.nicl.2016.05.011
59. Zhou F, Zhao Y, Huang M, Zeng X, Wang B, Gong H. Disrupted interhemispheric functional connectivity in chronic insomnia disorder: a resting-state fMRI study. *Neuropsychiatr Dis Treat*. 2018/05// 2018;14:1229-1240. doi:10.2147/NDT.S162325
60. Zhou F, Zhu Y, Zhu Y, et al. Altered long- and short-range functional connectivity density associated with poor sleep quality in patients with chronic insomnia disorder: A resting-state fMRI study. *Brain Behav*. Nov 2020;10(11):e01844. doi:10.1002/brb3.1844
61. Ji B, Dai M, Guo Z, et al. Functional Connectivity Density in the Sensorimotor Area is Associated with Sleep Latency in Patients with Primary Insomnia. *Neuropsychiatr Dis Treat*. 2022/01// 2022;18:1-10. doi:10.2147/NDT.S338489
62. Zhang Y, Zhang Z, Wang Y, et al. Dysfunctional beliefs and attitudes about sleep are associated with regional homogeneity of left inferior occidental gyrus in primary insomnia patients: a preliminary resting state functional magnetic resonance imaging study. *Sleep Med*. May 2021;81:188-193. doi:10.1016/j.sleep.2021.02.039
63. Jiang T, Yin X, Zhu L, et al. Abnormal alterations of regional spontaneous neuronal activity and functional connectivity in insomnia patients with difficulty falling asleep: a resting-state fMRI study. *BMC Neurol*. Dec 4 2023;23(1):430. doi:10.1186/s12883-023-03481-3
64. Joo EY, Noh HJ, Kim JS, et al. Brain Gray Matter Deficits in Patients with Chronic Primary Insomnia. *Sleep*. Jul 1 2013;36(7):999-1007. doi:10.5665/sleep.2796
65. Kay DB, Karim HT, Soehner AM, et al. Sleep-Wake Differences in Relative Regional Cerebral Metabolic Rate for Glucose among Patients with Insomnia Compared with Good Sleepers. *Sleep*. Oct 1 2016;39(10):1779-1794. doi:10.5665/sleep.6154
66. Kay DB, Karim HT, Soehner AM, et al. Subjective-Objective Sleep Discrepancy Is Associated With Alterations in Regional Glucose Metabolism in Patients With Insomnia and Good Sleeper Controls. *Sleep*. Nov 1 2017;40(11)doi:10.1093/sleep/zsx155
67. Kim SJ, Lee YJ, Kim N, et al. Exploration of changes in the brain response to sleep-related pictures after cognitive-behavioral therapy for psychophysiological insomnia. *Sci Rep*. Oct 2 2017;7(1):12528. doi:10.1038/s41598-017-13065-0
68. Lee MH, Lee KH, Oh SM, et al. The moderating effect of prefrontal response to sleep-related stimuli on the association between depression and sleep disturbance in insomnia disorder. *Sci Rep*. Oct 22 2022;12(1):17739. doi:10.1038/s41598-022-22652-9
69. Kim YB, Kim N, Lee JJ, Cho SE, Na KS, Kang SG. Brain reactivity using fMRI to insomnia stimuli in insomnia patients with discrepancy between subjective and objective sleep. *Sci Rep*. Jan 15 2021;11(1):1592. doi:10.1038/s41598-021-81219-2
70. Li Y, Liu L, Wang E, et al. Abnormal Neural Network of Primary Insomnia: Evidence from Spatial Working Memory Task fMRI. *Eur Neurol*. 2016 2016;75(1-2):48-57. doi:10.1159/000443372
71. Li G, Zhang X, Zhang J, Wang E, Zhang H, Li Y. Magnetic resonance study on the brain structure and resting-state brain functional connectivity in primary insomnia patients. *Medicine (Baltimore)*. Aug 2018;97(34):e11944. doi:10.1097/MD.0000000000011944
72. Li C, Ma X, Dong M, et al. Abnormal spontaneous regional brain activity in primary insomnia: a resting-state functional magnetic resonance imaging study. *Neuropsychiatr Dis Treat*. 2016/06// 2016;12:1371-8. doi:10.2147/NDT.S109633
73. Li M, Yan J, Li S, et al. Altered gray matter volume in primary insomnia patients: a DARTEL-VBM study. *Brain Imaging Behav*. Dec 2018;12(6):1759-1767. doi:10.1007/s11682-018-9844-x
74. Li S, Tian J, Li M, et al. Altered resting state connectivity in right side frontoparietal network in primary insomnia patients. *Eur Radiol*. Feb 2018;28(2):664-672. doi:10.1007/s00330-017-5012-8
75. Li C, Mai Y, Dong M, et al. Multivariate Pattern Classification of Primary Insomnia Using Three Types of Functional Connectivity Features. *Front Neurol*. 2019/10/02/ 2019;10:1037. doi:10.3389/fneur.2019.01037
76. Meng X, Zheng J, Liu Y, et al. Increased Dynamic Amplitude of Low Frequency Fluctuation in Primary Insomnia. *Front Neurol*. 2020/06/30/ 2020;11:609. doi:10.3389/fneur.2020.00609
77. Wang T, Li S, Jiang G, et al. Regional homogeneity changes in patients with primary insomnia. *Eur Radiol*. May 2016;26(5):1292-300. doi:10.1007/s00330-015-3960-4
78. Qiu G, Wang L, Liao W, et al. Electroacupuncture Activated Impaired Brain Areas and Improved Mental Status and Sleep Quality in Primary Insomnia Patients. *Altern Ther Health Med*. Dec 8 2023:AT9712.
79. Ran Q, Chen J, Li C, et al. Abnormal amplitude of low-frequency fluctuations associated with rapid-eye movement in chronic primary insomnia patients. *Oncotarget*. Oct 17 2017;8(49):84877-84888. doi:10.18632/oncotarget.17921
80. Santarnecchi E, Del Bianco C, Sicilia I, et al. Age of Insomnia Onset Correlates with a Reversal of Default Mode Network and Supplementary Motor Cortex Connectivity. *Neural Plast*. 2018 2018;2018:3678534. doi:10.1155/2018/3678534
81. Seo J, Moore KN, Gazecki S, et al. Delayed fear extinction in individuals with insomnia disorder. *Sleep*. Aug 1 2018;41(8)doi:10.1093/sleep/zsy095
82. Shao Y, Zou G, Tabarak S, et al. Spindle-related brain activation in patients with insomnia disorder: An EEG-fMRI study. *Brain Imaging Behav*. Apr 2022;16(2):659-670. doi:10.1007/s11682-021-00544-2
83. Son YD, Kang JM, Cho SJ, Lee JS, Hwang HY, Kang SG. fMRI brain activation in patients with insomnia disorder during a working memory task. *Sleep Breath*. May 2018;22(2):487-493. doi:10.1007/s11325-017-1575-5

84. Wassing R, Schalkwijk F, Lakbila-Kamal O, et al. Haunted by the past: old emotions remain salient in insomnia disorder. *Brain*. Jun 1 2019;142(6):1783-1796. doi:10.1093/brain/awz089
85. Yan CQ, Liu CZ, Wang X, et al. Abnormal Functional Connectivity of Anterior Cingulate Cortex in Patients With Primary Insomnia: A Resting-State Functional Magnetic Resonance Imaging Study. *Front Aging Neurosci*. 2018/06/05/ 2018;10:167. doi:10.3389/fnagi.2018.00167
86. Yan CQ, Wang X, Huo JW, et al. Abnormal Global Brain Functional Connectivity in Primary Insomnia Patients: A Resting-State Functional MRI Study. *Front Neurol*. 2018/11/02/ 2018;9:856. doi:10.3389/fneur.2018.00856
87. Yu S, Guo B, Shen Z, et al. The imbalanced anterior and posterior default mode network in the primary insomnia. *J Psychiatr Res*. Aug 2018;103:97-103. doi:10.1016/j.jpsychires.2018.05.013
88. Zhao B, Bi Y, Li L, et al. The Instant Spontaneous Neuronal Activity Modulation of Transcutaneous Auricular Vagus Nerve Stimulation on Patients With Primary Insomnia. *Front Neurosci*. 2020/03/13/ 2020;14:205. doi:10.3389/fnins.2020.00205
89. Zheng H, Zhou Q, Yang J, et al. Altered functional connectivity of the default mode and frontal control networks in patients with insomnia. *CNS Neurosci Ther*. Aug 2023;29(8):2318-2326. doi:10.1111/cns.14183
90. Engstrom M, Vigren P, Karlsson T, Landtblom AM. Working memory in 8 Kleine-Levin syndrome patients: an fMRI study. *Sleep*. May 2009;32(5):681-8. doi:10.1093/sleep/32.5.681
91. Brenneis C, Brandauer E, Frauscher B, et al. Voxel-based morphometry in narcolepsy. *Sleep Med*. Nov 2005;6(6):531-6. doi:10.1016/j.sleep.2005.03.015
92. Dauvilliers Y, Comte F, Bayard S, Carlander B, Zanca M, Touchon J. A brain PET study in patients with narcolepsy-cataplexy. *J Neurol Neurosurg Psychiatry*. Mar 2010;81(3):344-8. doi:10.1136/jnnp.2009.175786
93. Fulong X, Chao L, Dianjiang Z, et al. Recursive Partitioning Analysis of Fractional Low-Frequency Fluctuations in Narcolepsy With Cataplexy. *Front Neurol*. 2018/11/02/ 2018;9:936. doi:10.3389/fneur.2018.00936
94. Xiao F, Lu C, Zhao D, et al. Independent Component Analysis and Graph Theoretical Analysis in Patients with Narcolepsy. *Neurosci Bull*. Aug 2019;35(4):743-755. doi:10.1007/s12264-018-0307-6
95. Fulong X, Spruyt K, Chao L, Dianjiang Z, Jun Z, Fang H. Resting-state brain network topological properties and the correlation with neuropsychological assessment in adolescent narcolepsy. *Sleep*. Aug 12 2020;43(8):zsaa018. doi:10.1093/sleep/zsaa018
96. Gool JK, van der Werf YD, Lammers GJ, Fronczek R. The Sustained Attention to Response Task Shows Lower Cingulo-Opercular and Frontoparietal Activity in People with Narcolepsy Type 1: An fMRI Study on the Neural Regulation of Attention. *Brain Sci*. Jul 1 2020;10(7):419. doi:10.3390/brainsci10070419
97. Gool JK, Fronczek R, Bosma P, van der Meer JN, van der Werf YD, Lammers GJ. Enhanced Visual Cortex Activation in People With Narcolepsy Type 1 During Active Sleep Resistance: An fMRI-EEG Study. *Front Neurosci*. 2022/06/27/ 2022;16:904820. doi:10.3389/fnins.2022.904820
98. van Holst RJ, Janssen LK, van Mierlo P, et al. Enhanced food-related responses in the ventral medial prefrontal cortex in narcolepsy type 1. *Sci Rep*. Nov 6 2018;8(1):16391. doi:10.1038/s41598-018-34647-6
99. Joo EY, Tae WS, Kim JH, Kim BT, Hong SB. Glucose hypometabolism of hypothalamus and thalamus in narcolepsy. *Ann Neurol*. Sep 2004;56(3):437-40. doi:10.1002/ana.20212
100. Joo EY, Tae WS, Kim ST, Hong SB. Gray matter concentration abnormality in brains of narcolepsy patients. *Korean J Radiol*. Nov-Dec 2009;10(6):552-8. doi:10.3348/kjr.2009.10.6.552
101. Kaufmann C, Schuld A, Pollmacher T, Auer DP. Reduced cortical gray matter in narcolepsy: preliminary findings with voxel-based morphometry. *Neurology*. Jun 25 2002;58(12):1852-5. doi:10.1212/wnl.58.12.1852
102. Kim SJ, Lyoo IK, Lee YS, et al. Gray matter deficits in young adults with narcolepsy. *Acta Neurol Scand*. Jan 2009;119(1):61-7. doi:10.1111/j.1600-0404.2008.01063.x
103. Reiss AL, Hoeft F, Tenforde AS, Chen W, Mobbs D, Mignot EJ. Anomalous hypothalamic responses to humor in cataplexy. *PLOS ONE*. May 21 2008;3(5):e2225. doi:10.1371/journal.pone.0002225
104. Scherfler C, Frauscher B, Schocke M, et al. White and gray matter abnormalities in narcolepsy with cataplexy. *Sleep*. Mar 1 2012;35(3):345-51. doi:10.5665/sleep.1692
105. Schwartz S, Ponz A, Poryazova R, et al. Abnormal activity in hypothalamus and amygdala during humour processing in human narcolepsy with cataplexy. *Brain*. Feb 2008;131(Pt 2):514-22. doi:10.1093/brain/awm292
106. Tondelli M, Pizza F, Vaudano AE, Plazzi G, Meletti S. Cortical and Subcortical Brain Changes in Children and Adolescents With Narcolepsy Type 1. *Sleep*. Feb 1 2018;41(2)doi:10.1093/sleep/zsx192
107. Wu L, Zhan Q, Liu Q, et al. Abnormal Regional Spontaneous Neural Activity and Functional Connectivity in Unmedicated Patients with Narcolepsy Type 1: A Resting-State fMRI Study. *Int J Environ Res Public Health*. Nov 22 2022;19(23):15482. doi:10.3390/ijerph192315482
108. Xu L, Xue R, Ai Z, et al. Resting-State Functional Magnetic Resonance Imaging as an Indicator of Neuropsychological Changes in Type 1 Narcolepsy. *Acad Radiol*. Jan 2024;31(1):69-81. doi:10.1016/j.acra.2023.08.026
109. Shen C, Wang J, Ma G, et al. Waking-hour cerebral activations in nightmare disorder: A resting-state functional magnetic resonance imaging study. *Psychiatry Clin Neurosci*. Dec 2016;70(12):573-581. doi:10.1111/pcn.12455
110. Andre C, Rehel S, Kuhn E, et al. Association of Sleep-Disordered Breathing With Alzheimer Disease Biomarkers in Community-Dwelling Older Adults: A Secondary Analysis of a Randomized Clinical Trial. *JAMA Neurol*. Jun 1 2020;77(6):716-724. doi:10.1001/jamaneurol.2020.0311
111. Ayalon L, Ancoli-Israel S, Klemfuss Z, Shalauta MD, Drummond SP. Increased brain activation during verbal learning in obstructive sleep apnea. *NeuroImage*. Jul 15 2006;31(4):1817-25. doi:10.1016/j.neuroimage.2006.02.042
112. Ayalon L, Ancoli-Israel S, Drummond SP. Altered brain activation during response inhibition in obstructive sleep apnea. *J Sleep Res*. Jun 2009;18(2):204-8. doi:10.1111/j.1365-2869.2008.00707.x

© 2025 Reimann GM et al. *JAMA Psychiatry*.

113. Ayalon L, Ancoli-Israel S, Aka AA, McKenna BS, Drummond SP. Relationship between obstructive sleep apnea severity and brain activation during a sustained attention task. *Sleep*. Mar 2009;32(3):373-81. doi:10.1093/sleep/32.3.373
114. Bai J, Wen H, Tai J, et al. Altered Spontaneous Brain Activity Related to Neurologic and Sleep Dysfunction in Children With Obstructive Sleep Apnea Syndrome. *Front Neurosci*. 2021/11/15/ 2021;15:595412. doi:10.3389/fnins.2021.595412
115. Ji T, Li X, Chen J, et al. Brain function in children with obstructive sleep apnea: a resting-state fMRI study. *Sleep*. Aug 13 2021;44(8):zsab047. doi:10.1093/sleep/zsab047
116. Baima CB, Fim NC, Alves KF, Resende LAL, Fonseca RG, Betting LE. Analysis of patients with obstructive sleep apnea with and without pharyngeal myopathy using brain neuroimaging. *Sleep*. Feb 13 2020;43(2):zsz216. doi:10.1093/sleep/zsz216
117. Canessa N, Castronovo V, Cappa SF, et al. Obstructive sleep apnea: brain structural changes and neurocognitive function before and after treatment. *Am J Respir Crit Care Med*. May 15 2011;183(10):1419-26. doi:10.1164/rccm.201005-0693OC
118. Castronovo V, Canessa N, Strambi LF, et al. Brain activation changes before and after PAP treatment in obstructive sleep apnea. *Sleep*. Sep 2009;32(9):1161-72. doi:10.1093/sleep/32.9.1161
119. Celle S, Peyron R, Faillenot I, et al. Undiagnosed sleep-related breathing disorders are associated with focal brainstem atrophy in the elderly. *Hum Brain Mapp*. Jul 2009;30(7):2090-7. doi:10.1002/hbm.20650
120. Chen YS, Chen MH, Wang PM, Lu CH, Chen HL, Lin WC. Increased Levels of Plasma Alzheimer's Disease Biomarkers and Their Associations with Brain Structural Changes and Carotid Intima-Media Thickness in Cognitively Normal Obstructive Sleep Apnea Patients. *Diagnostics (Basel)*. Jun 22 2022;12(7):1522. doi:10.3390/diagnostics12071522
121. Lin WC, Huang CC, Chen HL, et al. Longitudinal brain structural alterations and systemic inflammation in obstructive sleep apnea before and after surgical treatment. *J Transl Med*. May 17 2016;14(1):139. doi:10.1186/s12967-016-0887-8
122. Fatouleh RH, Hammam E, Lundblad LC, et al. Functional and structural changes in the brain associated with the increase in muscle sympathetic nerve activity in obstructive sleep apnoea. *Neuroimage Clin*. 2014 2014;6:275-83. doi:10.1016/j.nicl.2014.08.021
123. Fernandes M, Mari L, Chiaravalloti A, et al. (18)F-FDG PET, cognitive functioning, and CSF biomarkers in patients with obstructive sleep apnoea before and after continuous positive airway pressure treatment. *J Neurol*. Oct 2022;269(10):5356-5367. doi:10.1007/s00415-022-11182-z
124. Fernandes M, Chiaravalloti A, Manfredi N, et al. Nocturnal Hypoxia and Sleep Fragmentation May Drive Neurodegenerative Processes: The Compared Effects of Obstructive Sleep Apnea Syndrome and Periodic Limb Movement Disorder on Alzheimer's Disease Biomarkers. *J Alzheimers Dis*. 2022/06/28/ 2022;88(1):127-139. doi:10.3233/JAD-215734
125. Gao J, Cao J, Chen J, et al. Brain morphology and functional connectivity alterations in patients with severe obstructive sleep apnea. *Sleep Med*. Nov 2023;111:62-69. doi:10.1016/j.sleep.2023.08.032
126. Harper RM, Macey PM, Henderson LA, et al. fMRI responses to cold pressor challenges in control and obstructive sleep apnea subjects. *J Appl Physiol (1985)*. Apr 2003;94(4):1583-95. doi:10.1152/japplphysiol.00881.2002
127. Henderson LA, Woo MA, Macey PM, et al. Neural responses during Valsalva maneuvers in obstructive sleep apnea syndrome. *J Appl Physiol (1985)*. Mar 2003;94(3):1063-74. doi:10.1152/japplphysiol.00702.2002
128. Macey PM, Macey KE, Henderson LA, et al. Functional magnetic resonance imaging responses to expiratory loading in obstructive sleep apnea. *Respir Physiol Neurobiol*. Nov 14 2003;138(2-3):275-90. doi:10.1016/j.resp.2003.09.002
129. Macey KE, Macey PM, Woo MA, et al. Inspiratory loading elicits aberrant fMRI signal changes in obstructive sleep apnea. *Respir Physiol Neurobiol*. Mar 28 2006;151(1):44-60. doi:10.1016/j.resp.2005.05.024
130. Huynh NT, Prilipko O, Kushida CA, Guilleminault C. Volumetric Brain Morphometry Changes in Patients with Obstructive Sleep Apnea Syndrome: Effects of CPAP Treatment and Literature Review. *Front Neurol*. 2014/04/29/ 2014;5:58. doi:10.3389/fneur.2014.00058
131. Prilipko O, Huynh N, Schwartz S, et al. Task positive and default mode networks during a parametric working memory task in obstructive sleep apnea patients and healthy controls. *Sleep*. Mar 1 2011;34(3):293-301A. doi:10.1093/sleep/34.3.293
132. Joo EY, Tae WS, Lee MJ, et al. Reduced brain gray matter concentration in patients with obstructive sleep apnea syndrome. *Sleep*. Feb 2010;33(2):235-41. doi:10.1093/sleep/33.2.235
133. Kim H, Joo E, Suh S, Kim JH, Kim ST, Hong SB. Effects of long-term treatment on brain volume in patients with obstructive sleep apnea syndrome. *Hum Brain Mapp*. Jan 2016;37(1):395-409. doi:10.1002/hbm.23038
134. Kang D, Qin Z, Wang W, et al. Brain functional changes in tibetan with obstructive sleep apnea hypopnea syndrome: A resting state fMRI study. *Medicine (Baltimore)*. Feb 2020;99(7):e18957. doi:10.1097/MD.0000000000018957
135. Li HJ, Dai XJ, Gong HH, Nie X, Zhang W, Peng DC. Aberrant spontaneous low-frequency brain activity in male patients with severe obstructive sleep apnea revealed by resting-state functional MRI. *Neuropsychiatr Dis Treat*. 2015/01// 2015;11:207-14. doi:10.2147/NDT.S73730
136. Li H, Li L, Shao Y, et al. Abnormal Intrinsic Functional Hubs in Severe Male Obstructive Sleep Apnea: Evidence from a Voxel-Wise Degree Centrality Analysis. *PLOS ONE*. 2016/10/10/ 2016;11(10):e0164031. doi:10.1371/journal.pone.0164031
137. Li H, Li L, Kong L, et al. Frequency-Specific Regional Homogeneity Alterations and Cognitive Function in Obstructive Sleep Apnea Before and After Short-Term Continuous Positive Airway Pressure Treatment. *Nat Sci Sleep*. 2021/12// 2021;13:2221-2238. doi:10.2147/NSS.S344842
138. Li P, Shu Y, Liu X, et al. The Effects of CPAP Treatment on Resting-State Network Centrality in Obstructive Sleep Apnea Patients. *Front Neurol*. 2022/03/28/ 2022;13:801121. doi:10.3389/fneur.2022.801121
139. Li K, Shu Y, Liu X, et al. Dynamic regional homogeneity alterations and cognitive impairment in patients with moderate and severe obstructive sleep apnea. *Front Neurosci*. 2022/08/26/ 2022;16:940721. doi:10.3389/fnins.2022.940721
140. Peng DC, Dai XJ, Gong HH, Li HJ, Nie X, Zhang W. Altered intrinsic regional brain activity in male patients with severe obstructive sleep apnea: a resting-state functional magnetic resonance imaging study. *Neuropsychiatr Dis Treat*. 2014/09// 2014;10:1819-26. doi:10.2147/NDT.S67805

© 2025 Reimann GM et al. *JAMA Psychiatry*.

141. Shu Y, Chen L, Li K, et al. Abnormal cerebellar-prefrontal cortical pathways in obstructive sleep apnea with/without mild cognitive impairment. *Front Neurosci*. 2022/10/20/ 2022;16:1002184. doi:10.3389/fnins.2022.1002184
142. Xie W, Shu Y, Liu X, et al. Abnormal Spontaneous Brain Activity and Cognitive Impairment in Obstructive Sleep Apnea. *Nat Sci Sleep*. 2022/09// 2022;14:1575-1587. doi:10.2147/NSS.S376638
143. Zeng Y, Shu Y, Liu X, et al. Frequency-specific alterations in intrinsic low-frequency oscillations in newly diagnosed male patients with obstructive sleep apnea. *Front Neurosci*. 2022/09/30/ 2022;16:987015. doi:10.3389/fnins.2022.987015
144. Liu YT, Zhang HX, Li HJ, et al. Aberrant Interhemispheric Connectivity in Obstructive Sleep Apnea-Hypopnea Syndrome. *Front Neurol*. 2018/05/08/ 2018;9:314. doi:10.3389/fneur.2018.00314
145. Morrell MJ, Jackson ML, Twigg GL, et al. Changes in brain morphology in patients with obstructive sleep apnoea. *Thorax*. Oct 2010;65(10):908-14. doi:10.1136/thx.2009.126730
146. Philby MF, Macey PM, Ma RA, Kumar R, Gozal D, Kheirandish-Gozal L. Reduced Regional Grey Matter Volumes in Pediatric Obstructive Sleep Apnea. *Sci Rep*. Mar 17 2017;7(1):44566. doi:10.1038/srep44566
147. Qin Z, Kang D, Feng X, Kong D, Wang F, Bao H. Resting-state functional magnetic resonance imaging of high altitude patients with obstructive sleep apnoea hypopnoea syndrome. *Sci Rep*. Sep 23 2020;10(1):15546. doi:10.1038/s41598-020-72339-2
148. Ruan Z, Xia Z, Zhang Q, Huang Z, Lv Q. Continuous Positive Airway Pressure Effect on the Changes of Regional Brain Activity in Patients with Severe Obstructive Sleep Apnea-Hypopnea Syndrome. *Journal of Biological Regulators and Homeostatic Agents*. 2023-02-20 2023;37(2):837-844. doi:10.23812/j.biol.regul.homeost.agents.20233702.85
149. Santarnecchi E, Sicilia I, Richiardi J, et al. Altered cortical and subcortical local coherence in obstructive sleep apnea: a functional magnetic resonance imaging study. *J Sleep Res*. Jun 2013;22(3):337-47. doi:10.1111/jsr.12006
150. Santarnecchi E, Sprugnoli G, Sicilia I, et al. Thalamic altered spontaneous activity and connectivity in obstructive sleep apnea syndrome. *J Neuroimaging*. Mar 2022;32(2):314-327. doi:10.1111/jon.12952
151. Sun Y, Yang SX, Xie M, Zou K, Tang X. Aberrant amplitude of low-frequency fluctuations in different frequency bands and changes after one-night positive airway pressure treatment in severe obstructive sleep apnea. *Front Neurol*. 2022/08/22/ 2022;13:985321. doi:10.3389/fneur.2022.985321
152. Sun Y, Lei F, Luo L, Zou K, Tang X. Effects of a single night of continuous positive airway pressure on spontaneous brain activity in severe obstructive sleep apnea. *Sci Rep*. Jun 2 2023;13(1):8950. doi:10.1038/s41598-023-36206-0
153. Torelli F, Moscufo N, Garreffa G, et al. Cognitive profile and brain morphological changes in obstructive sleep apnea. *NeuroImage*. Jan 15 2011;54(2):787-93. doi:10.1016/j.neuroimage.2010.09.065
154. Xiao P, Hua K, Chen F, et al. Abnormal Cerebral Blood Flow and Volumetric Brain Morphometry in Patients With Obstructive Sleep Apnea. *Front Neurosci*. 2022/07/06/ 2022;16:934166. doi:10.3389/fnins.2022.934166
155. Yaouhi K, Bertran F, Clochon P, et al. A combined neuropsychological and brain imaging study of obstructive sleep apnea. *J Sleep Res*. Mar 2009;18(1):36-48. doi:10.1111/j.1365-2869.2008.00705.x
156. Yu C, Fu Y, Lu Y, et al. Alterations of brain gray matter volume in children with obstructive sleep apnea. *Front Neurol*. 2023/05/16/ 2023;14:1107086. doi:10.3389/fneur.2023.1107086
157. Zhang Q, Wang D, Qin W, et al. Altered resting-state brain activity in obstructive sleep apnea. *Sleep*. May 1 2013;36(5):651-659B. doi:10.5665/sleep.2620
158. Zhou L, Shan X, Peng Y, et al. Reduced regional homogeneity and neurocognitive impairment in patients with moderate-to-severe obstructive sleep apnea. *Sleep Med*. Nov 2020;75:418-427. doi:10.1016/j.sleep.2020.09.009
159. Bourgouin PA, Rahayel S, Gaubert M, et al. Gray matter substrates of depressive and anxiety symptoms in idiopathic REM sleep behavior disorder. *Parkinsonism Relat Disord*. May 2019;62:163-170. doi:10.1016/j.parkreldis.2018.12.020
160. Rahayel S, Montplaisir J, Monchi O, et al. Patterns of cortical thinning in idiopathic rapid eye movement sleep behavior disorder. *Mov Disord*. Apr 15 2015;30(5):680-7. doi:10.1002/mds.25820
161. Rahayel S, Postuma RB, Montplaisir J, et al. Abnormal Gray Matter Shape, Thickness, and Volume in the Motor Cortico-Subcortical Loop in Idiopathic Rapid Eye Movement Sleep Behavior Disorder: Association with Clinical and Motor Features. *Cereb Cortex*. Feb 1 2018;28(2):658-671. doi:10.1093/cercor/bhx137
162. Brcina N, Hohenfeld C, Heidbreder A, et al. Increased neural motor activation and functional reorganization in patients with idiopathic rapid eye movement sleep behavior disorder. *Parkinsonism Relat Disord*. Nov 2021;92:76-82. doi:10.1016/j.parkreldis.2021.10.019
163. Chen M, Li Y, Chen J, et al. Structural and functional brain alterations in patients with idiopathic rapid eye movement sleep behavior disorder. *J Neuroradiol*. Jan 2022;49(1):66-72. doi:10.1016/j.neurad.2020.04.007
164. Ge J, Wu P, Peng S, et al. Assessing cerebral glucose metabolism in patients with idiopathic rapid eye movement sleep behavior disorder. *J Cereb Blood Flow Metab*. Dec 2015;35(12):2062-9. doi:10.1038/jcbfm.2015.173
165. Wu P, Yu H, Peng S, et al. Consistent abnormalities in metabolic network activity in idiopathic rapid eye movement sleep behaviour disorder. *Brain*. Dec 2014;137(Pt 12):3122-8. doi:10.1093/brain/awu290
166. Han XH, Li XM, Tang WJ, et al. Assessing gray matter volume in patients with idiopathic rapid eye movement sleep behavior disorder. *Neural Regen Res*. May 2019;14(5):868-875. doi:10.4103/1673-5374.249235
167. Hanyu H, Inoue Y, Sakurai H, et al. Voxel-based magnetic resonance imaging study of structural brain changes in patients with idiopathic REM sleep behavior disorder. *Parkinsonism Relat Disord*. Feb 2012;18(2):136-9. doi:10.1016/j.parkreldis.2011.08.023
168. Jiang X, Wu Z, Zhong M, et al. Abnormal Gray Matter Volume and Functional Connectivity in Parkinson's Disease with Rapid Eye Movement Sleep Behavior Disorder. *Parkinsons Dis*. 2021/02/22/ 2021;2021:8851027. doi:10.1155/2021/8851027
169. Jiang X, Pan Y, Zhu S, et al. Alterations of Regional Homogeneity in Parkinson's Disease with Rapid Eye Movement Sleep Behavior Disorder. *Neuropsychiatr Dis Treat*. 2022/12// 2022;18:2967-2978. doi:10.2147/NDT.S384752
170. Kim R, Lee JY, Kim YK, et al. Longitudinal Changes in Isolated Rapid Eye Movement Sleep Behavior Disorder-Related Metabolic Pattern Expression. *Mov Disord*. Aug 2021;36(8):1889-1898. doi:10.1002/mds.28592

© 2025 Reimann GM et al. *JAMA Psychiatry*.

171. Lim JS, Shin SA, Lee JY, Nam H, Lee JY, Kim YK. Neural substrates of rapid eye movement sleep behavior disorder in Parkinson's disease. *Parkinsonism Relat Disord*. Feb 2016;23:31-6. doi:10.1016/j.parkreldis.2015.11.027
172. Yoon EJ, Lee JY, Nam H, et al. A New Metabolic Network Correlated with Olfactory and Executive Dysfunctions in Idiopathic Rapid Eye Movement Sleep Behavior Disorder. *J Clin Neurol*. Apr 2019;15(2):175-183. doi:10.3988/jcn.2019.15.2.175
173. Li D, Huang P, Zang Y, et al. Abnormal baseline brain activity in Parkinson's disease with and without REM sleep behavior disorder: A resting-state functional MRI study. *J Magn Reson Imaging*. Sep 2017;46(3):697-703. doi:10.1002/jmri.25571
174. Li G, Chen Z, Zhou L, et al. Abnormal intrinsic brain activity of the putamen is correlated with dopamine deficiency in idiopathic rapid eye movement sleep behavior disorder. *Sleep Med*. Nov 2020;75:73-80. doi:10.1016/j.sleep.2019.09.015
175. Mattioli P, Pardini M, Fama F, et al. Cuneus/precuneus as a central hub for brain functional connectivity of mild cognitive impairment in idiopathic REM sleep behavior patients. *Eur J Nucl Med Mol Imaging*. Aug 2021;48(9):2834-2845. doi:10.1007/s00259-021-05205-6
176. Rahayel S, Gaubert M, Postuma RB, et al. Brain atrophy in Parkinson's disease with polysomnography-confirmed REM sleep behavior disorder. *Sleep*. Jun 11 2019;42(6):zsz062. doi:10.1093/sleep/zsz062
177. Salsone M, Cerasa A, Arabia G, et al. Reduced thalamic volume in Parkinson disease with REM sleep behavior disorder: volumetric study. *Parkinsonism Relat Disord*. Sep 2014;20(9):1004-8. doi:10.1016/j.parkreldis.2014.06.012
178. Scherfler C, Frauscher B, Schocke M, et al. White and gray matter abnormalities in idiopathic rapid eye movement sleep behavior disorder: a diffusion-tensor imaging and voxel-based morphometry study. *Ann Neurol*. Feb 2011;69(2):400-7. doi:10.1002/ana.22245
179. Woo KA, Kim H, Yoon EJ, et al. Brain olfactory-related atrophy in isolated rapid eye movement sleep behavior disorder. *Ann Clin Transl Neurol*. Dec 2023;10(12):2192-2207. doi:10.1002/acn3.51905
180. Zhang HJ, Wang SH, Bai YY, Zhang JW, Chen S. Abnormal Striatal-Cortical Networks Contribute to the Attention/Executive Function Deficits in Idiopathic REM Sleep Behavior Disorder: A Resting State Functional MRI Study. *Front Aging Neurosci*. 2021/07/01/ 2021;13:690854. doi:10.3389/fnagi.2021.690854
181. Etgen T, Draganski B, Ilg C, et al. Bilateral thalamic gray matter changes in patients with restless legs syndrome. *NeuroImage*. Feb 15 2005;24(4):1242-7. doi:10.1016/j.neuroimage.2004.10.021
182. Hornyak M, Ahrendts JC, Spiegelhalder K, et al. Voxel-based morphometry in unmedicated patients with restless legs syndrome. *Sleep Med*. Dec 2007;9(1):22-6. doi:10.1016/j.sleep.2006.09.010
183. Li T, Liu C, Lyu H, et al. Alterations of Sub-cortical Gray Matter Volume and Their Associations With Disease Duration in Patients With Restless Legs Syndrome. *Front Neurol*. 2018/12/17/ 2018;9:1098. doi:10.3389/fneur.2018.01098
184. Liu C, Dai Z, Zhang R, et al. Mapping intrinsic functional brain changes and repetitive transcranial magnetic stimulation neuromodulation in idiopathic restless legs syndrome: a resting-state functional magnetic resonance imaging study. *Sleep Med*. Jun 2015;16(6):785-91. doi:10.1016/j.sleep.2014.12.029
185. Liu C, Wang J, Hou Y, et al. Mapping the changed hubs and corresponding functional connectivity in idiopathic restless legs syndrome. *Sleep Med*. May 2018;45:132-139. doi:10.1016/j.sleep.2017.12.016
186. Zhang J, Zhang J, Sun H, et al. Cerebellum drives functional dysfunctions in restless leg syndrome. *Sleep Med*. Oct 2023;110:172-178. doi:10.1016/j.sleep.2023.08.014
187. Stefani A, Mitterling T, Heidbreder A, et al. Multimodal Magnetic Resonance Imaging reveals alterations of sensorimotor circuits in restless legs syndrome. *Sleep*. Dec 24 2019;42(12):zsz171. doi:10.1093/sleep/zsz171
188. Tuovinen N, Stefani A, Mitterling T, et al. Functional connectivity and topology in patients with restless legs syndrome: a case-control resting-state functional magnetic resonance imaging study. *Eur J Neurol*. Feb 2021;28(2):448-458. doi:10.1111/ene.14577
189. Yang FC, Chou KH, Lee PL, et al. Patterns of gray matter alterations in migraine and restless legs syndrome. *Ann Clin Transl Neurol*. Jan 2019;6(1):57-67. doi:10.1002/acn3.680
190. Zhuo Y, Wu Y, Xu Y, et al. Combined resting state functional magnetic resonance imaging and diffusion tensor imaging study in patients with idiopathic restless legs syndrome. *Sleep Med*. Oct 2017;38:96-103. doi:10.1016/j.sleep.2017.06.033
191. Heidbreder A, Stefani A, Brandauer E, et al. Gray matter abnormalities of the dorsal posterior cingulate in sleep walking. *Sleep Med*. Aug 2017;36:152-155. doi:10.1016/j.sleep.2017.05.007
192. Almklov EL, Drummond SP, Orff H, Alhassoon OM. The effects of sleep deprivation on brain functioning in older adults. *Behav Sleep Med*. 2015/07/04/ 2015;13(4):324-45. doi:10.1080/15402002.2014.905474
193. Bell-McGinty S, Habeck C, Hilton HJ, et al. Identification and differential vulnerability of a neural network in sleep deprivation. *Cereb Cortex*. May 2004;14(5):496-502. doi:10.1093/cercor/bhh011
194. Habeck C, Rakitin BC, Moeller J, et al. An event-related fMRI study of the neurobehavioral impact of sleep deprivation on performance of a delayed-match-to-sample task. *Brain Res Cogn Brain Res*. Feb 2004;18(3):306-21. doi:10.1016/j.cogbrainres.2003.10.019
195. Ben Simon E, Walker MP. Sleep loss causes social withdrawal and loneliness. *Nat Commun*. Aug 14 2018;9(1):3146. doi:10.1038/s41467-018-05377-0
196. Ben Simon E, Rossi A, Harvey AG, Walker MP. Overanxious and underslept. *Nat Hum Behav*. Jan 2020;4(1):100-110. doi:10.1038/s41562-019-0754-8
197. Ben Simon E, Vallat R, Rossi A, Walker MP. Sleep loss leads to the withdrawal of human helping across individuals, groups, and large-scale societies. *PLoS Biol*. Aug 2022;20(8):e3001733. doi:10.1371/journal.pbio.3001733
198. Greer SM, Goldstein AN, Knutson B, Walker MP. A Genetic Polymorphism of the Human Dopamine Transporter Determines the Impact of Sleep Deprivation on Brain Responses to Rewards and Punishments. *J Cogn Neurosci*. Jun 2016;28(6):803-10. doi:10.1162/jocn_a_00939

© 2025 Reimann GM et al. *JAMA Psychiatry*.

199. Cai Y, Mai Z, Li M, Zhou X, Ma N. Altered frontal connectivity after sleep deprivation predicts sustained attentional impairment: A resting-state functional magnetic resonance imaging study. *J Sleep Res*. Oct 2021;30(5):e13329. doi:10.1111/jsr.13329
200. Cao Q, Zeng H, Liu F, et al. Changes in brain function and heart sound in acute sleep deprivation individuals. *Sleep Med*. Jan 2024;113:249-259. doi:10.1016/j.sleep.2023.11.040
201. Chee MW, Choo WC. Functional imaging of working memory after 24 hr of total sleep deprivation. *J Neurosci*. May 12 2004;24(19):4560-7. doi:10.1523/JNEUROSCI.0007-04.2004
202. Choo WC, Lee WW, Venkatraman V, Sheu FS, Chee MW. Dissociation of cortical regions modulated by both working memory load and sleep deprivation and by sleep deprivation alone. *NeuroImage*. Apr 1 2005;25(2):579-87. doi:10.1016/j.neuroimage.2004.11.029
203. Chen L, Qi X, Zheng J. Altered Regional Cortical Brain Activity in Healthy Subjects After Sleep Deprivation: A Functional Magnetic Resonance Imaging Study. *Front Neurol*. 2018/08/02/ 2018;9:588. doi:10.3389/fneur.2018.00588
204. Dai XJ, Gong HH, Wang YX, et al. Gender differences in brain regional homogeneity of healthy subjects after normal sleep and after sleep deprivation: a resting-state fMRI study. *Sleep Med*. Jun 2012;13(6):720-7. doi:10.1016/j.sleep.2011.09.019
205. Gao L, Bai L, Zhang Y, et al. Frequency-dependent changes of local resting oscillations in sleep-deprived brain. *PLOS ONE*. 2015/03/23/ 2015;10(3):e0120323. doi:10.1371/journal.pone.0120323
206. Kong D, Liu R, Song L, Zheng J, Zhang J, Chen W. Altered Long- and Short-Range Functional Connectivity Density in Healthy Subjects After Sleep Deprivations. *Front Neurol*. 2018/07/16/ 2018;9:546. doi:10.3389/fneur.2018.00546
207. Zeng B, Zhou J, Li Z, Zhang H, Li Z, Yu P. Altered Percent Amplitude of Fluctuation in Healthy Subjects After 36 h Sleep Deprivation. *Front Neurol*. 2021/01/15/ 2020;11:565025. doi:10.3389/fneur.2020.565025
208. Chen J, Gong X, Wang L, et al. Altered Postcentral Connectivity after Sleep Deprivation Correlates to Impaired Risk Perception: A Resting-State Functional Magnetic Resonance Imaging Study. *Brain Sci*. Mar 20 2023;13(3):514. doi:10.3390/brainsci13030514
209. Dai C, Peng Z, Wang L, et al. Total sleep deprivation reduces the table tennis anticipation performance of young men: A functional magnetic resonance imaging study. *iScience*. Oct 20 2023;26(10):107973. doi:10.1016/j.isci.2023.107973
210. Liu C, Kong XZ, Liu X, Zhou R, Wu B. Long-term total sleep deprivation reduces thalamic gray matter volume in healthy men. *NeuroReport*. Mar 26 2014;25(5):320-3. doi:10.1097/WNR.0000000000000091
211. Shao Y, Qi J, Fan M, et al. Compensatory Neural Responses After 36 Hours of Total Sleep Deprivation and its Relationship with Executive Control Function. *soc behav pers*. 2009/10/01/ 2009;37(9):1239-1249. doi:10.2224/sbp.2009.37.9.1239
212. Czisch M, Wehrle R, Harsay HA, et al. On the Need of Objective Vigilance Monitoring: Effects of Sleep Loss on Target Detection and Task-Negative Activity Using Combined EEG/fMRI. *Front Neurol*. 2012 2012;3:67. doi:10.3389/fneur.2012.00067
213. Dai XJ, Liu CL, Zhou RL, et al. Long-term total sleep deprivation decreases the default spontaneous activity and connectivity pattern in healthy male subjects: a resting-state fMRI study. *Neuropsychiatr Dis Treat*. 2015/03// 2015;11:761-72. doi:10.2147/NDT.S78335
214. Drummond SP, Brown GG, Stricker JL, Buxton RB, Wong EC, Gillin JC. Sleep deprivation-induced reduction in cortical functional response to serial subtraction. *NeuroReport*. Dec 16 1999;10(18):3745-8. doi:10.1097/00001756-199912160-00004
215. Drummond SP, Gillin JC, Brown GG. Increased cerebral response during a divided attention task following sleep deprivation. *J Sleep Res*. Jun 2001;10(2):85-92. doi:10.1046/j.1365-2869.2001.00245.x
216. Drummond SP, Brown GG, Salamat JS, Gillin JC. Increasing task difficulty facilitates the cerebral compensatory response to total sleep deprivation. *Sleep*. May 1 2004;27(3):445-51.
217. Drummond SP, Meloy MJ, Yanagi MA, Orff HJ, Brown GG. Compensatory recruitment after sleep deprivation and the relationship with performance. *Psychiatry Res*. Dec 30 2005;140(3):211-23. doi:10.1016/j.pscychresns.2005.06.007
218. Fischer S, Nitschke MF, Melchert UH, Erdmann C, Born J. Motor memory consolidation in sleep shapes more effective neuronal representations. *J Neurosci*. Dec 7 2005;25(49):11248-55. doi:10.1523/JNEUROSCI.1743-05.2005
219. Gazdzinski SP, Binder M, Bortkiewicz A, Baran P, Dziuda Ł. Effects of All-Night Driving on Selective Attention in Professional Truck Drivers: A Preliminary Functional Magnetic Resonance Study. *Energies*. 2021/08/31/ 2021;14(17):5409. doi:10.3390/en14175409
220. Gazes Y, Rakitin BC, Steffener J, et al. Dual-tasking alleviated sleep deprivation disruption in visuomotor tracking: an fMRI study. *Brain Cogn*. Apr 2012;78(3):248-56. doi:10.1016/j.bandc.2012.01.004
221. Goldstein AN, Greer SM, Saletin JM, Harvey AG, Nitschke JB, Walker MP. Tired and apprehensive: anxiety amplifies the impact of sleep loss on aversive brain anticipation. *J Neurosci*. Jun 26 2013;33(26):10607-15. doi:10.1523/JNEUROSCI.5578-12.2013
222. Gujar N, Yoo SS, Hu P, Walker MP. The unrested resting brain: sleep deprivation alters activity within the default-mode network. *J Cogn Neurosci*. Aug 2010;22(8):1637-48. doi:10.1162/jocn.2009.21331
223. Gujar N, Yoo SS, Hu P, Walker MP. Sleep deprivation amplifies reactivity of brain reward networks, biasing the appraisal of positive emotional experiences. *J Neurosci*. Mar 23 2011;31(12):4466-74. doi:10.1523/JNEUROSCI.3220-10.2011
224. Yoo SS, Hu PT, Gujar N, Jolesz FA, Walker MP. A deficit in the ability to form new human memories without sleep. *Nat Neurosci*. Mar 2007;10(3):385-92. doi:10.1038/nn1851
225. Guo Z, Jiang Z, Jiang B, McClure MA, Mu Q. High-Frequency Repetitive Transcranial Magnetic Stimulation Could Improve Impaired Working Memory Induced by Sleep Deprivation. *Neural Plast*. 2019/12/12/ 2019;2019:7030286. doi:10.1155/2019/7030286
226. Huang NX, Gao ZL, Lin JH, Lin YJ, Chen HJ. Altered stability of brain functional architecture after sleep deprivation: A resting-state functional magnetic resonance imaging study. *Front Neurosci*. 2022/10/13/ 2022;16:998541. doi:10.3389/fnins.2022.998541

© 2025 Reimann GM et al. *JAMA Psychiatry*.

227. Yan FX, Lin JL, Lin JH, Chen HJ, Lin YJ. Altered dynamic brain activity and its association with memory decline after night shift-related sleep deprivation in nurses. *J Clin Nurs*. Jul 2023;32(13-14):3852-3862. doi:10.1111/jocn.16515
228. Jackson ML, Hughes ME, Croft RJ, et al. The effect of sleep deprivation on BOLD activity elicited by a divided attention task. *Brain Imaging Behav*. Jun 2011;5(2):97-108. doi:10.1007/s11682-011-9115-6
229. Klumpers UM, Veltman DJ, van Tol MJ, et al. Neurophysiological effects of sleep deprivation in healthy adults, a pilot study. *PLOS ONE*. 2015/01/21/ 2015;10(1):e0116906. doi:10.1371/journal.pone.0116906
230. Kong D, Soon CS, Chee MW. Functional imaging correlates of impaired distractor suppression following sleep deprivation. *NeuroImage*. May 15 2012;61(1):50-5. doi:10.1016/j.neuroimage.2012.02.081
231. Lei Y, Wang L, Chen P, et al. Neural correlates of increased risk-taking propensity in sleep-deprived people along with a changing risk level. *Brain Imaging Behav*. Dec 2017;11(6):1910-1921. doi:10.1007/s11682-016-9658-7
232. Shen H, Xu H, Wang L, et al. Making group inferences using sparse representation of resting-state functional mRI data with application to sleep deprivation. *Hum Brain Mapp*. Sep 2017;38(9):4671-4689. doi:10.1002/hbm.23693
233. Lythe KE, Williams SC, Anderson C, Libri V, Mehta MA. Frontal and parietal activity after sleep deprivation is dependent on task difficulty and can be predicted by the fMRI response after normal sleep. *Behav Brain Res*. Jul 15 2012;233(1):62-70. doi:10.1016/j.bbr.2012.04.050
234. Mao T, Fang Z, Chai Y, et al. Sleep deprivation attenuates neural responses to outcomes from risky decision-making. *Psychophysiology*. Apr 2024;61(4):e14465. doi:10.1111/psyp.14465
235. Menz MM, Buchel C, Peters J. Sleep deprivation is associated with attenuated parametric valuation and control signals in the midbrain during value-based decision making. *J Neurosci*. May 16 2012;32(20):6937-46. doi:10.1523/JNEUROSCI.3553-11.2012
236. Rihm JS, Menz MM, Schultz H, et al. Sleep Deprivation Selectively Upregulates an Amygdala-Hypothalamic Circuit Involved in Food Reward. *J Neurosci*. Jan 30 2019;39(5):888-899. doi:10.1523/JNEUROSCI.0250-18.2018
237. Mi Y, Duan H, Xu Z, Lei X. The Impact of Sleep Deprivation on Brain Networks in Response to Social Evaluation Tasks. *Brain Sci*. Jul 25 2023;13(8):1122. doi:10.3390/brainsci13081122
238. Mu Q, Mishory A, Johnson KA, et al. Decreased brain activation during a working memory task at rested baseline is associated with vulnerability to sleep deprivation. *Sleep*. Apr 2005;28(4):433-46. doi:10.1093/sleep/28.4.433
239. Mu Q, Nahas Z, Johnson KA, et al. Decreased cortical response to verbal working memory following sleep deprivation. *Sleep*. Jan 2005;28(1):55-67. doi:10.1093/sleep/28.1.55
240. Mullin BC, Phillips ML, Siegle GJ, Buysse DJ, Forbes EE, Franzen PL. Sleep deprivation amplifies striatal activation to monetary reward. *Psychol Med*. Oct 2013;43(10):2215-25. doi:10.1017/S0033291712002875
241. Nakashima A, Bouak F, Lam Q, Smith I, Vartanian O. Task switching following 24 h of total sleep deprivation: a functional MRI study. *NeuroReport*. Jan 17 2018;29(2):123-127. doi:10.1097/WNR.0000000000000934
242. Shermohammed M, Kordyban LE, Somerville LH. Examining the Causal Effects of Sleep Deprivation on Emotion Regulation and Its Neural Mechanisms. *J Cogn Neurosci*. Jul 2020;32(7):1289-1300. doi:10.1162/jocn_a_01555
243. Sun J, Zhao R, Yang X, et al. Alteration of Brain Gray Matter Density After 24 h of Sleep Deprivation in Healthy Adults. *Front Neurosci*. 2020/08/11/ 2020;14:754. doi:10.3389/fnins.2020.00754
244. Zhao R, Zhang X, Fei N, et al. Decreased cortical and subcortical response to inhibition control after sleep deprivation. *Brain Imaging Behav*. Jun 2019;13(3):638-650. doi:10.1007/s11682-018-9868-2
245. Thomas M, Sing H, Belenky G, et al. Neural basis of alertness and cognitive performance impairments during sleepiness. I. Effects of 24 h of sleep deprivation on waking human regional brain activity. *J Sleep Res*. Dec 2000;9(4):335-52. doi:10.1046/j.1365-2869.2000.00225.x
246. Vartanian O, Bouak F, Caldwell JL, et al. The effects of a single night of sleep deprivation on fluency and prefrontal cortex function during divergent thinking. *Front Hum Neurosci*. 2014/04/22/ 2014;8:214. doi:10.3389/fnhum.2014.00214
247. Venkatraman V, Chuah YM, Huettel SA, Chee MW. Sleep deprivation elevates expectation of gains and attenuates response to losses following risky decisions. *Sleep*. May 2007;30(5):603-9. doi:10.1093/sleep/30.5.603
248. Venkatraman V, Huettel SA, Chuah LY, Payne JW, Chee MW. Sleep deprivation biases the neural mechanisms underlying economic preferences. *J Neurosci*. Mar 9 2011;31(10):3712-8. doi:10.1523/JNEUROSCI.4407-10.2011
249. Wang L, Chen Y, Yao Y, Pan Y, Sun Y. Sleep deprivation disturbed regional brain activity in healthy subjects: evidence from a functional magnetic resonance-imaging study. *Neuropsychiatr Dis Treat*. 2016/04// 2016;12:801-7. doi:10.2147/NDT.S99644
250. Wu JC, Gillin JC, Buchsbaum MS, et al. Frontal lobe metabolic decreases with sleep deprivation not totally reversed by recovery sleep. *Neuropsychopharmacol*. Dec 2006;31(12):2783-92. doi:10.1038/sj.npp.1301166
251. Xu J, Zhu Y, Fu C, et al. Frontal metabolic activity contributes to individual differences in vulnerability toward total sleep deprivation-induced changes in cognitive function. *J Sleep Res*. Apr 2016;25(2):169-80. doi:10.1111/jsr.12354
252. Zhu Y, Feng Z, Xu J, et al. Increased interhemispheric resting-state functional connectivity after sleep deprivation: a resting-state fMRI study. *Brain Imaging Behav*. Sep 2016;10(3):911-9. doi:10.1007/s11682-015-9490-5
253. Ye H, Ji M, Wang C, et al. Integrated Functional Neuroimaging, Monoamine Neurotransmitters, and Behavioral Score on Depressive Tendency in Intensive Care Unit Medical Staffs Induced by Sleep Deprivation After Night Shift Work. *Front Psychiatry*. 2022/03/22/ 2022;13:848709. doi:10.3389/fpsyt.2022.848709
254. Ye HT, Lu CQ, Wang C, et al. Plasma Abeta level alterations after sleep deprivation correspond to brain structural remodeling in medical night shift workers. *Brain Res Bull*. Oct 15 2023;203:110776. doi:10.1016/j.brainresbull.2023.110776
255. Alsameen M, DiFrancesco MW, Drummond SPA, Franzen PL, Beebe DW. Neuronal activation and performance changes in working memory induced by chronic sleep restriction in adolescents. *J Sleep Res*. Oct 2021;30(5):e13304. doi:10.1111/jsr.13304

© 2025 Reimann GM et al. *JAMA Psychiatry*.

256. DiFrancesco MW, Alsameen M, St-Onge MP, Duraccio KM, Beebe DW. Altered neuronal response to visual food stimuli in adolescents undergoing chronic sleep restriction. *Sleep*. Apr 12 2024;47(4):zsad036. doi:10.1093/sleep/zsad036
257. Demos KE, Sweet LH, Hart CN, et al. The Effects of Experimental Manipulation of Sleep Duration on Neural Response to Food Cues. *Sleep*. Nov 1 2017;40(11)doi:10.1093/sleep/zsx125
258. Li XY, Yoncheva Y, Yan CG, Castellanos FX, St-Onge MP. Chronic Mild Sleep Restriction Does Not Lead to Marked Neuronal Alterations Compared With Maintained Adequate Sleep in Adults. *J Nutr*. Feb 2024;154(2):446-454. doi:10.1016/j.tjnut.2023.12.016
259. Peters AC, Blechert J, Samann PG, Eidner I, Czisch M, Spoormaker VI. One night of partial sleep deprivation affects habituation of hypothalamus and skin conductance responses. *J Neurophysiol*. Sep 15 2014;112(6):1267-76. doi:10.1152/jn.00657.2013
260. Poudel GR, Innes CR, Jones RD. Distinct neural correlates of time-on-task and transient errors during a visuomotor tracking task after sleep restriction. *NeuroImage*. Aug 15 2013;77:105-13. doi:10.1016/j.neuroimage.2013.03.054
261. Robinson JL, Erath SA, Kana RK, El-Sheikh M. Neurophysiological differences in the adolescent brain following a single night of restricted sleep - A 7T fMRI study. *Dev Cogn Neurosci*. Jun 2018;31:1-10. doi:10.1016/j.dcn.2018.03.012
262. St-Onge MP, McReynolds A, Trivedi ZB, Roberts AL, Sy M, Hirsch J. Sleep restriction leads to increased activation of brain regions sensitive to food stimuli. *Am J Clin Nutr*. Apr 2012;95(4):818-24. doi:10.3945/ajcn.111.027383
263. St-Onge MP, Wolfe S, Sy M, Shechter A, Hirsch J. Sleep restriction increases the neuronal response to unhealthy food in normal-weight individuals. *Int J Obes (Lond)*. Mar 2014;38(3):411-6. doi:10.1038/ijo.2013.114

© 2025 Reimann GM et al. *JAMA Psychiatry*.
