# Supplementary material for: Distinct Convergent Brain Alterations in Sleep Disorders and Sleep Deprivation: A Meta-Analysis
Source: JAMA Psychiatry. 2025 Apr 23;82(7):681–91. doi: 10.1001/jamapsychiatry.2025.0488 (PMC12019678; doi:10.1001/jamapsychiatry.2025.0488)
Supplement: Supplement 2. — Data Sharing Statement [file jamapsychiatry-e250488-s002.pdf]

Data Sharing Statement

Reimann. Distinct Convergent Brain Alterations in Sleep Disorders and Sleep Deprivation. *JAMA Psychiatry*. Published April 23, 2025. doi:10.1001/jamapsychiatry.2025.0488

Data
Data available: Yes
Data types: Data (not involving human participants)
How to access data: Coordinates of brain abnormalities across existing studies
When available: With publication

Supporting Documents
Document types: Statistical/analytic code
How to access documents: The coordinate table for the ALE analyses as well as the specific pyALE input are available at https://osf.io/2w5v9/
When available: With publication

Additional Information
Who can access the data: The data is publicly available.
Types of analyses: Neuroimaging meta-analysis
Mechanisms of data availability: With investigator support and after approval of a proposal.
